# Oncogenic KRAS-driven type I interferon signalling primes pancreatic cancer for necroptosis

**DOI:** 10.1038/s41467-026-73189-8

**Published:** 2026-06-15

**Authors:** Sofya Tishina, Alina Dahlhaus, Marta Manik, Lejla Mulalic, Janine Murr, Michael Kotliar, Hassan Rakhsh-Khorshid, Myrto Kostopoulou, Florian Hocher, Jenny Stroh, Julia Beck, Riley M. Williams, Gülce G. Balta, Fanyu Liu, Ali T. Abdallah, Christina M. Bebber, Moritz Reese, Jonathan K. M. Lim, Alexander Quaas, Johannes Brägelmann, Manolis Pasparakis, Filippo Beleggia, Siddharth Balachandran, Anna Trauzold, Gianmaria Liccardi, Igor Astsaturov, Maximilian Reichert, Ariadne Androulidaki, Silvia von Karstedt

**Affiliations:** 1 University of Cologne, Faculty of Medicine and University Hospital Cologne, Department of Translational Genomics, Cologne, Germany; 2 CECAD Cluster of Excellence, Faculty of Medicine and University Hospital Cologne, Cologne, Germany; 3 Translational Pancreatic Cancer Research Centre, TUM School of Medicine and Health, Department of Clinical Medicine – Clinical Department for Internal Medicine II, TUM University Hospital, Technical University of Munich, Munich, Germany; 4 Centre for Organoid Systems (COS), Technical University Munich (TUM), Garching, Germany; 5 Cincinnati Children’s Hospital Medical Center, Division of Allergy and Immunology, Cincinnati, OH USA; 6 University of Cologne, Faculty of Medicine and University Hospital Cologne, Centre for Biochemistry, Cologne, Germany; 7 University of Kiel, Institute for Experimental Cancer Research, Kiel, Germany; 8 Fox Chase Cancer Center, Cancer Signaling and Microenvironment Program, Philadelphia, PA USA; 9 Institute of Medical Statistics and Computational Biology, Faculty of Medicine, University of Cologne, Cologne, Germany; 10 Heinrich Heine University, Medical Faculty and University Hospital Düsseldorf, Institute of Neuropathology, Düsseldorf, Germany; 11 University of Cologne, Faculty of Medicine and University Hospital Cologne, Institute of Pathology, Cologne, Germany; 12 University Hospital Cologne, Mildred Scheel School of Oncology, Cologne, Germany; 13 University Hospital Cologne, Centre for Molecular Medicine Cologne (CMMC), Cologne, Germany; 14 University of Cologne, Institute for Genetics, Cologne, Germany; 15 University Hospital Schleswig-Holstein (UKSH), Department of Gynecology and Obstetrics, Campus Kiel, Kiel, Germany; 16 Fox Chase Cancer Center, Molecular Therapeutics Program, Philadelphia, PA USA; 17 German Cancer Consortium (DKTK), partner site Munich, a partnership between DKFZ and TUM University Hospital, Munich, Germany; 18 Bavarian Cancer Research Centre (BZKF), Munich, Germany

**Keywords:** Necroptosis, Pancreatic cancer

## Abstract

Pancreatic ductal adenocarcinoma (PDAC) is projected to become the second leading cause of cancer-related death within this decade. Here, we show that its major driver oncogene KRAS activates the cGAS-STING-TBK1 axis, inducing a type I interferon (IFN) response that primes PDAC cells for necroptosis. Using genetically engineered mouse models, we find that cancer cell-specific deletion of caspase-8 is sufficient to trigger necroptotic cell death, eliminating most pancreatic precursor lesions. Mechanistically, KRAS-driven IFN signalling induces ISGF3-dependent expression of necroptosis-related interferon-stimulated genes, including MLKL. This renders PDAC cells selectively vulnerable to necroptosis upon caspase-8 inhibition. Therapeutically, pharmacologic caspase inhibition reduces tumour burden in aggressive PDAC models and human patient-derived organoids. A pan-cancer transcriptomic analysis links necroptosis gene expression with Ras pathway activity and IFN signatures across multiple tumour types. These findings reveal a KRAS-induced IFN program that sensitises tumour cells to necroptosis, highlighting a therapeutic vulnerability in PDAC with broader relevance across IFN-activated cancers.

## Abstract (web summary)

The regulatory roles of oncogenic KRAS in cell death pathways remain poorly understood. Here the authors report that KRAS induces a type I interferon response that primes pancreatic cancer cells for necroptosis, rendering a therapeutic vulnerability to inhibition of caspase 8.

## Introduction

Pancreatic ductal adenocarcinoma (PDAC) is among the deadliest cancer entities, and it is expected to become the second leading cause of cancer-related death within this decade^1–3^. Large research efforts of recent years have described the existence of several molecular subtypes of PDAC^4–7^ indicating that intra- and interpatient heterogeneity likely complicates effective treatment further. 90% of PDAC cases present with activating point mutations within the proto-oncogene and small GTPase KRAS leading to its constitutive activation and aberrant downstream signalling^8^. While several studies have identified promising synthetic lethality targets for cells expressing oncogenic KRAS^9^, many of these are cytostatic rather than inducing regulated cell death pathways. Notably, expression of oncogenic KRAS protects against oxidative cell death and ferroptosis via upregulation of the cystine/glutamate antiporter xCT^10^ and ferroptosis suppressor protein 1 (FSP1), respectively^11^. Moreover, cells expressing oncogenic KRAS are known to rewire the extrinsic apoptosis pathway leading to extrinsic apoptosis resistance^12,13^. Extrinsic apoptosis is triggered through death receptors belonging to the tumour necrosis factor (TNF) receptor (TNFR)-superfamily^14^. Upon ligand binding to their cognate receptor, the adaptor molecule Fas associated with a death domain (FADD) is recruited allowing for pro-caspase 8 recruitment and its proximity-induced activation^15^. The activity of this death-inducing signalling complex (DISC)^16^ critically depends upon caspase 8 for effective execution of extrinsic apoptosis in mice. Importantly, during mouse development and in healthy adult tissue homeostasis, caspase 8 also protects cells from aberrant necroptosis^17–19^, a non-apoptotic form of cell death driven by RIPK1, RIPK3^20,21^, and MLKL^19^. Despite this essential role in mouse development, homozygous caspase 8 mutations in amino acid 248 in humans are compatible with normal development but lead to impaired lymphocyte development and immunodeficiency^22^. While this mutation found in humans led to decreased expression levels and impaired enzymatic apoptotic activity some of its other pleiotropic functions might have been preserved. While its role in normal tissue homeostasis has been the subject of intense investigation, the role of caspase 8 in neoplastic disease remains controversial and both up- and downregulation has been observed. In cancers with neuroendocrine differentiation caspase 8 expression is characteristically low and this has been suggested to promote metastasis by disabling apoptosis and, more recently, by promoting necroptosis-fuelled inflammation^23–29^. By contrast, high nuclear caspase 8 has been shown to promote melanoma by protecting from p53-driven apoptosis^30^. Moreover, caspase 8 expression fulfils a central role in promoting liver injury- and inflammation-associated hepatocarcinogenesis^31,32^. Previous studies have reported elevated expression of necroptosis pathway components such as RIPK1, RIPK3, and MLKL in human PDAC^33,34^ suggesting a cancer-protective function of caspase 8 expression in this context. However, the upstream mechanisms driving this expression and the functional consequences for tumour development remain poorly understood.

Here, we identify a mechanism by which oncogenic KRAS activates type I interferon signalling. A cytosolic DNA sensing cascade involving cGAS and STING drives the upregulation of necroptosis effectors upon expression of oncogenic KRAS and establishes a genetic dependency on caspase 8 to prevent necroptotic cell death. These findings uncover a link between KRAS-induced innate immune activation and cell death pathway regulation in PDAC, and they reveal type I interferon signalling as a potential vulnerability to necroptosis-inducing therapies.

## Results

### KRAS-driven pancreatic neoplasia is vulnerable to genetic induction of necroptosis

**Fig. 1:**
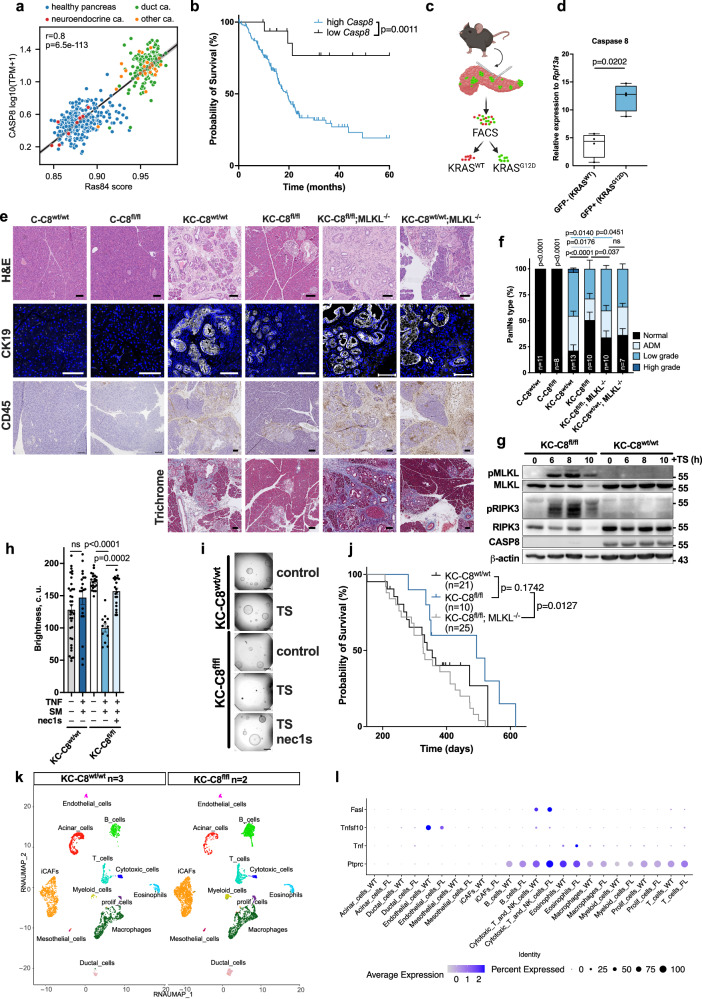
KRAS-driven pancreatic neoplasia is vulnerable to genetic induction of necroptosis. **a** Caspase 8 expression plotted as a function of the Ras84 score^38^ in the PAAD TCGA and normal human pancreas (GTEx) datasets. CA carcinoma. **b** Kaplan–Meier survival of the 10th percentile lowest *Casp8* expression plotted vs. the rest of PAAD TCGA. **c** Schematic of GFP^+^ and tdTomato^+^ cells isolation from KC-mice pancreata with flow cytometry sorting was created with Biorender. JS (2025) (https://BioRender.com/ffudhn3). **d** Relative expression of C*asp8* from adjacent WT pancreas (tdTomato^+^, *n* = 4) and KRAS^G12D^-expressing PanINs (GFP^+^, *n* = 4) from 5-month-old KC mice. Boxplot centre line, mean; box limits, upper/lower quartile; whiskers min. to max. **e**, **f** 5-month-old mice of the indicated genotypes were sacrificed and the pancreata were stained by H&E, CK19, CD45 or trichrome. **e** Representative images are shown. Size bars represent 100 µm. **f** 5-month-old mice from the indicated genotypes pancreas stained by H&E and ducts were graded and quantified by blinded pathological inspection. Additional significantly different *p*-values that are not in figure (in normal PanINs): KC-C8^fl/fl^ vs KC-C8^wt/wt^; MLKL^−/−^ (*p* = 0.0239) and KC-C8^wt/wt^ vs KC-C8^fl/fl^; MLKL^−/−^ (*p* = 0.0185) and KC-C8^wt/wt^; MLKL^−/−^ (*p* = 0.0118). **g** Organoids from 5-month-old KC-C8^wt/wt^ and KC-C8^fl/fl^ mice treated with TNF [500 ng/ml], SMAC mimetics (SM, [1 µM]). Harvested at indicated time points. Representative Western blots are shown. **h**, **i** Organoids were treated for 24 h with TNF [500 ng/ml], SM [1 µM], and nec1s [10 µM]. **h** Organoid viability was quantified using organoid brightness as a proxy. *n* = 35, 20, 21, 12, 21 independent organoids from 3 repeats (left to right). **i** Images of representative organoids are shown. **j** Kaplan–Meier survival for mice of indicated genotypes is shown. **k** Single cells from 5-month-old KC-C8^wt/wt^ and KC-C8^fl/fl^ were subjected to scRNA-seq and UMAP plots are shown. **l** Cell type-specific expression of the indicated genes are shown as log1p-transformed scaled average gene expression (scale factor 10,000) within KC-C8^wt/wt^ (WT) and KC-C8^fl/fl^ (FL) single cells. Data are means +/− SEM (**d**, **f**, **h**). Two-way ANOVA (**f**), one-way ANOVA + Sidak’s test (**h**), logrank test (**b**, **j**) and paired *t* test (**d**) were used. Source data are provided as a Source Data file.

All human cancers with activating point mutations in the small GTPase KRAS within the Cancer Genome Atlas (TCGA) highly express caspase 8 (Supplementary Fig. 1a). These include lung adenocarcinoma (LUAD), colon adenocarcinoma (COAD) and PDAC, (PAAD in TCGA). Interestingly, caspase 8 mRNA expression was also elevated in PDAC as compared to adjacent normal pancreas in two independent patient cohorts^35,36^ (Supplementary Fig. 1b, c) and in a recent proteogenomic study^37^. Overall, these data hint at an unrecognised cancer-beneficial function of caspase 8 expression in several cancer entities in general and in the context of oncogenic KRAS-driven neoplasia in particular. To investigate this function, we first correlated caspase 8 expression in human PDAC and normal pancreatic tissue using respective datasets from TCGA (PAAD) and the Genotype Tissue Expression project (GTEx) with a recently published score for transcriptional RAS pathway activation (Ras84)^38^. Indeed, caspase 8 expression strongly correlated with the Ras84 score in PDAC while normal pancreas showed significantly less caspase 8 and Ras84 score expression (Fig. 1a). Of note, the few cases of neuroendocrine pancreatic cancers contained within the PAAD dataset expressed explicitly low levels of caspase 8 and Ras84 score. Moreover, the 10% of PDAC patients with lowest caspase 8 expression constituted a group of PDAC super-survivors (Fig. 1b). To genetically define the function of caspase 8 in KRAS-driven PDAC, we first addressed whether PDAC-specific caspase 8 upregulation as compared to adjacent normal pancreas was recapitulated within the most used genetically engineered mouse model expressing oncogenic KRAS^G12D^ targeted to pancreatic precursor cells (KC-mice) resulting in pancreatic intraepithelial neoplasia (PanIN) development^39^. To this end, we crossed KC-mice to a reporter strain in which all tissues express tandem tomato (tdTomato) which switch to expression of green fluorescent protein (GFP) upon Cre-mediated excision (ROSA26^mTmG^-mice)^40^. As expected, macroscopic pancreata showed mosaic tdTomato^+^ and GFP^+^ areas and microscopic inspection confirmed these to be mutually exclusive (Supplementary Fig. 1d). Next, pancreatic tissues were digested, and GFP^+^ and GFP^-^ cells were sorted by fluorescent-activated cell sorting (Fig. 1c, Supplementary Fig. 1e) and subjected to analyses by quantitative real-time PCR (qPCR). Strikingly, GFP^+^ PanIN cells expressing KRAS^G12D^ from four individual mice indeed expressed significantly elevated levels of caspase 8 mRNA as compared to adjacent normal tissue (Fig. 1d). Next, to approach a function of caspase 8 in pancreatic tissue before and after oncogenic transformation, we first generated mice with mosaic deletion of caspase 8 in normal pancreatic precursor cells, PDX1-Cre(C); Casp8^fl/fl^(C8^fl/fl^). Surprisingly, and unlike in the skin and intestine^41,42^, C-C8^fl/fl^ mice did not show any overt signs of inflammatory disease of the pancreas or changes in CD45^+^ immune infiltrates (Fig. 1e, Supplementary Fig. 1f). Therefore, given the observed high caspase 8 expression within KRAS-mutated TCGA cancer cohorts, we crossed C8^fl/fl^ mice with KC-mice. Strikingly, PanIN-restricted caspase 8 deletion led to a drastic amelioration of PanIN development in KC-mice with ~50% of pancreatic ducts presenting with normal morphology (Fig. 1e, f). Of note, caspase 8 protein was also upregulated within PanINs as compared to adjacent normal pancreas in tissue stainings of KC-mice and effectively depleted in KC-C8^fl/fl^ lesions (Supplementary Fig. 1h). Notably, during mouse development, caspase 8 is known to protect cells from aberrant necroptosis^20,21^, while its role in neoplastic disease is less clear. Therefore, we next tested whether the reduced disease burden observed was genetically dependent upon the necroptosis executioner MLKL. To this end, we crossed KC-C8^fl/fl^ mice with MLKL-deficient mice^43^. Strikingly, MLKL-deficiency fully reverted PanIN amelioration observed in KC-mice upon conditional caspase 8 deletion (Fig. 1e, f). Unlike the results from blinded pathological inspection (Fig. 1f), automated quantification of the Cytokeratin 19 (CK19) ^+^ areas showed only partial reversal by MLKL-deficiency (Supplementary Fig. 1g). This was probably caused by the fact that very large ductal lesions with very thin CK19^+^ ductal linings were observed in KC-C8^fl/fl^; MLKL^−/−^ mice, a feature which is frequently seen upon progression and loss of epithelial differentiation (Fig. 1e, CK19 panel). A hallmark feature of necroptotic cell death is phosphorylation of MLKL^44,45^. Nevertheless, we could not detect any pMLKL using a recently published protocol^46^ and terminal deoxynucleotidyl transferase (TdT) dUTP nick end labeling positive (TUNEL + ) cells were at the background level of death at time of tissue harvest, most likely due to earlier clearance of dead cells resulting in less PanINs at time of analysis (Supplementary Fig. 1i). For this reason, we isolated murine organoids from KC-C8^fl/fl^ and KC-C8^wt/wt^ animals and stimulated them ex vivo with TNF (T) and the SMAC mimetic birinapant (S), a combination known to only trigger necroptosis when caspase 8 is inactivated^47^. Indeed, we observed a time dependent strong phosphorylation of MLKL and RIPK3 only in KC-C8^fl/fl^ organoids (Fig. 1g). Moreover, cell death in KC-C8^fl/fl^ organoids, but not wild type controls, was fully reverted by co-treatment with the RIPK1 inhibitor nec1s, indicating the induction of necroptotic cell death (Fig. 1h, i).

In addition to restricting PanIN formation, upon necropsy at experimental endpoint we noted a drastically increased occurrence of macroscopic liver metastasis in 68% of KC-C8^fl/fl^; MLKL^−/−^ as opposed to 38.5% in KC-C8^wt/wt^ and only 28.6% in KC-C8^fl/fl^ mice (Supplementary Fig. 1j, k). These data suggest that in addition to PanIN development, pancreatic cancer metastases are constitutively edited via the necroptosis and apoptosis pathway, a feature only evident when both compensatory pathways are taken out. In line with this, KC-C8^fl/fl^ mice only showed a significant increase in overall survival in comparison to KC-C8^fl/fl^; MLKL^−/−^ (Fig. 1j). Moreover, we found that heterozygous KC-C8^wt/fl^ mice which have reduced extrinsic apoptotic activity but retain caspase 8-mediated blockade of necroptosis^21^ show an exacerbated disease phenotype reminiscent of KC-C8^fl/fl^; MLKL^−/−^ mice (Supplementary Fig. 1l, m).

In contrast to our results using MLKL-deficient mice, a previous study has shown that whole body RIPK3-deficiency in KC-mice ameliorates PanIN progression^34^. Based upon these data it was concluded that necroptosis-associated inflammation promotes pancreatic oncogenesis. In order to account for this discrepancy, we also crossed KC-mice to RIPK3^−/−^ mice^48^ and could confirm that whole-body RIPK3-deficiency indeed led to disease amelioration (Supplementary Fig. 1l, m). Moreover, KC-C8^fl/fl^; RIPK3^−/−^ mice did not show additional disease amelioration in comparison to KC-RIPK3^−/−^ mice (Supplementary Fig. 1m). These findings suggest that caspase 8 deletion has no additive effect in the absence of RIPK3, supporting a model in which disease amelioration observed in KC-RIPK3^−/−^ mice is mediated via non-necroptotic functions of RIPK3^49–51^ and that caspase 8 deletion can only elicit a phenotype in the presence of RIPK3. Notably, MLKL-deficiency specifically abrogates necroptosis in mouse models of tissue injury^52^.

Necroptosis can be induced by TNF-superfamily death ligand stimulation including TNF, CD95L or TRAIL all of which are known to be expressed by activated immune cells while TRAIL was also suggested to be expressed by KRAS-mutated cancer cells^13,15^. To map death ligand expression across neoplastic and normal pancreatic tissue, we next analysed the transcriptomes of single cells (sc) isolated from 5-month-old KC-pancreata with or without caspase 8 deletion. After initial quality control and pooled analysis batch correction^53^, we obtained transcriptomes from 5,356 cells from KC-C8^wt/wt^ (3 mice) and 8,251 cells from KC-C8^fl/fl^ (2 mice) pancreata. Overall, dimensionality reduction using Uniform Manifold Approximation and Projection (UMAP) identified 12 cell clusters in all replicates whose cellular identity was inferred from established pancreatic lineage markers^54,55^ (Supplementary Data 1). While pancreatic acinar, ductal, mesothelial and inflammatory cancer-associated fibroblasts (iCAFs) could be detected, a significant proportion of single cells constituted immune cells in both genotypes (Fig. 1k). Interestingly, and irrespective of caspase 8 deletion within PanINs, we found TNF to be widely expressed by innate immune cells, CD95L expression was restricted to the T- and effector cell compartment and TRAIL was in addition also expressed by endothelial and ductal cells supporting a possibility of autocrine stimulation^13^ (Fig. 1l). The presence of soluble death ligand protein expression could also be confirmed using ELISA on whole pancreas extracts from KC-mice (Supplementary Fig. 2a–c).

To further study differentiation state within PanIN cells upon caspase 8 deletion we also focussed on the non-immune compartment (CD45^−^ cells). Indeed, highest caspase 8 expression was detected within ductal cells containing the PanIN cell fraction and was downregulated in the majority but not all KC-C8^fl/fl^ ductal cells (Supplementary Fig. 2d). Ductal cells from KC-C8^fl/fl^ mice also showed significantly lower expression of the transcription factor *Sox9*- a known driver of acinar-to-ductal cell reprogramming^56^ (Supplementary Fig. 2e) supporting the presence of less progressed PanINs in the absence of caspase 8.

In line with the histology data (Fig. 1e, Supplementary Fig. 1f), the overall amount of CD45^+^ cells within the scRNA-seq dataset did not differ between KC-C8^wt/wt^ (30.6%) and KC-C8^fl/fl^ (32%) yet differences within individual immune cell clusters were evident from the initial UMAP analysis (Fig. 1k). To further resolve these differences, we next re-clustered CD45^+^ cells only and identified 11 distinct clusters in all samples whose cellular identity was inferred from published mouse immune cell markers for scRNA-seq data^57^ (Supplementary Fig. 2f, g, Supplementary Data 2).

Neutrophils are known to infiltrate sites of cell death within tissues^58^ in particular in the context of necroptotic cell death^59^. Differential gene expression analysis within the neutrophil/eosinophil cluster indeed revealed genes associated with neutrophil activation such as CCL3 to be highly enriched in the KC-C8^fl/fl^ group (Supplementary Fig. 2h). Moreover, we could also detect elevated levels of CXCL2 and CCL3 in bulk RNA and on protein levels in KC-C8^fl/fl^ mice (Supplementary Fig. 2i, j). Significantly elevated levels of CXCL2 mRNA and protein were also reverted by additional MLKL-deficiency (Supplementary Fig. 2i, j). Given that at time of sampling (5 months), KC-C8^fl/fl^ mice had already less PanINs and some of the differential immune response might have subsided, we also profiled immune infiltrates at 3 months of age, a time at which pancreata showed minimal PanIN development in both groups (Supplementary Fig. 2k). Interestingly, pancreata of KC-C8^fl/fl^ mice contained significantly reduced levels of M2-macrophages, (Supplementary Fig. 2l, m), elevated levels of neutrophils (CD14-CCR3−/CD11b+SSC^high^/Gr1+ cells) (Supplementary Fig. 2l, m) and enhanced levels of inflammatory chemokines and cytokines indicative of innate immune activation (Supplementary Fig. 2n). Moreover, we could trace neutrophils/eosinophils as the main source of CXCL2 as detected in bulk also on single-cell level (Supplementary Fig. 2o).

Since caspase 10 (CASP10) can function redundantly with caspase 8 to suppress necroptosis in humans, we also examined its expression in human PDAC. Analysis of the TCGA PAAD dataset showed that caspase 10 expression is indeed also significantly elevated in human PDAC and correlates strongly with the Ras84 score (*r* = 0.85), while normal pancreatic tissue expresses much lower levels of CASP10 (Supplementary Fig. 2p). Moreover, patients within the lowest decile of caspase 10 expression exhibited markedly prolonged survival (Supplementary Fig. 2q). These observations independently indicate that elevated necroptosis suppressors, including CASP10 in humans, may support PDAC maintenance by limiting necroptotic cell death. Taken together, we provide genetic evidence that caspase 8 deletion in the pancreas promotes disease-restrictive necroptosis in the context of oncogenic-KRAS driven neoplasia.

### Expression of oncogenic KRAS induces interferon-stimulated genes (ISGs)

**Fig. 2:**
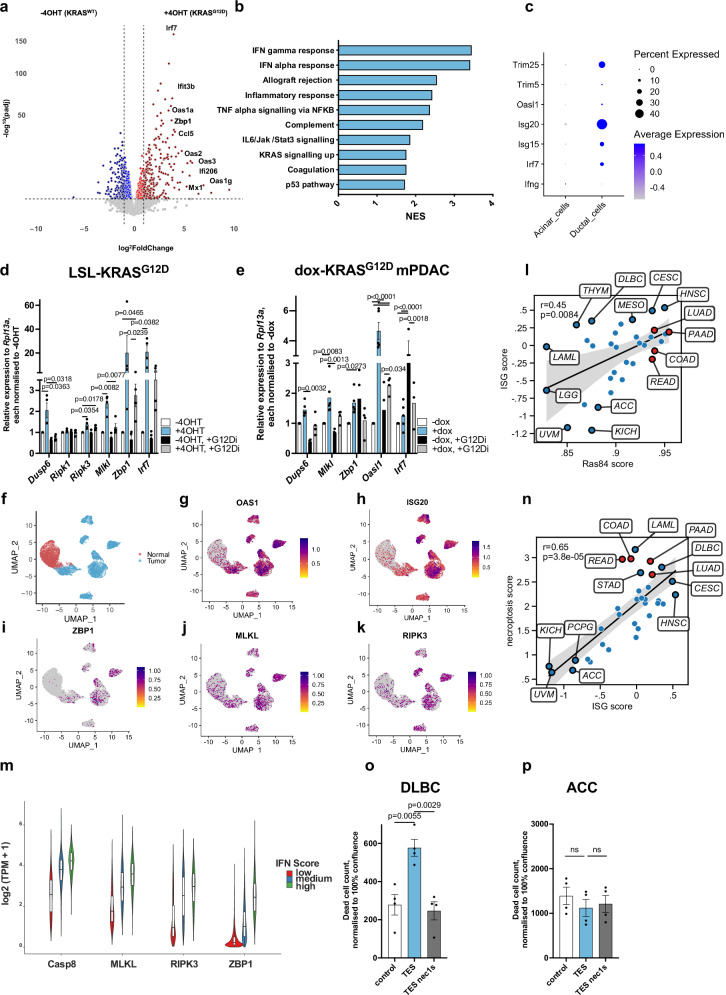
Expression of oncogenic KRAS induces interferon-stimulated genes (ISGs). **a** Seven LSL-KRAS^G12D^; mTmG MEF lines generated from 3 distinct litters were treated with control or 4OHT [1 µM] for 96 h. RNA-sequenced. A volcano plot shows the top 20 upregulated transcripts. red = +4OHT (KRAS^G12D^), blue = −4OHT (KRAS^WT^). **b** A ranked list from the RNA-seq data from (**a**) was subjected to gene set enrichment analysis (GSEA). Significant normalized enrichment score (NES) is shown for all hallmark gene sets within the KRAS^G12D^-induced group ( + 4OHT). **c** Expression of the indicated genes comparing acinar (normal) versus ductal (KRAS^G12D^ PanIN cells) within scRNA-seq. Expression is shown as log1p-transformed scaled average gene expression (scale factor 10000) within KC-C8^wt/wt^ sc (*n* = 3)**. d** LSL-KRAS^G12D^ MEFs (*n* = 4) as in a were treated with 4OHT [1 µM] and KRAS^G12D^ inhibitor (G12Di) [10 nM] for 96 h. Gene expression was quantified by qPCR. **e** Mouse dox-KRAS^G12D^ mPDAC from KC mice were treated with doxycycline [1 µM] and G12Di [20 nM] for 24 h. The exact n numbers for each condition are in the Source Data file. **f**–**k** UMAP plots of necroptosis-related genes in primary human PDAC and normal pancreas scRNA-Seq data^67^. **l**, An ISG score^68^ was computed as published and plotted against the Ras84 signature^38^. **m** An ISG score as in l was plotted against gene expression of *Casp8*, *Mlkl*, *Ripk3* and *Zbp1* within cancers across all human TCGA datasets with high, intermediate and low ISG score (*n* = 1531, 6793, 1464 patient samples, respectively, across 32 TCGA cancer types). Boxplot centre line, mean; box limits, upper/lower quartile; whiskers min. to max. **n** A necroptosis score was computed upon combined *Ripk3*, *Mlkl* and *Zbp1* expression and correlated to an ISG score^68^. Red dots - cancers with frequent KRAS mutations. **o**, **p** Diffuse large B cell lymphoma (DLBC, *n* = 4) (**o**), Adrenocortical Carcinoma (ACC, *n* = 4) (**p**) cells were treated with (SM, birinapant [1 μM], Emricasan [2.5 μM], nec1s [10 μM], TNF [50 ng/ml]). Cell death was analysed by DRAQ7 fluorescence using the IncuCyte bioimaging. Data are means +/− SEM. Two-way ANOVA (**d**, **e**) and one-way ANOVA (**o**, **p**) were used. Source data are provided as a Source Data file.

The observation that genetic induction of necroptosis had a more profound effect in the context of oncogenic KRAS-driven transformation in vivo, led us to investigate whether cells expressing oncogenic KRAS might selectively regulate the necroptosis pathway. To address this question in a simplified cellular system with oncogenic KRAS expression from the endogenous locus we generated mouse embryonic fibroblasts (MEFs) from a cross of an inducible Cre strain (CAG-ERT2-Cre) with the LSL-KRAS^G12D^ mouse strain and mTmG reporter mice as previously described^11^ (hereafter referred to as LSL-KRAS^G12D^ MEFs). Upon treatment with 4-hydroxytamoxifen (4OHT), tdTomato+ MEFs switched to express GFP indicating successful Cre-mediated recombination and KRAS^G12D^ expression (Supplementary Fig. 3a). Next, we subjected LSL-KRAS^G12D^ MEFs derived from 7 independent embryos from 3 distinct litters to treatment with or without 4OHT and bulk RNA-sequencing. Interestingly, expression of endogenous KRAS^G12D^ strongly induced a list of bona fide interferon-stimulated genes (ISGs) including *Irf7*, *Oasl1*, *Ccl5*, *Zbp1*, *Mx1* and *Ifi206* (Fig. 2a). Gene set enrichment analysis (GSEA) confirmed a strong enrichment of type I and II interferon (IFN) pathway activation along with NF-kB activation, IL-6/STAT3 and KRAS signalling activation signatures (Fig. 2b). In support of this, all bona fide ISGs tested were much more strongly expressed within the ductal cell (KRAS-driven PanIN) but not acinar cell (normal) population within KC-derived single cells (Fig. 2c). Notably, the two core necroptosis executioners *Zbp1* and *Mlkl* are well characterised ISGs^60–63^. Indeed, expression of *Zbp1* and *Mlkl* with the known KRAS effector pathway target gene *Dusp6* was significantly induced upon induction of KRAS^G12D^ (Fig. 2d, Supplementary Fig. 3b). Importantly, this induction was dependent upon KRAS^G12D^ activity as co-treatment with MRTX1133 – a recently developed selective inhibitor for KRAS^G12D^^64^- strongly inhibited induction of these genes (Fig. 2d, Supplementary Fig. 3c). Moreover, in an independent dataset and cellular system comparing Rasless MEFs (N- and HRAS-deficient MEFs^65^) reconstituted with near-endogenous level expression of either KRAS^WT^ or KRAS^G12D^, we also found an enrichment of interferon-related signatures to be upregulated along with *Mlkl* in KRAS-mutant cells (Supplementary Fig. 3d, e). Furthermore, using mouse PDAC cell lines derived from KC mice engineered to express a doxycycline‑inducible additional KRAS^G12D^ allele^66^ (dox-KRAS^G12D^ mPDAC) we could confirm a KRAS^G12D^ activity-dependent increase of *Mlkl*, *Zbp1* and *Oasl1* expression (Fig. 2e). Similarly, upon inducible expression of KRAS^G12D^ in normal human pancreatic duct epithelial (HPDE) cells and in the human PDAC cell line BxPC-3 we could confirm induction of *Dusp6* along with a significant induction of *Mlkl* while *Zbp1* was not expressed by these cells (Supplementary Fig. 3f, g). To gain insights into the expression pattern of necroptosis machinery components and ISGs in primary human PDAC on single cells level, we analysed a human scRNA-seq dataset including 24 patient and 11 normal pancreas samples^67^. Interestingly, the bona fide ISGs *Oas1* and *Isg20* were highly expressed in tumour but not normal pancreas (Fig. 2f–h). *Zbp1* and *Mlkl* as well as *Ripk3* expression was also enriched in tumour versus normal cells albeit with a heterogeneous distribution across patients and within single patients (Fig. 2i–k). While these data indicate that intra- and interpatient heterogeneity exists regarding ISG expression, they confirm a general upregulation of ISGs within PDAC.

To next test whether this concept was potentially more widely applicable to other cancers with Ras pathway activation, we computed an ISG score from 11075 patient samples across all cancer types present within the TCGA based upon a published set of 38 ISGs^68^ and correlated it with the respective Ras84 score in each cancer type. Notably, all cancer entities typically presenting with activating mutations in KRAS including PDAC, Lung adenocarcinoma (LUAD) and Colon adenocarcinoma (COAD) also scored high in ISG score (Fig. 2l). Interestingly, also other cancers with high Ras84 signature in the absence of Ras mutations scored high in the ISG score. Moreover, pan-cancer analysis separating high, intermediate and low ISG score cases revealed a strong correlation with the necroptosis molecular machinery as well as necroptosis-protective caspase 8 (Fig. 2m). Lastly, we computed a necroptosis score for all human cancers using combined expression of *Ripk3*/*Mlkl*/*Zbp1*. Plotting this score against the ISG score across human cancers revealed a strong correlation to exist between the two parameters (Fig. 2n). Notably, the strong association of ISG signature expression along with a high necroptosis pathway score also held true in cancers with low Ras84 score such as DLBCL (DLBC) and acute myeloid leukaemia (LAML). Likewise, representative DLBC cells were sensitive to experimental induction of necroptosis (Fig. 2o). Conversely, ISG-low Adrenocortical Carcinoma (ACC) cells exhibited necroptosis resistance (Fig. 2p). These findings suggest that in addition to the Ras pathway-driven increase in ISG signature, the ISG signature itself serves as an independent determinant of necroptosis sensitivity beyond RAS activation status. Compared with other oncogenic alterations, KRAS mutations showed a uniquely strong association with the necroptosis score, producing the highest values and an exceptionally significant correlation (*q* = 1.4 × 10^⁻⁹⁶^). In contrast, most other mutations were associated with only minimal, non-significant increases in necroptosis score. BRAF-mutant cancers were the only additional group reaching statistical significance; however, the association was markedly weaker (*q* = 0.01) and the phenotypic distribution substantially more heterogeneous than in KRAS-mutant tumours. Together, these data indicate that type I IFN pathway activation downstream of oncogenic KRAS may be responsible for upregulation of essential components of the necroptotic machinery.

### Expression of oncogenic KRAS induces STING-dependent type I IFNs

**Fig. 3:**
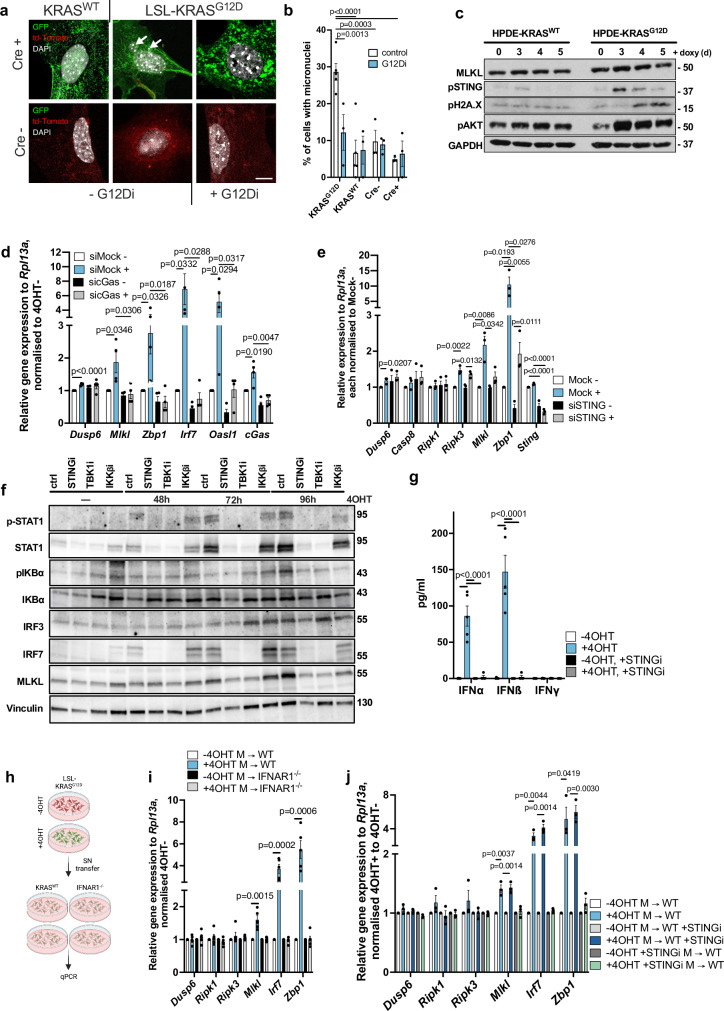
Expression of oncogenic KRAS induces interferon-stimulated genes (ISGs) in a STING-dependent manner. **a**, **b** LSL-KRAS^G12D^; mTmG (*n* = 3) and control LSL-mTmG (KRAS^WT^) MEFs (*n* = 3) were pretreated with G12D inhibitor [10 nM] for 24 h. Due to spontaneous Cre recombination, GFP-positive LSL-KRAS^G12D^; mTmG are KRAS^G12D^ with Cre expression and Tomato-positive are KRAS^WT^ without Cre expression. GFP and Tomato-positive control cells are KRAS^WT^ with or without Cre expression. **a** Representative confocal images; arrows indicate micronuclei, **b** percentage of micronuclei-containing cells. **c** Doxycycline inducible KRAS^WT^ or KRAS^G12D^ HPDE cells were treated with control or doxycycline [1 mg/ml] for indicated number of days. Representative Western Blots are shown. **d**, **e** LSL-KRAS^G12D^ MEFs were pretreated together with siMock (*n* = 4-with siGAS/3-with siSTING) or (**d**) siGAS (*n* = 4) or (**e**) siSTING-mediated (*n* = 3) knockdown for 48 h, afterwards medium replaced and cells treated +/− 4OHT [1 µM] and fresh siMock/siGAS/siSTING-mediated knockdown. Expression of the indicated genes was quantified by qPCR after 96 h incubation +/− 4OHT. **f** LSL-KRAS^G12D^ MEFs were treated with or without 4OHT [1 µM] for the indicated time. The following inhibitors or DMSO (ctr) were added together with 4OHT [1 µM]: STINGi [10 µM], TBK1i [5 µM], IKKβi [10 µM]. Representative Western Blots for the indicated proteins are shown. **g** LSL-KRAS^G12D^ MEFs were treated +/− 4OHT [1 µM] (*n* = 5) for 96 h to induce KRAS^G12D^ +/− STINGi [10 µM] (*n* = 4). Supernatants were subjected to ELISA quantification of the indicated proteins. **h** Scheme of experimental set up was created with Biorender. JS (2025) (https://BioRender.com/ffudhn3). **i** LSL-KRAS^G12D^ MEFs were treated +/− 4OHT [1 µM] for 36 h to induce KRAS^G12D^. After which media were replaced with 4OHT-free media and incubated for additional 60 h. Collected supernatants were applied to the recipient WT (*n* = 5) or *ifnar1*^*−/−*^(*n* = 5) MEFs for 60 h. mRNA expression of the indicated genes was quantified by qPCR. M – media. **j** As in **i** but with STINGi [10 µM] added to donor and/or recipient WT MEFs +/− STINGi [10 µM, 2 days prior] (*n* = 3). Each gene is normalised to its respective non-induced media control stimulation. Data are means +/− SEM. Two-way ANOVA (**b**, **e**, **g**) and multiple unpaired *t* test (**d**, **i**, **j**) were used. Source data are provided as a Source Data file.

A major activator of the type I IFN pathway is the cyclic GMP-AMP synthase (cGAS)/stimulator of interferon genes (STING) pathway^69^. STING is activated by cGAS binding to cytosolic DNA which can be released from stressed mitochondria (mitochondrial DNA; mitoDNA)^70^. Yet, effective depletion of mitochondrial DNA using ddC or IMT1B did not affect KRAS-induced ISG induction (Supplementary Fig. 4a, b) ruling out mitochondrial DNA as the source. Cytosolic DNA can also arise from micronuclei forming at the periphery of the nucleus^69,71^. Indeed, we observed oncogenic KRAS activity-dependent induction of micronuclei (Fig. 3a, b). Moreover, induction of oncogenic KRAS in HPDE and BxPC-3 human pancreatic cell lines, led to time-dependent phosphorylation of STING (Fig. 3c, Supplementary Fig. 4c). Notably, we also detected p-H2AX upon oncogenic KRAS expression, yet the signal increased only after phosphorylation of STING suggesting DNA damage to follow STING activation rather than precede it in this context (Fig. 3c and Supplementary Fig. 4c). Importantly, silencing of either *cGAS* or *STING* was sufficient to block ISG induction upon oncogenic KRAS expression (Fig. 3d, e, Supplementary Fig. 4d). Downstream of STING, TANK-binding kinase 1 (TBK1) or NFkB are known to be activated^69^. To determine which of these arms may enhance *Mlkl* expression upon KRAS induction we used small molecule inhibitors against either TBK1 or IKK-ß, a component of the IKK complex which activates NFkB. While a STING inhibitor (STINGi; C-178) or TBK1 inhibitor (TBK1i; GSK8612) also blocked phosphorylation of IkB- α -in line with its described upstream function in NFkB activation^72^ - only inhibition of STING or TBK1 was capable of blocking KRAS-induced MLKL induction along with IRF7 accumulation and STAT1 activation (Fig. 3f). While type I IFN release was not evident in WT MEFs expressing Cre recombinase upon 4OHT-treatment (Supplementary Fig. 4e), we detected significant levels of secreted IFN-α and -ß but not -γ upon induction of KRAS^G12D^ expression which was blunted upon incubation with a STINGi (Fig. 3g). Soluble type IFNs activate ISG expression downstream of type I IFN receptor 1 (IFNAR1) and 2 (IFNAR2). Therefore, we next tested whether supernatants collected from KRAS^G12D^- uninduced as compared to -induced cells might be sufficient to stimulate expression of the necroptosis machinery in recipient WT or *Ifnar1*^−/−^ cells lacking the LSL-KRAS^G12D^ locus. Indeed, supernatants from KRAS^G12D^-induced cells stimulated expression of *Mlkl* in WT but not in *Ifnar1*^−/−^ MEFs (Fig. 3h, i). Of note, *Dusp6* was, as expected, not induced in these cells given that they did not express oncogenic KRAS. Moreover, in another supernatant transfer experiment, recipient WT cells stimulated with conditioned media from KRAS^G12D^-induced cells in the presence of STINGi did not induce expression of necroptosis-associated ISGs (Fig. 3j) indicating the requirement for STING in supernatant producing but not receiving cells. Together, these data show that expression and activity of oncogenic KRAS induces type I IFNs in a cGAS/STING/TBK1-dependent manner resulting in enhanced expression of necroptosis-associated ISGs downstream of *ifnar1*.

### Necroptosis-associated ISGs are induced in an ISGF3-dependent manner

**Fig. 4:**
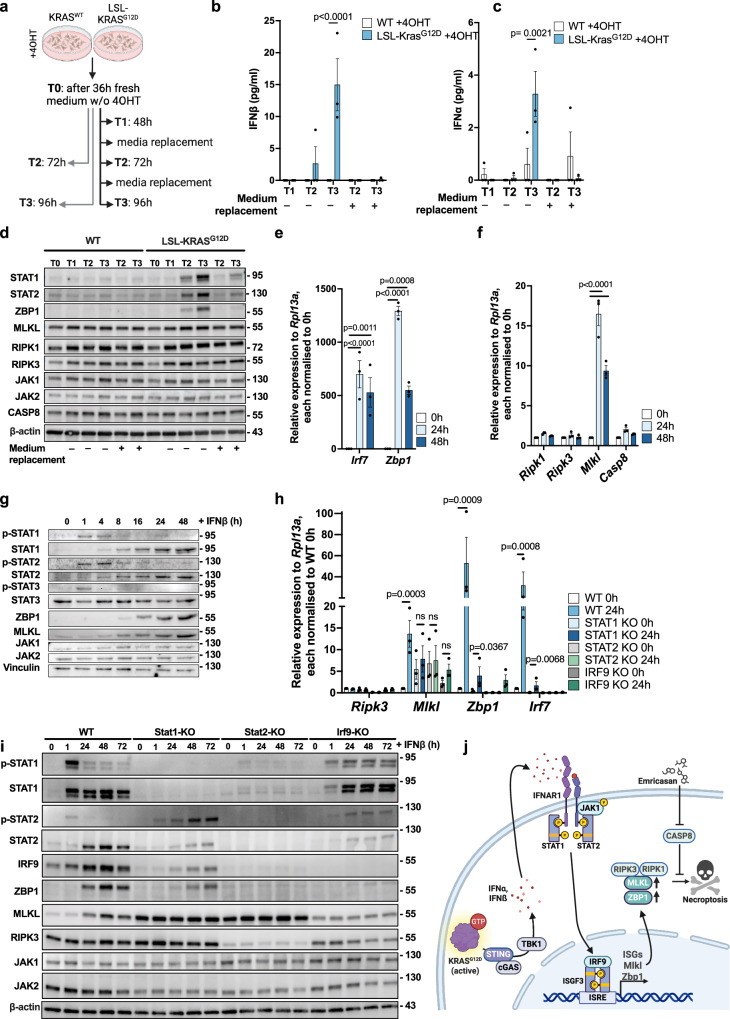
Necroptosis-associated interferon-stimulated genes (ISGs) are induced in a STAT1/2/IRF9-dependent manner. **a** Scheme of experimental set up was created with Biorender. JS (2025) (https://BioRender.com/ffudhn3). **b**, **c** WT (*n* = 3) or LSL-KRAS^G12D^ MEFs (*n* = 3) were treated with 4OHT [1 µM] for 36 h, media were replaced without adding 4OHT and incubated for the indicated times (T1; 48 h, T2;72 h, T3;96 h without or with media replacement every 12 hours). Concentrations of the indicated type I IFNs in media were quantified using ELISA. **d** Cells treated as in (**b**) were subjected to Western Blotting. **e**, **f** WT MEFs (*n* = 3) were treated with IFN-ß [100 ng/ml] for the indicated times. Expression of the indicated genes was quantified by qPCR. **g** Cells as in **e** were treated with IFN-ß [100 ng/ml] for the indicated times. Protein expression was analysed by Western Blotting. **h**, **i** WT, STAT1-, STAT2- and IRF9-deficient MEFs (*n* = 3 for each) were treated with IFN-ß [100 ng/ml] for the indicated times. **h** Expression of the indicated genes was quantified by qPCR. **i** Protein expression of indicated proteins were analysed by Western Blotting. **j** Schematic representation of the proposed mechanism was created with Biorender. JS (2025) (https://BioRender.com/ffudhn3). Relative expression of genes normalized to housekeeping gene *Rpl13a* and respective control. Data are means +/− SEM or representative images if not indicated otherwise. 2way ANOVA with Šidák correction (**b**, **c**), with Dunnett correction (**e**, **f**). Unpaired *t* test on log2-transformed data (**h**) and Fisher’s LSD test (**i**). Source data are provided as a Source Data file.

To next determine whether accumulation of type I IFNs in the medium is sufficient to induce autocrine induction of ISGs after induction of oncogenic KRAS, we devised an experiment in which WT or LSL-KRAS^G12D^ MEFs were treated with 4OHT for 36 h after which media were exchanged without re-adding 4OHT (T0). In one group, we then exchanged the media again every 12 h to prevent accumulation of type I IFNs, while we left the other group’s media unchanged after T0. Both group’s supernatants were collected and analysed after 48 h (T1; 12 h after T0), after 72 h (T2; 36 h after T0) and after 96 h (T3; 60 h after T0) (Fig. 4a). Indeed, supernatants from LSL-KRAS^G12D^ MEFs without media replacement showed an accumulation of soluble type I IFNs over time, while neither WT supernatants nor LSL-KRAS^G12D^ supernatants with media exchange replacement every 12 h contained measurable amounts of type I IFNs (Fig. 4b, c). Importantly, only LSL-KRAS^G12D^ MEFs without media replacement after T0 showed a clear induction of ISGs such as *Zbp1*, while this was blunted in the media replacement group (Fig. 4d). Stimulation of WT MEFs with recombinant IFN-ß phenocopied the effect of oncogenic KRAS induction in inducing ISGs, including *Zbp1*, *Mlkl* (Fig. 4e–g). Moreover, as with induced LSL-KRAS^G12D^ MEFs, we observed an induction of STAT1 and −2 activation upon IFN-ß stimulation, followed by an upregulation of ZBP1 and MLKL (Fig. 4g). Downstream of oncogenic KRAS-induced type I IFNs we found JAK1 but not JAK2 to be involved in the induction of necroptosis associated ISGs (Supplementary Fig. 5a). To test whether this effect would be fully phenocopied by recombinant IFN-ß in WT MEFs we silenced *Jak1*, *Jak2* or both using small interfering RNA (siRNA). Again, *Jak1* but not *Jak2* silencing was sufficient to reduce IFN-ß-induced ISGs including *Irf7*, *Zbp1* and *Mlkl* (Supplementary Fig. 5b–d). These data suggested that type I IFN-induced necroptosis-associated ISGs might be dependent upon STAT1/STAT2/IRF9-mediated transcription. To test this hypothesis, we made use of control, STAT1, STAT2 and IRF9-deficient MEFs^73^. Importantly, IFN-ß-dependent induction of ISGs including ZBP1 and MLKL was blunted in STAT1, − 2 and IRF9-deficient cells (Fig. 4h, i). Given that STAT1 and IRF9 expression is dependent on STAT2, STAT2-deficient cells did not express STAT1 or IRF9 either (Fig. 4i). Moreover, IFN-ß-induced STAT2 and IRF9 were strongly impaired in STAT1-deficient cells. While basal levels of ZBP1 were also entirely dependent upon STAT2 expression, MLKL basal expression was elevated in STAT2-deficient cells but its specific induction upon IFN-ß stimulation was entirely blunted suggesting different regulatory mechanisms for tonic expression levels of MLKL as compared to induced levels (Fig. 4h, i). Of note, STAT1/2 and IRF9 form a transcription factor complex termed interferon-stimulated gene factor 3 (ISGF3). Based on these data, we propose a model in which oncogenic KRAS induces soluble type I IFNs in a STING-TBK1-dependent manner causing the upregulation of ISGs including ZBP1 and MLKL via ISGF3-mediated transcription (Fig. 4j).

### Oncogenic KRAS induces necroptotic priming in a STING-dependent manner

**Fig. 5:**
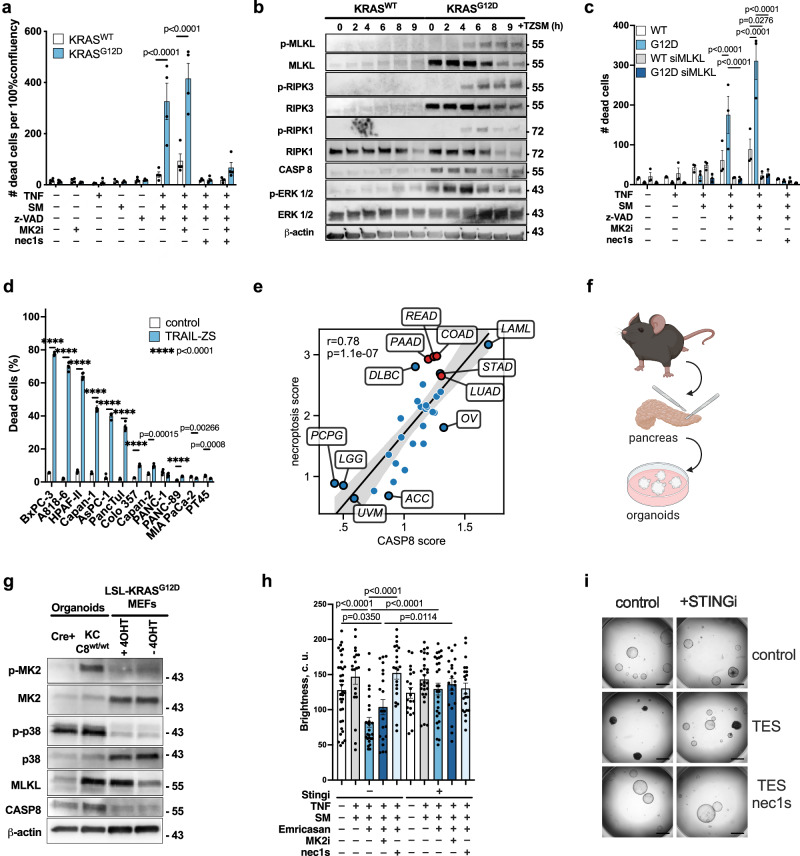
Oncogenic KRAS expression sensitises to necroptosis through STING activation. **a** KRAS G12D and KRAS WT MEFs (Rasless MEFs constitutively reconstituted) (*n* = 4 each) were treated with (SM, birinapant [1 μM], z-VAD [20 μM], MK2i [20 μM], nec1s [10 μM], TNF [50 ng/ml] for 24 h. Cell death was analysed by DRAQ7 fluorescence with normalization to cell confluence using IncuCyte. **b** Cells treated as in a for the indicated times. Representative Western blots are shown. **c** Cells as in a were pretreated together with siMock or siMLKL-mediated knockdown for 48 h and treated as indicated (SM, birinapant [1 μM], z-VAD [20 μM], MK2i [20 μM], nec1s [10 μM], TNF [50 ng/ml] for 24 h (*n* = 3). Dead cells (eFluor660+ cells) were quantified by flow cytometry. **d** Human PDAC cell lines (*n* = 4 each) were treated with TRAIL (TRAIL) [100 ng/ml], z-VAD (Z) [20 μM] and the SMAC mimetic birinapant (S) [1 μM]. Percentage of dead cells from cells treated was analysed using the NYONE fluorescence assay. **e** A necroptosis score was computed based upon combined *Ripk3*, *Mlkl* and *Zbp1* expression and correlated to the median caspase 8 expression across all human TCGA datasets. Red dots mark cancers with frequent KRAS mutations. **f** Schematic representation of the isolation of murine pancreatic ducts from KC mice for organoid generation was created with Biorender. JS (2025) (https://BioRender.com/ffudhn3). **g** Organoids were generated from 5-month-old C-C8^wt/wt^ and KC-mice, LSL-KRAS^G12D^ MEFs control or treated with 4OHT for 96 h. Representative Western Blots for the indicated proteins are shown. **h**, **i** Organoids were isolated from 5-month-old KC-mice and seeded +/− the indicated treatments for 24 h: STINGi [10 µM], TNF [500 ng/ml], SM [1 µM], emricasan [2.5 µM], MK2i [10 µM] and RIPK1i [nec1s, 10 µM]. **h** Organoid viability was quantified using organoid brightness as a proxy and the BZ-X800E microscope (Keyence) and BZ-H4M/Measurement Application Software (Keyence). *n* = 35, 20, 26, 21, 22, 20, 27, 28, 21, 22 independent organoids from 2 repeats (left to right). **i** Representative images are shown. Data are means +/− SEM. Two-way ANOVA (**a**, **c**), multiple unpaired *t* test (**d**) and one-way ANOVA (**h**) were used. Source data are provided as a Source Data file.

Next, we tested whether the type I IFN-mediated upregulation of necroptosis effectors, i.e. necroptotic priming, was sufficient to selectively sensitise cells expressing oncogenic KRAS to necroptosis. Strikingly, constitutive expression of KRAS^G12D^ was sufficient to selectively sensitise to necroptotic cell death commonly induced by TNF in combination with the pan-caspase inhibitor z-VAD and SMAC mimetics (Fig. 5a). Co-treatment with the RIPK1 inhibitor nec1s reverted cell death induction indicative of RIPK1-dependent necroptosis. Oncogenic KRAS upregulates the stress response p38/MK2 kinase pathway^74^ and MK2 was shown to place an inhibitory role on the phosphorylation of RIPK1 limiting thereby necroptosis^75^. Indeed, MK2 inhibitor (MK2i) addition further sensitised cells expressing oncogenic KRAS to RIPK1-dependent necroptosis. Furthermore, replacing TNF as the stimulating TNF superfamily ligand by other TNF superfamily death ligands such as CD95L or TRAIL equally sensitised KRAS expressing cells to necroptosis (Supplementary Fig. 6a, b). Inducible expression of KRAS^G12D^ from its endogenous locus in LSL-KRAS^G12D^ MEFs also sensitised to necroptosis (Supplementary Fig. 6c) and MRTX1133 treatment of KRAS-mutated mouse PDAC cells partially reverted necroptosis sensitivity of these cells (Supplementary Fig. 6d). In addition, necroptosis induction using a combination of TNF/sSMAC mimetics/caspase inhibitor and MK2i (TZSM) in KRAS^G12D^-expressing MEFs led to an earlier detection of phosphorylated MLKL and RIPK3 along with an earlier induction of necroptotic cell death (Fig. 5b, Supplementary Fig. 6e, f). Notably, in support of type I IFNs driving sensitisation, a 48h-pretreatment with recombinant IFN-ß was also sufficient to sensitise WT MEFs to necroptosis (Supplementary Fig. 6g). Importantly, effective siRNA-mediated silencing of *Mlkl* was sufficient to revert KRAS-endowed necroptosis sensitisation (Fig. 5c, Supplementary Fig. 6h). In addition, induction of oncogenic KRAS expression with concomitant induction of caspase 8 knockout (LSL-KRAS^G12D^; C8^fl/fl^ MEFs) led to spontaneous cell death that was further enhanced by TNF/SMAC mimetic and reverted by Nec-1s indicating the crucial role for caspase 8 in antagonising necroptosis in oncogenic KRAS-expressing cells (Supplementary Fig. 6i).

Unlike MEFs, human cancer cell lines frequently do not express RIPK3 despite detectable RIPK3 expression in associated primary patient tissue^76^ (Fig. 2k). We also observed variable expression of RIPK1, RIPK3 and MLKL in a panel of human PDAC cell lines (Supplementary Fig. 6j). Nevertheless, about half of the human PDAC cell lines tested known to highly express TRAIL-receptor 2^13^ showed some level of sensitivity to necroptosis induced by TRAIL in combination with the caspase inhibitor zVAD and sSMAC mimetic birinapant (TRAIL-ZS) (Fig. 5d). Of note, BxPC-3, a KRAS WT cell line was also highly sensitive to necroptosis possibly due to the fact that BxPC-3 cells harbour oncogenic deletions within BRAF^77^ leading to its constitutive activation and hence, equally show RAS-pathway activation. Indeed, we computed the Ras84 signature and all cell lines, including BxPC-3 scored high at about 0.9 (Supplementary Fig. 6k) which was comparable to primary human PDAC (Fig. 1a). In addition, we observed that gene expression levels of MK2 (MAPKAPK2) were overall higher while RIPK3 expression slightly lower in non-responders (cell lines which showed less than 20% of cell death) and vice versa cFLIP expression was elevated in responders (cell lines which showed more than 30% of cell death). While such expression patterns could provide a hint regarding necroptosis sensitivity or resistance in these cell lines, it is more likely that multiple factors play a role their ability to respond (Supplementary Fig. 6l). To assess caspase targetability and necroptotic priming of human PDAC but circumnavigate the problem of necroptosis-associated ISG heterogeneity in human PDAC cell lines, we cross-correlated the necroptotic priming score with caspase 8 expression across TCGA datasets. Strikingly, all cancers with frequent KRAS mutations (red) grouped together with the cancers with highest necroptotic priming score (Fig. 5e).

To test whether the potential synthetic lethality of necroptotic priming can be exploited therapeutically, we isolated murine ducts from KC-mice and 3D-cultured them to obtain organoids (Fig. 5f). In line with expression of oncogenic KRAS inducing p38/MK2 pathway activation^74^, we detected higher p-MK2 levels in KC organoids compared with normal ductal organoids and MEFs, and increased p-p38 levels relative to MEFs (Fig. 5g). Therefore, we opted to induce necroptosis using TNF/clinically approved caspase inhibitor emricasan/SMAC mimetics (TES) and include MK2i. Indeed, both combinations rapidly killed KC-organoids the death of which was reverted by alleviating priming through pretreatment with STINGi (Fig. 5h, i). Taken together, our data further indicate that STING-induced necroptotic priming is a feature sensitising cells expressing oncogenic KRAS to necroptotic cell death.

### Pharmacological induction of necroptosis is an effective therapeutic strategy in PDAC

**Fig. 6:**
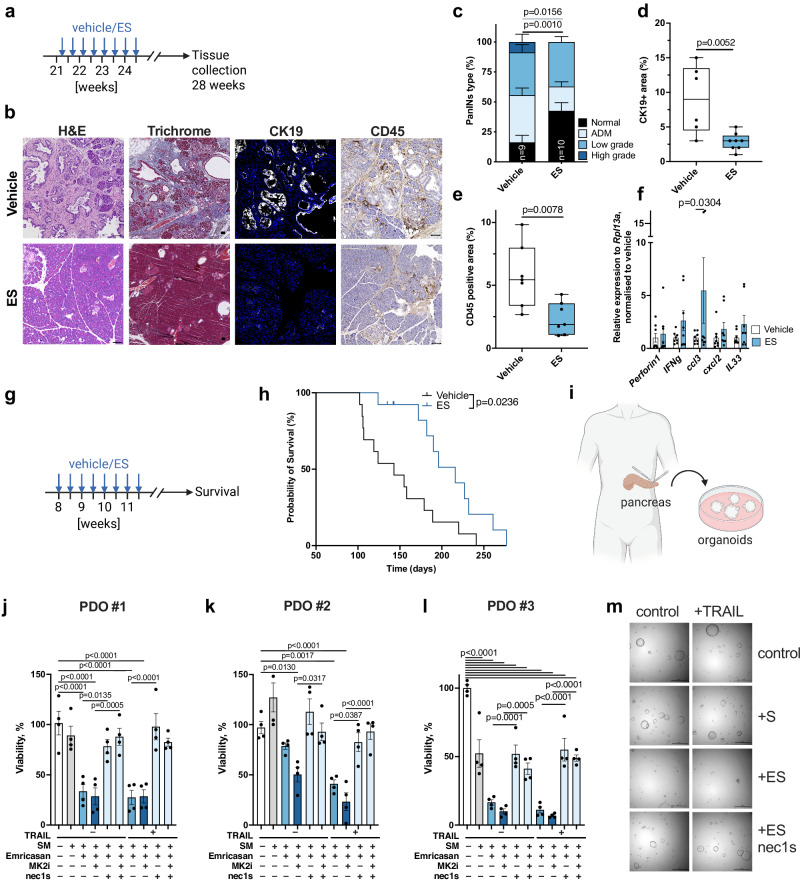
Pharmacological induction of necroptosis is an effective therapeutic strategy in PDAC. **a** Scheme of KC-mice treatment schedule. **b** 5-month-old KC-mice were treated i. p. with vehicle (PBS with 40% PEG-4000, 0.4% DMSO) (*n* = 9) or Emricasan (E) [2.5 mg/kg]/Birinapant (SMAC mimetic; S) [5 mg/kg] (*n* = 10), twice a week for 4 consecutive weeks. Four weeks after the last treatment the mice were sacrificed, pancreata excised and tissues analysed by the indicated stains. Representative images are shown. Size bars represent 100 µm. **c** In pancreata from treated mice as in (**a**), % of ducts visible per section were graded and quantified by a pathological blinded to the group allocation. **d** Vehicle (*n* = 6) and ES (*n* = 8)-treated sections were stained for CK19 using immunofluorescence and CK19^+^ area was quantified as before. Boxplot centre line, mean; box limits, upper and lower quartile; whiskers min. to max. **e** Vehicle (*n* = 7) and ES (*n* = 7)-treated sections were stained for CD45 using immunohistochemistry and CD45^+^ area was quantified with QuPath. Boxplot as in (**d**). **f** mRNA expression of the indicated genes within bulk pancreatic mRNA extracts from mice treated as in (**d**) was quantified using qPCR in vehicle (*n* = 8) and ES (*n* = 8)-treated pancreata. **g** Scheme of KPC mice treatment schedule. **h** 8-week-old KPC mice were treated with vehicle (*n* = 13) or the combination of Emricasan [2.5 mg/kg]/Birinapant [5 mg/kg] (*n* = 13). Mice were kept for Kaplan–Meier survival studies until experimental endpoint was met. **i** Scheme of human PDAC organoid isolation from pancreatic cancer patients was created with Biorender. JS (2025) (https://BioRender.com/ffudhn3). **j**–**m** Three distinct human PDAC patient-derived organoids (PDOs) (*n* = 4 for each PDO line) were treated +/− TRAIL [500 ng/ml], SMAC mimetic (SM, birinapant) [1 µM], emricasan [5 µM], MK2i [10 µM] and nec1s [10 µM] for 72 h. **l** Organoid viability was determined by cell Titer Glo. **m** Representative images are shown. Data are means +/− SEM. Two-way ANOVA (**c**), unpaired *t* test (**d**, **e**), two-way ANOVA with Šídák multiple comparison (**f**), Gehan-Breslow-Wilcoxon test (**h**) and Ordinary one-way ANOVA with Šídák multiple comparison (**j**–**l**) were used. Source data are provided as a Source Data file.

To next test efficacy of therapeutic induction of necroptosis in a KRAS-driven cancer model in vivo, we treated 21-week-old KC mice for 4 weeks with either vehicle or combined caspase inhibition with a SMAC mimetic (emricasan/birinapant, ES) (Fig. 6a). Strikingly, this combination showed a very strong anti-tumour effect achieving a restoration of almost 50% of normal pancreatic ducts and a strongly reduced presence of CK19^+^ area as well as less CD45^+^-cells (Fig. 6b–e). While CD45^+^ cells relative to residual CK19^+^ area or TUNEL^+^ cells were not significantly different between both groups at this time point (Supplementary Fig. 7a–c). Importantly, while we could detect cleaved caspase 3 and TUNEL+ positive cells in vehicle-treated KC-mice, we observed cases of TUNEL-positive cells which had adjacent cleaved caspase 3- negative sections in emricasan/birinapant-treated KC-mice, suggesting the presence of regulated necrosis (Supplementary Fig. 7d). Moreover, KC-mice receiving emricasan/birinapant also presented with elevated pancreatic levels of CCL3 and other M1-type inflammatory chemokines and cytokines observed in the context of genetic induction of necroptosis (Fig. 6f). While the KC-mouse model can progress to develop PDAC, this takes a long time and does not recapitulate other features of aggressive PDAC such as ascites accumulation and metastasis. Therefore, we next tested therapeutic efficacy of targeting necroptotic priming in mice expressing LSL-KRAS^G12D^ and LSL-Trp53^R172H^ in the pancreas (KPC mice) which gives rise to highly aggressive metastasizing PDAC^78^. Strikingly, treatment with emricasan/birinapant for only 4 weeks was sufficient to drastically prolong overall survival of KPC mice (Fig. 6g, h). Upon necropsy at experimental endpoint, 4 out of 13 mice showed liver and lung macrometastasis in the vehicle group, whereas only 2 of 13 mice in the emricasan/birinapant group exhibited metastasis, despite a significantly extended survival time. Since necroptosis can be initiated through both RIPK1-dependent and ZBP1-dependent mechanisms, yet, ZBP1 expression is tightly regulated, we assessed its expression throughout PanIN-to-PDAC progression in mice. Notably, *Zbp1* expression was only upregulated at later stages in KPC tumours (Supplementary Fig. 7e). These data suggest that while it is possible that ZBP1 participates in necroptosis induction in PDAC, its expression seems counter selected in earlier PanIN lesions. Given the promising results obtained in mouse models of PDAC, we next opted to therapeutically exploit necroptotic priming in PDAC patient-derived organoids (PDOs). PDOs were obtained from surgical specimen of PDAC patients (Fig. 6i) and cultured as described^79^. Strikingly, induction of necroptosis using emricasan/birinapant with or without additional TRAIL and with or without MK2i led to a drastic anti-tumour effect in all three human PDOs (Fig. 6j–m). Of note, necroptosis induction in PDO #1 and #3 did not require addition of exogenous TRAIL while PDO #2 showed additional benefit. Importantly, the loss of viability observed was reverted by co-treatment with nec1s indicating the induction of necroptotic cell death. Collectively, these data suggest that pharmacological induction of necroptosis might be an effective therapeutic strategy to treat human PDAC patients.

## Discussion

In this work, we found that expression of the PDAC hallmark oncogene KRAS drives a cytosolic DNA/cGAS/STING-dependent type I interferon response leading to “necroptotic priming”. This priming state allowed for pharmacological intervention using necroptosis induction. Strikingly, this resulted in drastically prolonged survival of mice with PDAC and effectively reduced viability of human PDAC patient organoids. These data provide grounds for a much-needed effective therapeutic approach for the treatment of PDAC while uncovering a mechanism linking oncogenic KRAS to type I IFN signalling.

In the context of inflammation, the core necroptosis pathway machinery has been described to be regulated as part of a transcriptional IFN response^60^ including ZBP1^61,62^ and MLKL^63^. While the KRAS-induced soluble type I IFN response may overall serve pro-survival responses and resistance to DNA damage^80^ we find that it comes at the collateral cost of high necroptotic priming. Notably, KRAS-mutated cells nevertheless survive due to elevated expression caspase 8 acting as a block towards necroptosis. This principle follows a similar logic as the concept of “apoptotic priming” in which BH3 domain profiling identified that certain lymphomas are more primed than others towards intrinsic apoptosis but kept alive by elevated BCL-2 overexpression^81^. Importantly, while KRAS-mutated cells are known to be resistant to apoptosis on multiple levels, we find that the opposite holds true for the necroptosis pathway.

In addition, we find that KRAS-induced type I IFN production is STING/TBK1-dependent. Seemingly in contrast to our findings, KRAS inhibition in vivo has been shown to enhance IFN-related programs^82,83^. However, these studies were conducted within the framework of KRAS oncogene addiction. This raises the possibility that here cell death was a primary consequence of KRAS inhibition, which subsequently might have triggered secondary activation of immune cells—the likely predominant source of the observed cytokine and chemokine production. Moreover, direct KRAS-dependent suppression of type I IFN signalling was reported^84^. One notable experimental difference between the cited study and ours is that KRAS-mutant cells in Muthalagu et al. expressed high levels of MYC, whereas MYC expression was relatively low in our cells, as determined by RNA-seq. Notably, MYC expression itself can mediate repression of ISG expression^84^ and co-operate with KRAS signalling, which may explain why KRAS signalling was inhibitory in their system but not in ours. Consistent with our experimental data in cells, independent patient single-cell datasets showed higher ISG expression in human PDAC compared with normal pancreas (Fig. 2f–k). Moreover, analysis of TCGA datasets revealed that ISG levels strongly and positively correlate with the Ras84 score, and that KRAS-mutant cancers, including PDAC, lung adenocarcinoma (LUAD), and colon adenocarcinoma (COAD), rank among those with the highest ISG expression (Fig. 2l). Collectively, these observations indicate that while the influence of oncogenic KRAS on type I IFN signalling may vary across in vitro experimental systems and may depend on co-mutations, our findings align well with patterns observed in patient-derived data.

Aside from its function as essential necroptosis executioner, in the context of low RIPK3 expression MLKL was recently shown to promote tumour-supportive inflammation in liver cancer through the formation of a sublethal necrosome^85^. Interestingly, we also observed slightly decreased incidence of metastasis in KC-MLKL^−/−^ mice which might be caused by this effect, yet upon co-deletion of caspase 8 (KC-C8^fl/fl^; MLKL^−/−^) this effect was reversed (Supplementary Fig. 1j, k). These data argue that in the presence of high levels of caspase 8, MLKL might favour a cancer-promoting function yet, upon its deletion this shifts the balance towards cell death induction and elimination of PanINs. Of note, cancer cell lines were shown to migrate in response to conditioned media coming from necroptotic cells supporting a potential pro-migratory function of necroptosis in certain contexts^86^. While our data show that genetic triggering of necroptosis through caspase 8 deletion restricts PanINs, MLKL-deficiency alone did not significantly affect PanIN development in the presence of caspase 8 (Fig. 1e, f). In line with this, kinase-dead RIPK1 knock-in blocking necroptotic cell death- did not significantly affect KRAS-driven PDAC development in mice^87^. These data suggest that either spontaneous necroptosis does not significantly contribute to the disease phenotype due to elevated caspase 8 expression or that prior constitutive selection via necroptosis causes elevated caspase 8 expression to counteract selection. Moreover, caspase 8 and MLKL co-deletion led to increased metastasis, hence, arguing for some degree of spontaneous tumour necroptosis to shape its evolution. With this in mind, we would expect kinase-dead RIPK1 knock-in to also rescue the PanIN exacerbation phenotype observed in KC-C8^fl/fl^ mice. While these observations might give a hint, they will warrant further investigations into the precise role of spontaneous necroptosis in shaping PDAC metastasis.

In addition to the here discovered causal role of oncogenic KRAS itself in driving an IFN response, caspase 8 or FADD deletion were shown to induce *Zbp1*- expression and -dependent necroptosis through a cGAS/STING-mediated pathway^88^. Along similar lines, absence of caspase 8 may additionally unleash RIPK1/TBK1-dependent induction of an IFN response^89^ which may contribute towards the oncogenic KRAS-induced IFN response we observed here upon caspase 8 deletion in vivo. Interestingly, therapeutic induction of necroptosis in acute myeloid leukaemia (AML), one of the most necroptotically primed cancers we find (LAML, Fig. 2n), was shown to be highly effective^47^. These data suggest that targeting necroptotic priming might represent a therapeutic treatment strategy which is potentially efficacious beyond PDAC in cancers with high ISG scores.

Mechanistically, we find that KRAS-induced cells secrete type I IFNs which are responsible for the induction of further downstream ISGs. This opens the intriguing possibility that aside from an autocrine effect within KRAS-mutated cells, their secretome may elicit a type I IFN response also in bystander cells including cancer-associated fibroblasts (CAFs). Recently, inflammatory CAFs (iCAFs) have been described which present with activation of IFN-related pathways^54,90,91^. Of note, we also find very high expression of all necroptosis pathway components tested including the strong IFN pathway target gene *Zbp1* in iCAFs (Supplementary Fig. 7g). Therefore, the strong therapeutic effect observed with induction of necroptosis using combined emricasan/birinapant in KC and KPC mice may in part also derive from targeting iCAFs along with cancer cells. In addition, this also suggests that even in cases where cancer cells may have disabled the necroptosis pathway -as we have observed in several cases of human PDAC cell lines- therapeutic induction of necroptosis may still elicit a therapeutic response by targeting the tumour microenvironment (TME). While we observed that the genetic induction of necroptosis led to increased neutrophil recruitment, the TME, in particular adaptive immune cells such as T-cells did not show overt signs of activation making it unlikely that they play a role in the anti-tumour effect observed. In addition to PDAC cells expressing oncogenic KRAS, other cells within the PDAC TME have been shown to express type I IFNs^92^, which would similarly prime PDAC tumours for necroptotic cell death. As a cautionary note, necroptotic priming was reverted upon treatment with a KRAS^G12D^ inhibitor (MRTX1133) which suggests that therapeutic necroptosis induction would likely not be effective in the presence of such inhibitors. Nevertheless, recent clinical evidence demonstrates that acquired resistance to the KRAS^G12C^-selective inhibitor adagrasib mechanistically involved alternative activating alterations within the KRAS effector pathway^93^ which might similarly lead to necroptotic priming as observed in BRAF-mutated BxPC-3 cells. Importantly, targeting necroptotic priming was efficacious in three human PDOs. Interestingly, for some of them treatment with emricasan/birinapant was sufficient to trigger loss of viability which was reverted by nec1s co-treatment. These data suggest that these PDOs likely belong to a cell type described to activate an autocrine loop of TNF production upon SMAC mimetic treatment which kills them without the need to add exogenous TNF superfamily ligands^94,95^.

Over recent years, extensive molecular profiling efforts have defined several distinct molecular subtypes of PDAC^4–7^. Interestingly, a recent study identified an IFN signature as characteristic feature within a molecular PDAC subtype deriving from ductal cells and defined by hypomethylation of repetitive elements^96^. Moreover, this subtype largely overlapped with the prior described basal-like subtype of PDAC. Of note, the GSEA transcriptional response pattern we observed upon KRAS induction in MEFs strongly overlapped with the GSEA pattern observed within the basal-like PDAC subtype^4–7,37^. Based upon the observation that we found necroptotic priming to be caused by an oncogenic KRAS-induced type I IFN response, we propose that PDAC patients with the high IFN signature subtype comprising the basal-like subtype might benefit from therapeutic caspase inhibition combined with IAP inhibition stabilising RIPK1-driven necroptosis in the presence of TNF superfamily ligands^97^. Beyond Ras pathway activation, we also found that high ISG scores correlated with necroptotic priming in human TCGA datasets, hence it is tempting to speculate whether a high ISG score might be considered as biomarker group to predict necroptosis vulnerability of several additional aggressive human cancer entities beyond PDAC. Taken together, this study describes the principle of oncogenic KRAS-induced type I IFN signalling and simultaneously reveals that pharmacological necroptosis inducers might be used for human PDAC treatment.

## Methods

### Ethics approval

This study was conducted in compliance with all relevant ethical regulations. All animal experiments were approved by local government authorities (Landesamt für Verbraucherschutz und Ernährung, Nordrhein-Westfalen, LAVE, Germany, license numbers: 2017.A433; 2017.A477; 2022.A364) and were conducted in compliance with European, national and institutional guidelines on animal welfare at the University of Cologne, Germany. All people involved in animal experiments received prior training and have passed the additionally required personal licensing course (FELASA B). Mice were closely monitored and sacrificed at the indicated experimental endpoint (time) or later at humane endpoint when reaching a score 10 (moderate burden) within a scale of 20 (severe burden) to minimise animal suffering as approved by local authorities. The maximum burden was not exceeded in any experiment. The primary human PDO models were established and analysed in accordance with the Declaration of Helsinki; were approved by the local ethical committee of Technical University Munich (TUM), Klinikum rechts der Isar and LMU, Klinikum der Universität München (projects 207/15, 1946/07, 330/19S, 80/17S, 5542/12, and 17-648); and written informed consent from the patients for research was obtained prior to the investigation. Publicly available TCGA and other cited RNA-sequencing datasets were used; prior ethics approval has been obtained for these studies, and no additional approval is required.

### Antibodies

The following antibodies for Western Blots were used: AKT (Cell Signaling, 4691 1:1000), Caspase 8 (AdipoGen, AG-20B-0057-C050 1:1000), Caspase 8 (Enzo Life Sciences, ALX-804-447-C100 1:1000), ERK (Cell Signaling, 9102 1:1000), GAPDH (Cell Signaling, 97166 1:2000), IKBα (Santa Cruz, sc-1643 1:1000), IRF3 (Abcam, ab68481 1:1000), IRF7 (Cell Signaling, 72073 1:1000), IRF9 (Cell Signaling, 28845 1:1000), JAK1 (Cell Signaling, 3344 1:1000), JAK2 (Cell Signaling, 3230 1:1000), MK2 (Cell Signaling, 3042 1:1000), MLKL (Cell Signaling, 14993 1:1000), MLKL (Millipore, MABC604 1:1000), p-AKT (Ser473) (Cell Signaling, 4060 1:1000), p-ERK (Thr202/Tyr204) (Cell Signaling, 4307 1:1000), p-IKBα (Cell Signaling, 9246 1:1000), p-MK2 (Thr334) (Cell Signaling, 3007 1:1000), p-MLKL (Ser345) (Cell Signaling, 37333 1:1000), p-MLKL (Ser358) (Cell Signaling, 91689 1:1000), p-p38 (Cell Signaling, 4511 1:1000), p-RIPK1 (Ser166) (Cell Signaling, 65746 1:1000), p-RIPK3 (Thr231/Ser232) (Cell Signaling, 91702 1:1000), p-STAT1 (Tyr701) (Cell Signaling, 9167 1:500), p-STAT2 (Tyr689) (Sigma-Aldrich, 07-224 1:500), p-STAT3 (Tyr705) (Cell Signaling, 9145 1:500), p38 (Cell Signaling, 9212 1:1000), RIPK1 (Cell Signaling, 3493 1:1000), RIPK3 (Cell Signaling, 15828 1:1000), RIPK3 (Enzo Life Sciences, ADI-905-242 1:1000), ß-Actin (Sigma, A1978, 1:10,000), STAT1 (Cell Signaling, 14995 1:1000), STAT2 (Sigma-Aldrich, 07-140 1:1000), STAT3 (Cell Signaling, 9139 1:1000), STING (Cell Signaling, 13647 1:1000), pSTING (Cell Signaling, 19781 1:1000), Vinculin (Cell Signaling, 13901 1:2000), ZBP1 (Adipogen, A42342106 1:1000)); HRP-conjugated secondary antibodies: goat-anti-mouse-HRP (Linaris GmBH, 20400, 1:10,000), goat-anti-rabbit-HRP (Linaris GmBH, 20402, 1:10,000), goat-anti-rat-HRP (Sigma, A9037, 1:10,000). The following antibodies were used for tissue stainings: Cytokeratin 19 (1:100, AHP1846, Bio rad antibodies (discontinued) and TROMA-III from Developmental Studies Hybridoma Bank Iowa University), CD45 (1:100, 14-0451-82, Invitrogen), Caspase 8 (1:200, ALX-804-447-C100, Enzo), cleaved caspase 3 (1:500, 9661, Cell Signaling), p-STAT1 (1:50, 9167, Cell Signaling). The following antibodies were used for micronuclei microscopy: DAPI (D1306, InvitrogenTM), Anti-DNA Antibody (CBL186, Sigma), ProLong Gold antifade (P36934, InvitrogenTM). Fluorescence-activated cell sorting (FACS) antibodies**:** Fc block (CD16/32, clone 93, 101301, biolegend, 1:50), CD45-BV421 (30.F11, 1:1000, 103133, biolegend), CD45-FITC (30.F11, 1:1000, 103107, biolegend), NK1.1- BV421 (PK136, 1:1000, 156537, biolegend), CD4-V450 (RM4-5, 1:1000, 560470, BD Horizon), CD11b-PE (M1/70, 1:1000, 12-0112-81, ebioscience), CD8a-PE (53-6.7, 1:1000, 100707, biolegend), CD19- PE (1D3, 1:1000, 12-0193-82, ebioscience), CD11c-BV421 (N418, 1:1000, 117329, biolegend), Ly-6G/Ly-6C-FITC (RB6-BC5, 1:1000, 108435, ebioscience), Gr1-BV-711 (RB6-8C5, 1:1000, 108443, biolegend), CD14-FITC (Sa14-2, 1:1000, 123307, biolegend), CCR3-FITC (J073E5, 1:1000, 144509, biolegend), CD206-BV421 (MMR, C068C2, 1:50, 141717, biolegend), Rat IgG2a, κ-isotype Ctrl -BV-421 (RTK-2758, 1:50, 400501, biolegend).

### Reagents

Birinapant (S/SM, S7015, sellekchem), Emricasan (HY-10396, Hölzel), zVAD (BML-P416-0001, ENZO), Ripk1i (nec1s, ab221984, Abcam), Ripk3i (GSK872, 530389, Millipore), PF-3644022 hydrate (PZ0188, Sigma Aldrich), G12Di (MRTX1133, HY-134813, sellekchem), TBK1i (GSK8612, S8872, sellekchem), IKKßi (BI 605906, Boehringer Ingelheim Opnme), IFNß1 (581302, biolegend), TRAIL (1121-TL-010/CF, R&D systems), Fas Ligand (6128-SA-025/CF, R&D systems), PD184352 (PZ0181, Sigma Aldrich), MK2206 (S1078, Sellekchem), Ruxolitinib (S1378, Selleckchem), STINGi (C-178, S6667, Selleckchem), Palbociclib (S1116, Sellekchem), IMT1B (HY-137067, MedChemExpress), 2′,3′-Didesoxycytidin / Zalcitabine / ddC (HY-17392, MedChemExpress), DRAQ7 (424001, Biolegend), Dharmafect I (T-2005-01, Dharmacon), Puromycin (P8833, Sigma), Doxycycline hydrochloride (J67043, Alfa Aesar), 4-hydroxy-tamoxifen (4OHT) (T5648, Sigma), HBS (H3162, Sigma Aldrich), propidium iodide (P4170, Sigma), AnnexinV (29005, Biotium), Matrigel (Growth Factor Reduced, Phenol Red Free; 356231, Corning), PancreaCult™ Organoid Growth Medium (06040, Stemcell). Knockdowns were performed using SMARTpool ON-TARGETplus: siJak1 (L-040117-00-0005, Horizon), siJak2 (L-040118-00-0005, Horizon), siMlkl (L-061420-00-0005, Horizon), siSTING (L-055528-00-0005, Horizon), siGAS (L-055608-01-0005, Horizon). Collagenase II (17101015, Thermo Fisher Scientific), RBC lysis buffer (A1049201, Thermo Fisher Scientific), TrypLE (12604039, Thermo Fisher Scientific), D-glucose (G8270, Sigma-Aldrich), ITS Premix (354350, Thermo Fisher Scientific), 3,3,5-triiodo-L-thyronine (T0821, Sigma-Aldrich), Dexamethasone (D175, Sigma-Aldrich), Cholera toxin (C9903, Sigma-Aldrich), Penicillin/streptomycin (15140122, Thermo Fisher Scientific), NU-Serum IV (355500, Thermo Fisher Scientific), Bovine pituitary extract (P1167, Sigma-Aldrich), Nicotinamide (N3376, Sigma-Aldrich), Primocin (ant-pm-2, InvivoGen), A83-01 (2939, Tocris), Recombinant Human Heregulin-1 (100-03, PeproTech), Rho Kinase Inhibitor (TB1254-GMP, Tocris).

### Mice

Male and female mice were used in all experiments with balanced sex ratios maintained across experimental groups to avoid sex-related confounding effects. For scRNA-seq analysis, sex ratios were unintentionally unbalanced between compared groups due to sample availability at the time of collection; sex-specific genes (*Xist*, *Ddx3y*) were therefore excluded from the analysis to account for this. Sex-disaggregated numbers (male/female breakdown per group) are provided in the Source Data file for all in vivo experiments. LSL-KRAS^G12D^-^23^ and LSL-Trp53^R172H^ are on mixed 129/SvJae/C57Bl/6 J background PDX-Cre mice on C57Bl/6 J were purchased from the Jackson Laboratory. Casp8^fl/fl^ mice^98^ on a C57BL/6 N background were obtained under a material transfer agreement (MTA) from Stephen Hedrick. MLKL^−/−^ mice on C57BL/6 N were newly generated in the Pasparakis lab and described in Körner et al^99^., RIPK3^−/−^ mice on C57BL/6 N background were obtained from Genentech under an existing MTA as part of the Pasparakis lab^48^. All mice were maintained on a 12-hour light/dark cycle at 20–22 °C ambient temperature and 50–60% humidity, with water and food ad libitum. For four consecutive weeks, 5-month-old mice were injected i.p. 2 x per week either with vehicle (PBS with 40% PEG-4000, 0.4% DMSO) or emricasan [2.5 mg/kg] with birinapant [5 mg/kg]. KC mice were sacrificed four weeks after the last treatment and the pathologist was blinded to the group allocation while performing the progression analysis. 8-week-old KPC mice were treated as described above and kept until humane experimental endpoint. For all Kaplan-Meier experiments experimental endpoints were chosen based upon a scoring system quantifying animal well-being based upon weight loss, general condition, abnormal behaviour as common practice in German animal experimentation. Animals were euthanised once the cumulative score reached 20. Scoring was performed by a person blinded to the study.

### Pathological Inspection and quantification of PanINs

All Samples were fixed in 4% buffered formalin for a minimum of 24 hours and a maximum of 72 hours, then transferred to paraffin. Three micrometer thin sections were prepared according to the standardised procedures at the Institute of Pathology and haematoxylin-eosin stained (H&E). A histopathologist with experience in the field of gastroenteropathology (AQ) evaluated all sections in a blinded manner. All primary ductal structures were identified and their various forms of intraepithelial Neoplasia of the pancreas (PanIN). PanINs were determined according to the current WHO classification (2019) and classified into low grade pancreatic intraepithelial neoplasia versus high grade pancreatic intraepithelial neoplasia. The extent of the PanINs relative to the total of available ductules was also quantified. The majority of the low grade PanINs found corresponded to the classic, simple mucin-filled columnar cells that completely or partially replaced the ductal epithelia. Furthermore, other histomorphological changes were determined: a) duct ectasia b) periductular fibrosis/stroma reaction c) extent and type of inflammation (low versus marked inflammation) (lymphocyte-dominated versus neutrophil-granulocyte dominated inflammation) d) invasive carcinoma.

### FACS analysis on pancreatic immune cell infiltrates

In order to isolate immune cells from the pancreas, whole tissue was dissected and minced with scalpels into fragments small enough to be aspirated into a 5 ml pipette at RT. 45 ml of tissue suspension was incubated with 5 ml of a 10× Triple Enzyme Mix (1 g Collagenase IV, 100 mg Hyaluronidase and 20,000 Units DNase IV into 80 ml HBSS) at RT for 30 min on a shaker at 80 rpm. Cell suspension was repeatedly pipetted to further dissociate cells, centrifuged at 50 *x g* at RT for 10 min and the supernatant was collected by passing it through a 70 μm nylon strainer. The bigger pellets at the bottom of the tube were then discarded and the filtered supernatant was centrifuged at 200 ×​​​​​​ ​*g* for 5 min. Cell pellets were washed with 10 ml Wash Buffer (1 g BSA and 2 ml 0.5 M EDTA in 800 ml HBSS) at 200 × *g* for 5 min once and were resuspended with 2 ml ACK lysis buffer (Gibco) for 1 min to deplete red blood cells. Cells were washed with PBS and immediately stained for live/dead cells using the viability dye eFluor660 (eBioscience) (1:1000) in PBS for 30 min, at 4 °C. Cells were then washed twice with FACS buffer (PBS, 2% FCS) and Fc block (CD16/32, biolegend, 1:50) was used 15 min. Cells were then stained with CD45-BV421 (30.F11) or CD45-FITC (30.F11) (biolegend), NK1.1- BV421 (PK136) (biolegend), CD4-V450 (RM4-5) (BD Horizon), CD11b-PE (M1/70) (ebioscience), CD8-PE (53-6.7) (biolegend), CD19- PE (1D3) (ebioscience), CD11c-BV421 (N418) (biolegend), Ly-6G/Ly-6C-FITC (RB6-BC5) (ebioscience), Gr1-BV-711 (RB6-8C5) (biolegend), CD14-FITC (Sa14-2) (biolegend), CCR3-FITC (J073E5) (biolegend), all at 1:1000 for another 30 min, at 4 °C. For subsequent intracellular stainings, cell pellets were resuspended in 200 μl Fixation/Permeabilization buffer (eBioscience) and incubated overnight at 4 °C. The next day, cells were washed with 1× permeabilisation buffer (eBioscience) and incubated for 15 min with 2% goat serum before adding CD206-BV421 (MMR) (C068C2) or Rat IgG2a, κ-isotype Ctrl -BV-421 (RTK-2758) (biolegend)1:50 for 30 min at 4 °C in 1× permeabilisation buffer. After washing twice with 1× permeabilisation buffer cells were resuspended in FACS buffer. Measurements were acquired using a BD LSR Fortessa flow cytometer and data were analysed using the FlowJo (10.6.1) software.

### Cells

LSL-KRAS^G12D^ inducible MEFs were generated as described previously^46^. “Rasless” MEFs reconstituted with either WT KRAS4B or KRAS^G12D^ (RPZ25854, RPZ26198) were generated and kindly provided by the RAS Initiative at the Frederick National Laboratory for Cancer Research (FNLCR), US. Rasless MEFs were grown in Dulbecco’s modified Eagle’s (DMEM) + GlutaMAX™ medium (Gibco) with 4 µg/ml of blasticidin. Freshly isolated LSL-KRAS^G12D^ inducible MEFs and IFNAR1^−/−^ MEFs were cultured in DMEM (Gibco) supplied with 1% L-Glutamine (Sigma) and 1% Sodium Pyruvate (Sigma). Control, STAT1, STAT2 and IRF9-deficient MEFs were previously published^73^ and kindly provided by Thomas Decker. Mouse PDAC cell lines (dox-KRASG12D mPDAC) derived from KC mice and engineered to express a doxycycline‑inducible additional KRAS^G12D^ allele^66^ were kindly provided by Roland Rad. Inducible human pancreatic duct epithelial cells (HPDE) pCW-KRAS^G12D^ were described previously^11^ and cultured in 75% RPMI 1640/ medium in presence of 25% keratinocyte growth medium 2 (PromoCell) + 0.5 µg Puromycin, BxPC-3 pCW-KRAS^G12D46^ in RPMI 1640 GlutaMAX™ + 1% Sodium Pyruvate (Sigma) + 2.5 µg Puromycin. Human PDAC cell lines BxPC-3, A818-6, HPAF-II, Capan-1, AsPC1, PancTul, Colo 357, Capan-2, PANC-1, PANC-89, MIA PaCa-2, PT45 were cultured in RPMI 1640 GlutaMAX™ + 1% Sodium Pyruvate (Sigma). All media were supplemented with 10% fetal calf serum (FCS) (Sigma Aldrich) and 1000 U/mL of both penicillin and streptomycin (Pen/Strep) (Sigma Aldrich). All cells were kept at 37 °C with 5% CO_2_ and tested for mycoplasma at regular intervals (mycoplasma barcodes, Eurofins Genomics). Human PDAC cell lines BxPC-3 (ATCC CRL-1687), HPAF-II (ATCC CRL-1997), Capan-1 (ATCC HTB-79), AsPC1 (ATCC CRL-1682), PANC-1 (ATCC CRL-1469), MIA PaCa-2 (ATCC CRL-1420), Capan-2 (ATCC HTB-80) were obtained from ATCC. A818-6 was kindly provided by A. Trauzold and described previously^100^. PancTul, Colo357, PANC-89 and PT45 were kindly provided by A. Trauzold and described previously^101^.

### Cell death assays (flow cytometry)

Four days before treatment 120,000 LSL-KRAS^G12D^ inducible MEFs (with or without 4OHT [1 µg/ml]), one day before treatment 500,000 cells (“Rasless” MEFs) were plated in each well of a 6-well plate. To determine cell death, adherent and detached cells were harvested and stained with propidium iodide (PI) [1 μg/ml] (Sigma Aldrich) or with Fixable Viability Dye eFluor™ 660 (eBioscience™) [1:1000] in PBS (Thermo Fisher) supplemented with 2% FBS. For staining with Annexin V (Biotium) [1 μg/ml] we used manufacturer’s protocol. Staining-positive cells were quantified by flow cytometry using an LSR-FACS Fortessa (BD Bioscience) and FlowJo software (BD Bioscience).

### Cytotoxicity/viability assay NYONE®

The cells were seeded at 1 × 10^4^ cells/well in 96-well plates. After 24 h, the cells were pretreated with Birinapant (1 µM; Selleck Chemicals, Houston, Texas, United States) or Birinapant (1 µM) and zVAD-fmk (20 µM, Bachem Holding, Bubendorf, Switzerland) for 1 h, followed by treatment with 100 ng/ml TRAIL (PeproTech, Hamburg, Germany). After 24 h cell viability and cell death were assessed by triple-fluorescence staining with Propidium Iodide (PI) (10 μg/ml, Invitrogen, Karlsruhe, Germany), Calcein AM (0.1 μg/ml, BioLegend, San Diego, California, United States) and Hoechst 33342 (2.5 μg/ml, Invitrogen). PI/Calcein AM/Hoechst 33342 staining was performed by diluting the substances in PBS and adding to the wells. The cells were incubated in the dark at 37 °C and 5% CO_2_ for 20 min and subsequently centrifuged for 5 min at 400 ×​​​​​​ *g*. Fluorescence imaging was performed by NYONE® SCIENTIFIC (SYNENTEC GmbH, Elmshorn, Germany) and the images were quantified using YT-software® (SYNENTEC GmbH): Cells stained with Hoechst 33342 and Calcein AM were considered living cells, whereas PI stained cells were counted as dead cells due to the loss of membrane integrity.

### Live cell imaging (IncuCyte)

Five thousand (“Rasless” MEFs) per 96-well plate were seeded 24 h in advance. Treatments were added in two parts: “pretreatment” everything except TNF or TRAIL or Fas Ligand and 30 minutes later cells are treated with ligands. Cells were imaged using the ×10 objective within the IncuCyte SX5 live cell imaging system (Sartorius). For dead cell quantification, 100 nM DRAQ7 (Thermofisher) were added to each well. Cells were imaged for the indicated timepoints every 2 h. Analysis for confluence, DRAQ7- positive (dead) cells was performed using the Software IncuCyte 2021 A (Sartorius).

### Mouse organoids

KC-organoids were generated from PDX1-Cre KRAS^G12D^ mice. The pancreas was washed with cold wash medium (DMEM high glucose, Pen/Strep, 1% FCS) and cut into 1–2 mm pieces. Pieces were digested in digestion medium (200 ml wash medium, 25 mg Collagenase P (C9407, Sigma-Aldrich), 25 mg Dispase II (17105041, Thermo Fisher) at 130 rpm at 37 °C in repeated cycles until wash fractions contained mainly pancreatic ducts. Duct-enriched fractions were centrifuged at 1200 rpm for 5 min, resuspended in ice-cold Matrigel (356231, Corning) and seeded as 30 µl domes in pre-warmed 24-well plates. After 15 min, 500 µl PancreaCult™ Organoid Growth Medium (06040, Stemcell) was added. Organoids were maintained with weekly splitting and twice-weekly medium changes. For treatment experiments, single cells from organoids were seeded at 250 cells in 100 µl of 10% Matrigel PancreaCult™ Organoid Growth Medium supplemented with Rho Kinase Inhibitor 10.5 µM (TB1254-GMP, Tocris) on solidified layer of 30 µM Matrigel:DPBS (1:1) per well in 96-well plates and grown for 6 days before treatment. Organoid viability was assessed using organoid brightness as a proxy using the BZ-X800E microscope and BZ-H4M/Measurement Application Software (Keyence).

### Patient-derived pancreatic cancer organoids (PDOs)

To evaluate the therapeutic strategy in patient-derived pancreatic cancer organoids, three distinct organoid lines were generated from EUS-guided fine needle aspiration/biopsy and surgical resection^102^. Biopsies were minced and surgery specimens were incubated rotating for collagen digestion using DMEM-F12, 1× Primocin and 6 mg/mL collagenase II. Tissue pellets were incubated with RBC lysis buffer and further digested using TrypLE. Cell pellets were resuspended in 50 µL Matrigel/well and PDO medium was added after Matrigel solidification. PDO medium consisted of DMEM-F12 supplemented with d-glucose, ITS Premix, triiodo-l-thyronine, dexamethasone, cholera toxin, penicillin/streptomycin, NU-Serum IV, bovine pituitary extract, nicotinamide, Primocin, A83-01, RSPO1-conditioned medium, Recombinant Human Heregulin-1 and Rho Kinase Inhibitor. The two male PDOs (#2 and #3) were previously established and published^103^ the additional female PDO (#1) was not published previously. All available clinical data on all PDOs are in the Supplementary Table 1. For organoid seeding, 500 single cells were plated in 30 µl of PDO medium containing 5% Matrigel in a 384-well white plate (#3570, Corning). After 24 hours, treatments were added in a total volume of 5 µl. Following an additional 30 minutes, 5 µl of medium—with or without 500 ng/ml human TRAIL—was added to each well. After 72 hours of incubation, cell viability was assessed using the CellTiter-Glo® 3D Cell Viability Assay (#G9681, Promega). For this, 8 µl of the reagent was added to each well containing 40 µl of total volume. Plates were shaken for 10 minutes and then incubated at room temperature for 20 minutes to stabilise the luminescent signal. Luminescence was measured using the VantaStar microplate reader (BMG Labtech).

### Western Blotting

The cells were washed with PBS and lysed with RIPA buffer (89901, Thermo Fisher), which was supplemented with phosphatase and protease inhibitors (Roche). The protein lysate concentrations were determined using the bicinchoninic acid (BCA) protein assay (50000113, Bio-Rad) and subsequently adjusted to the same concentration. Equal amounts of protein were mixed with a final concentration of 1× LDS sample buffer (NP0008, NuPAGE) and DTT (200 mM) and then heated to 80 °C for 10 min. The samples were separated via gel electrophoresis and transferred to nitrocellulose membranes (Bio-Rad). The membranes were blocked in PBS with 0.1% Tween 20 (PBST) with 5% BSA (Sigma-Aldrich) for at least 1 h and incubated with primary antibodies overnight at 4 °C. After washing with PBST, membranes were incubated with 1:10,000 diluted horse radish peroxidase (HRP)-coupled secondary antibodies for at least 1 h at room temperature. After another washing step, bound antibodies were detected using chemiluminescent Amersham ECL Prime Western Blotting Detection Reagent (RPN2235, Cytiva) or SuperSignalTM West Femto Maximum Sensitivity Substrate (34095, Thermo Fisher). The FUSION Solo S system and software (Vilber) were used to image the membranes.

### ELISA

CXCL2 (DY452-05, R&D systems), IFN-α (MFNAS0, R&D systems), IFN-ß (DY8234-05, R&D systems), IFN-γ (DY485-05, R&D systems), TNF (DY410-05, R&D systems), Fas ligand (DY526, R&D systems), TRAIL (DY1121, R&D systems) were used following the manufacturer’s instructions. Cell culture supernatants and murine pancreatic extracts were analysed after storage at − 80 °C. For mouse samples amounts of released protein was normalised to total protein level in the sample measured by DC protein assay kit (774985, Bio-rad). For the IFN-detecting ELISAs, cell culture supernatants were 16-fold concentrated using 3 kDa molecular weight cut-off spin columns (VS2092, Sartorius).

### Tissue stainings (immunohistochemistry and immunofluorescence)

Pancreatic tissues were fixed in 4% paraformaldehyde, embedded in paraffin, and cut into 3–5 µm sections. Paraffin sections were rehydrated by passing the slides through xylene and descending grades of alcohol then rinsed in water. The slides for IHC were incubated for 15 minutes with Peroxidase Blocking Solution (SP-6000, Vector Laboratories). Then heat-induced antigen retrieval was performed in citrate buffer (H-3300, Vector Laboratories) in a pressure cooker (110 °C) for 1.5 h or by proteinase K treatment for 15 min at 37 °C (only CD45). Slides were then immediately cooled under running water and rinsed in PBS. 100 μl of Protein Block (ab64226, Abcam) was added to each slide for 45 minutes. After washing 3× with 0.05% PBS/Tween 20 solution for 5 minutes, the slides were incubated with 100 μl of the primary antibody at 4 °C overnight in a humidity chamber. Following overnight incubation, slides were washed with 0.05% PBS/Tween 20 solution. The sections were then incubated with secondary antibody for 1 h at room temperature and again washed three times. For IHC, the sections were developed using the ABC kit (PK-6100, Biozol) and consequently incubated with DAB substrate before being counterstained with haematoxylin and rinsed in water for 1 minute. Slides were then dehydrated in ascending grades of alcohol and cleared in xylene (IHC). Finally, the sections were mounted using Di-N-Butyl Phthalate in Xylene (DPX) mounting solution and covered with a glass coverslip (IHC) or mounted in DAPI (ProLong® Golds antifade reagent with DAPI, Invitrogen) (IF). For negative controls, adjacent duplicate slides from each case were used. These slides were incubated with 100 μl antibody diluent instead of primary antibody/secondary antibody. H&E stainings were examined by an experienced pathologist (A.Q.) who was blinded to the study design. TUNEL stainings were performed according to manufacturer’s protocol (Promega, G3250) for paraffin embedded tissue. The propidium iodide step was omitted, and slides were instead covered with ProLong™ Gold Antifade mounting medium with DAPI (Invitrogen™, P36941). The Trichrome staining was performed according to the manufacturer’s protocol of the Trichrome Stain (Masson) Kit (HT15-1KT, Sigma-Aldrich). Fluorescence pictures were acquired using the Keyence BZ-X800 microscope and analysed with the BZ-X800 Analyser software. Cytokeratin 19 and CD45 IHC stainings were analysed and quantified using the QuPath software.

### Immunofluorescence microscopy

Ten thousand MEFs per well were seeded in 24 well plates on top of a sterilised glass coverslip and incubated for 24 hours with or without 10 nM of the KRAS inhibitor MRTX1133. After the incubation the cells were washed with PBS and fixed with 4% paraformaldehyde for 15 min in room temperature. After fixation cells were treated with DAPI (1:5000) for 1 hour in room temperature and washed three times with PBS. Fluorescence pictures were acquired using confocal microscopy (SP8 Confocal Microscope, Leica).

### siRNA transfections

Two hundred microliters Opti-MEM (Gibco) and 1.5 µL Dharmafect Reagent I (Dharmacon) were mixed and incubated for 5–10 min at room temperature. 2.8 µL of siRNA (Stock 20 mM) (Dharmacon) were added to the mixture and incubated for another 30 min at room temperature. After incubation, 200 µL of the mixture was added to each well (6-well) plate and cells were plated on top. Knockdowns were incubated for 72 h, as indicated.

### Quantitative real-time PCR (qPCR)

The NucleoSpin RNA kit (740955.5, Macherey-Nagel) was used to isolate total RNA following the manufacturer’s instructions. The isolated RNA was then converted to cDNA using the LunaScript RT SuperMix Kit (E3010L, New England Biolabs). For qPCR each reaction contained 5 µl of Luna Universal qPCR Master Mix (M3003E, New England Biolabs), 2 µl of nuclease-free water, 1 µl (10 µM) of primer mix (forward and reverse primers), and 2 µl of cDNA (10 ng/µl). qPCR was performed in quadruplicates on the Quant Studio5 qRT PCR cycler. All results were normalised to the expression of the housekeeping gene control *Rpl13a*. Primer sequences used can be found in Supplementary Data 3.

### Bulk RNA-seq analysis

The RNA-seq datasets were analysed on the CHEOPS HPC cluster of the University of Cologne using the RNA-Seq pipeline of the nf-core suite v3.7^104^ with default parameters and Nextflow v21.10.6^105^. For instance, the reads were aligned to the mm10 reference genome sequence using STAR v2.7.10a^106^ with default parameters. In the quantification step, reads were counted with the quasi-mapping quantification tool Salmon v1.5.2^107^ Differential expression analysis (DEA) was then performed in R using the DESeq function of the DESeq2 v1.36.0 package^108^, with default parameters. Genes with absolute value of log2 fold change ≥0.56 and adjusted *p* value ≤ 0.05 were considered differentially expressed.

### Bioinformatic analysis of human TCGA datasets

Processed RNA-sequencing data of TCGA patient cohorts was downloaded using the R packages GenomicsDataCommons and TCGAutils on 2023-01-12. Interferon-stimulated gene (ISG) expression signatures were calculated using a published approach and Interferon gene set^68^. In brief, to derive Interferon signature scores, median absolute deviation (MAD) Z-scores of log2-transformed and Transcript Per Million (TPM)-normalised mRNA expression values were calculated per gene. The mean of all signature genes’ Z-scores per sample was subsequently defined as the ISG score. Categories of high or low ISG score were defined by the mean ISG score +/− 1 standard deviation, remaining samples were considered having medium ISG score and log2-TPM expression of candidate genes compared between groups. For survival analysis of PAncreatic ADenocarcinoma (PAAD), patients were grouped by Caspase 8 expression as low (lowest 10%) or high Caspase 8 (highest 90%). Kaplan–Meier analysis was performed between both groups. Events after 5 years of follow-up were censored to minimise artifacts by competing risks. The Ras84 gene set was downloaded from East et al.^38^ and every gene within the gene set was assigned a value corresponding to the rank of its expression level within each sample. For each sample, the Ras84 score is then calculated as the median of the ranks of the 84 genes. For each tumour cohort, the Ras84 score was calculated as the median of the Ras84 scores of individual samples. The necroptosis score was calculated as the sum of the expression levels of *Mlkl*, *Ripk3*, and *Zbp1* after log10 (TPM + 1) transformation. For each tumour cohort, the necroptosis score was calculated as the median of the necroptosis score of individual samples. Pearson correlation was used to assess the relationship between computationally derived scores across TCGA datasets. Mutation data was downloaded from the TCGA repository on 2023-05-03 and cohorts with a frequency of missense mutations in KRAS greater than 25% were marked as high-frequency KRAS tumour types.

TCGA analyses included 10,534 samples across 33 cancer types for pan-cancer analyses, 507 samples for PAAD/GTEx correlation analyses, and 178 patients for survival analyses. Cell line Ras84 scores were computed for 12 human PDAC cell lines. Sample sizes separated for each cancer type for all TCGA analyses are provided in the Source Data file.

### Single-cell sample preparation for scRNA-seq of KC mice samples and fluorescent cell sorting

Mouse pancreata harvested from 5-month-old mice was cut into ~3 mm pieces, incubated with 5 ml of digestion medium (200 mg/l Dispase (17105041, Thermo Fisher), 200 mg/l collagenase P (11249002001, Merck) in DMEM (Gibco) containing Pen/Strep and 1% FCS) for 20 min 37 °C while shaking at 150 rpm. Tissue was processed with gentleMACS™ Octo Dissociator imp.tumor3 program. Then cells were strained through a 70 µm strainer, spun down at 300 × *g* for 7 minutes. Blood cells were removed with Red Blood Cell Lysis Solution (Miltenyi Biotec 130-094-183). Cells were then washed with 0.04% BSA (9048-46-8, Thermo Fisher) in PBS, strained through a 40 µm tissue strainer and resuspended in 0.04% BSA in PBS for scRNA-seq. For fluorescent cell sorting, single cells were prepared as described above, washed in PBS and immediately stained for live/dead cells using the viability dye eFluor660 (eBioscience) (1:1000) in PBS for 30 min, at 4 °C. Cells were then washed twice with FACS buffer (PBS, 2% FCS) and incubated with Fc block (CD16/32, biolegend, 1:50) for 15 min. Then cells were stained with CD45-APC-Cy7 (30.F11) (biolegend), washed and CD45^-^ cells with or without GFP signal were sorted to obtain KRAS^G12D^- or KRAS^WT^-expressing cells, respectively. Cells were spun down at 200 × *g* for 5 min and the cell pellet was used for RNA extraction (according to manufacture instructions 740902.10, Macherey Nagel) and further qPCR analysis.

### Single cell RNA-sequencing (scRNA-seq) bioinformatic pipeline for KC mice samples

#### Preprocessing

Bioinformatics analysis of single-cell RNA sequencing data was conducted as described previously^109^ using the reproducible Common Workflow Language pipelines available on Scientific Data Analysis Platform (SciDAP). Briefly, raw FASTQ files (PRJNA975357) were independently mapped to the mm10 (2020-A from July 7, 2020) reference genome by the Cell Ranger Count (version 4.0.0) pipeline. Produced gene expression profiles per sample were aggregated into a single feature-barcode matrix by running Cell Ranger Aggregate (version 4.0.0) pipeline with disabled depth normalization parameter. All other data analysis workflows described below used the Seurat (version 4.1.0) R package.

#### Low quality cell removal

Low quality cells were removed in two iterations. First, the following filtering thresholds were applied per cell – from 80 to 6000 genes, minimum 500 uniquely mapped fragments, maximum 5 percent of transcripts mapped to mitochondrial genes. Preliminary filtered data were run through the dimensionality reduction pipeline to integrate all samples into a single UMAP. The latter allowed us to identify the red blood cells based on the expression of the marker genes (gene names) and manually exclude them on the next iteration of the filtering pipeline.

#### Datasets integration and clustering

High quality cells from all samples were first processed by dimensionality reduction pipeline. On this step gene expression data from each sample were normalised and scaled using SCTransform function. When scaling, the expression levels of the following genes – *Xist* and *Ddx3y*, were set as variables to regress out. Normalised data were then integrated following the instructions from the official Seurat vignette using the first 20 dimensions for principal component analysis (PCA) and UMAP projection. Next, cluster analysis workflow was run on the PCA reduced data using 20 dimensions and 0.1 clustering resolution. Gene markers were identified for each cluster using FindAllMarkers function with default parameters (Wilcoxon rank-sum test, two-sided, with Bonferroni correction for multiple comparisons), returning only upregulated genes. Based on the identified gene markers the cell types were assigned. For the analysis of only immune cells, dimensionality reduction and clustering pipelines were run on the prefiltered cells using 0.2 clustering resolution.

#### Pseudobulk differential gene expression analysis

To identify differences in gene expression profiles between two groups of samples (KC-C8^wt/wt^ and KC-C8^fl/fl^) pseudobulk differential gene expression pipeline was run for each identified cell type. Cells were first prefiltered to belong only to the specific cell type, then aggregated to a pseudobulk form within each sample. Ribosomal, mitochondrial, as well as *Xist* and *Ddx3y* genes were excluded from the analysis. Aggregated raw reads counts were processed by DESeq2 as bulk RNA sequencing data. The results were filtered to include only differentially expressed genes with p adjusted values not bigger than 0.05.

### Single cell analysis of published human scRNA-seq data

The human primary PDAC and normal pancreas scRNA-seq dataset^67^ was downloaded from GSA: CRA001160. For all samples, only ductal cell 1 and 2 were extracted based on cell type annotation. Data was then normalised by SCTransform. The first 15 PCAs are used for clustering and UMAP projection. Expression of each gene is plotted by Seurat FeaturePlot function.

### Statistics & reproducibility

All data were analysed by GraphPad Prism Software using *t* test, one-way ANOVA or two-way ANOVA as indicated. Results were considered significant at *p* < 0.05. For RNA-seq differential expression analysis, genes with adjusted *p* value ≤ 0.05 and fold change ≥ 0.56 were considered differentially expressed. In vitro and in vivo results are presented as means ± SEM calculated from at least three independent biological repeats (each performed with at least two technical replicates) unless otherwise noted. All statistical tests used were two-sided. Numbers are indicated in the figure legends and in Source Data file. Numbers of mice are stated within figures or figure legends in all cases. No statistical method was used to predetermine sample size. Mitochondrial genes and sex-specific genes (*Xist*, *Ddx3y*) were excluded from scRNA-seq analysis to account for mixed-sex samples; no other data were excluded from the analyses. In vivo experiments were randomized with mice randomly assigned to treatment groups while maintaining balanced sex ratios; randomization was not applicable to cell-based experiments. The investigators were not blinded to allocation during experiments and outcome assessment, except for histological analysis which was performed by a pathologist blinded to the experimental groups. Western blot, immunofluorescence, immunohistochemistry, and organoid images shown are representative of at least three independent experiments with similar results unless otherwise stated in the figure legend. Molecular weight markers in kDa are indicated on all Western blot panels.

### Reporting summary

Further information on research design is available in the Nature Portfolio Reporting Summary linked to this article.

## Supplementary information

Supplementary Information

Description of Additional Supplementary Files

Supplementary Data

Reporting Summary

Transparent Peer Review file

## Source data

Source data

## Data Availability

The single-cell RNA-seq datasets generated in this study are available in Sequence Read Archive (SRA) under the accession code PRJNA975357 and the bulk RNA-seq datasets generated in this study are available on SRA accession code PRJNA975358. Source data are provided with this paper. The remaining data are available within the Article, Supplementary Information or Source Data file. Source data are provided with this paper.
