# Supplementary material for: Oncogenic KRAS-driven type I interferon signalling primes pancreatic cancer for necroptosis
Source: Nat Commun. 2026 Jun 15;17:5288. doi: 10.1038/s41467-026-73189-8 (PMC13269921; doi:10.1038/s41467-026-73189-8)
Supplement: Supplementary file 1 — Supplementary Information [file 41467_2026_73189_MOESM1_ESM.pdf]

Supplementary Information

Oncogenic KRAS-Driven type I Interferon Signalling Primes Pancreatic Cancer for Necroptosis

Sofya Tishina[1, 2], Alina Dahlhaus[1, 2], Marta Manik[1, 2], Lejla Mulalic[1, 2], Janine Murr[3,4], Michael Kotliar[5], Hassan Rakhsh-Khorshid[6], Myrto Kostopoulou[1, 2], Florian Hocher[7], Jenny Stroh[1, 2], Julia Beck[1, 2], Riley M. Willliams[8], Gülce G. Balta[1, 2], Fanyu Liu[1, 2], Ali T. Abdallah[2,9], Christina M. Bebber[1, 2], Moritz Reese[1, 2], Jonathan K. M. Lim[10], Alexander Quaas[11], Johannes Brägelmann[1, 2, 12, 13], Manolis Pasparakis[2, 13, 14], Filippo Beleggia[1, 2], Siddharth Balachandran[8], Anna Trauzold[7, 15], Gianmaria Liccardi[6], Igor Astsaturov[16], Maximilian Reichert[3,5,17,18], Ariadne Androulidaki[1, 2] & Silvia von Karstedt[1, 2, 13, #]

[1]University of Cologne, Faculty of Medicine and University Hospital Cologne, Department of Translational Genomics; Cologne, Germany.
[2]CECAD Cluster of Excellence, Faculty of Medicine and University Hospital Cologne; Cologne, Germany.
[3]Translational Pancreatic Cancer Research Centre, TUM School of Medicine and Health, Department of Clinical Medicine – Clinical Department for Internal Medicine II, TUM University Hospital, Technical University of Munich, Munich Germany
[4]Centre for Organoid Systems (COS), Technical University Munich (TUM), Garching, Germany.
[5]Cincinnati Children's Hospital Medical Center, Division of Allergy and Immunology, Cincinnati, USA
[6]University of Cologne, Faculty of Medicine and University Hospital Cologne, Centre for Biochemistry, Cologne, Germany.
[7]University of Kiel, Institute for Experimental Cancer Research, Kiel, Germany
[8]Fox Chase Cancer Center, Cancer Signaling and Microenvironment Program, Philadelphia, USA
[9]Institute of Medical Statistics and Computational Biology, Faculty of Medicine, University of Cologne; Cologne, Germany.
[10]Heinrich Heine University, Medical Faculty and University Hospital Düsseldorf, Institute of Neuropathology, Düsseldorf, Germany.
[11]University of Cologne, Faculty of Medicine and University Hospital Cologne, Institute of Pathology, Cologne, Germany.
[12]University Hospital Cologne, Mildred Scheel School of Oncology, Cologne, Germany.
[13]University Hospital Cologne, Centre for Molecular Medicine Cologne (CMMC), Cologne, Germany.
[14]University of Cologne, Institute for Genetics, Cologne, Germany
[15]University Hospital Schleswig-Holstein (UKSH), Department of Gynecology and Obstetrics, Campus Kiel, Kiel, Germany.
[16]Fox Chase Cancer Center, Molecular Therapeutics Program, Philadelphia, USA
[17]German Cancer Consortium (DKTK), partner site Munich, a partnership between DKFZ and TUM University Hospital, Germany.
[18]Bavarian Cancer Research Centre (BZKF), Munich, Germany

[#]Corresponding author: S von Karstedt, E-mail: s.vonkarstedt@uni-koeln.de

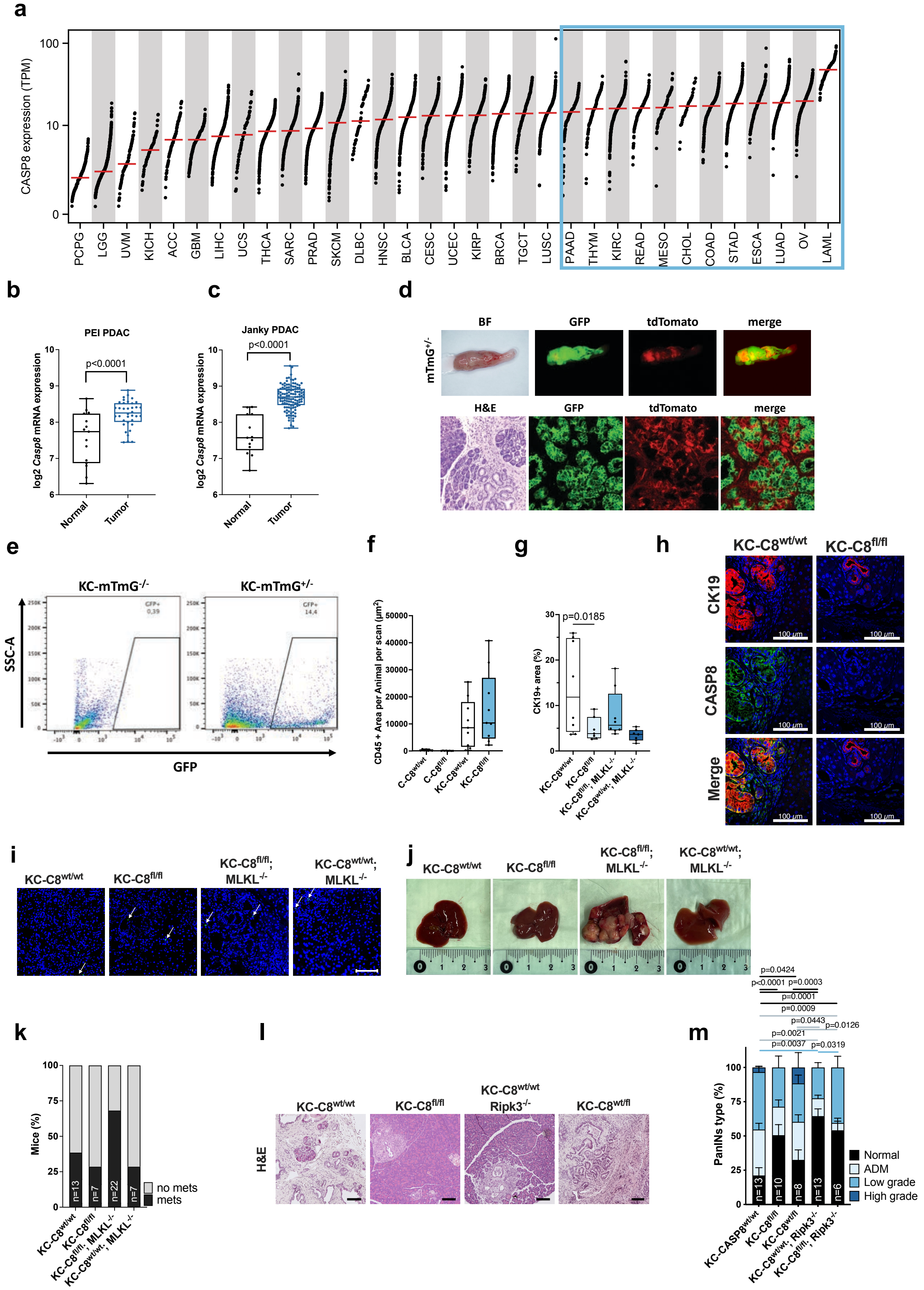

Supplementary Figure 1. Caspase 8 expression is elevated in PDAC.

a, Caspase 8 expression is plotted as transcript per million (TPM) across the cancer genome atlas (TCGA) datasets. **b, c,** Log^2-transformed caspase 8 mRNA expression is plotted in PDAC as compared to adjacent normal pancreas two independent cohorts (**b**[20]) and (**c**[19]). Boxplot centre line, mean; box limits, upper and lower quartile; whiskers min. to max. **d,** Macroscopic and microscopic acquisition of KC-ROSA26^{mTmG} mouse pancreata. **e,** Representative result of GFP^{+} fluorescent cell sorting of KC-ROSA26^{mTmG}. **f,** C- C8$^{wt/wt}$ (n=7), C- C8$^{fl/fl}$ (n=8), KC-C8$^{wt/wt}$ (n=9) and KC-C8$^{fl/fl}$ (n=9) sections were stained for CD45 using immunohistochemistry and quantified using QuPath. Boxplot centre line, mean; box limits, upper and lower quartile; whiskers min. to max. **g,** Pancreata from 5-months-old KC-C8$^{wt/wt}$ (n=8), KC-C8$^{fl/fl}$ (n=7), KC-C8$^{fl/fl}$;MLKL$^{-/-}$ (n=8) and KC-C8$^{wt/wt}$;MLKL$^{-/-}$ mice (n=7) were stained for CK19 using immunofluorescence and immunohistochemistry, CK19^{+} area was quantified using QuPath. Boxplot centre line, mean; box limits, upper and lower quartile; whiskers min. to max. **h,** 5-months-old mice of the indicated genotypes were sacrificed and pancreata were excised, fixed and stained for caspase 8 using immunofluorescence, representative images are shown. **i,** Representative images of 5-months-old mice with indicated genotypes are stained by TUNEL assay. **j,** Photos of representative livers are shown. **k,** Percentage of macroscopic liver metastasis was quantified at experimental endpoint. **l, m,** 5-months-old KC-C8$^{wt/wt}$ (n=13), KC-C8$^{fl/fl}$ (n=10), KC-C8$^{wt/fl}$ (n=8), KC-C8$^{wt/wt}$ Ripk3$^{-/-}$ (n=13) and KC-C8$^{fl/fl}$ Ripk3$^{-/-}$ (n=6) mice were sacrificed, pancreata were excised, fixed and stained by H&E. (**l**) Representative images are shown. Size bars represent 100 μm. (**m**) Percentage of ducts visible per section from m were graded and quantified by blinded pathological inspection. Data are means +/- SEM. Unpaired t-test (b, c), ordinary one-way ANOVA (f, g), and two-way ANOVA (m) were used. Source data are provided as a Source Data file.

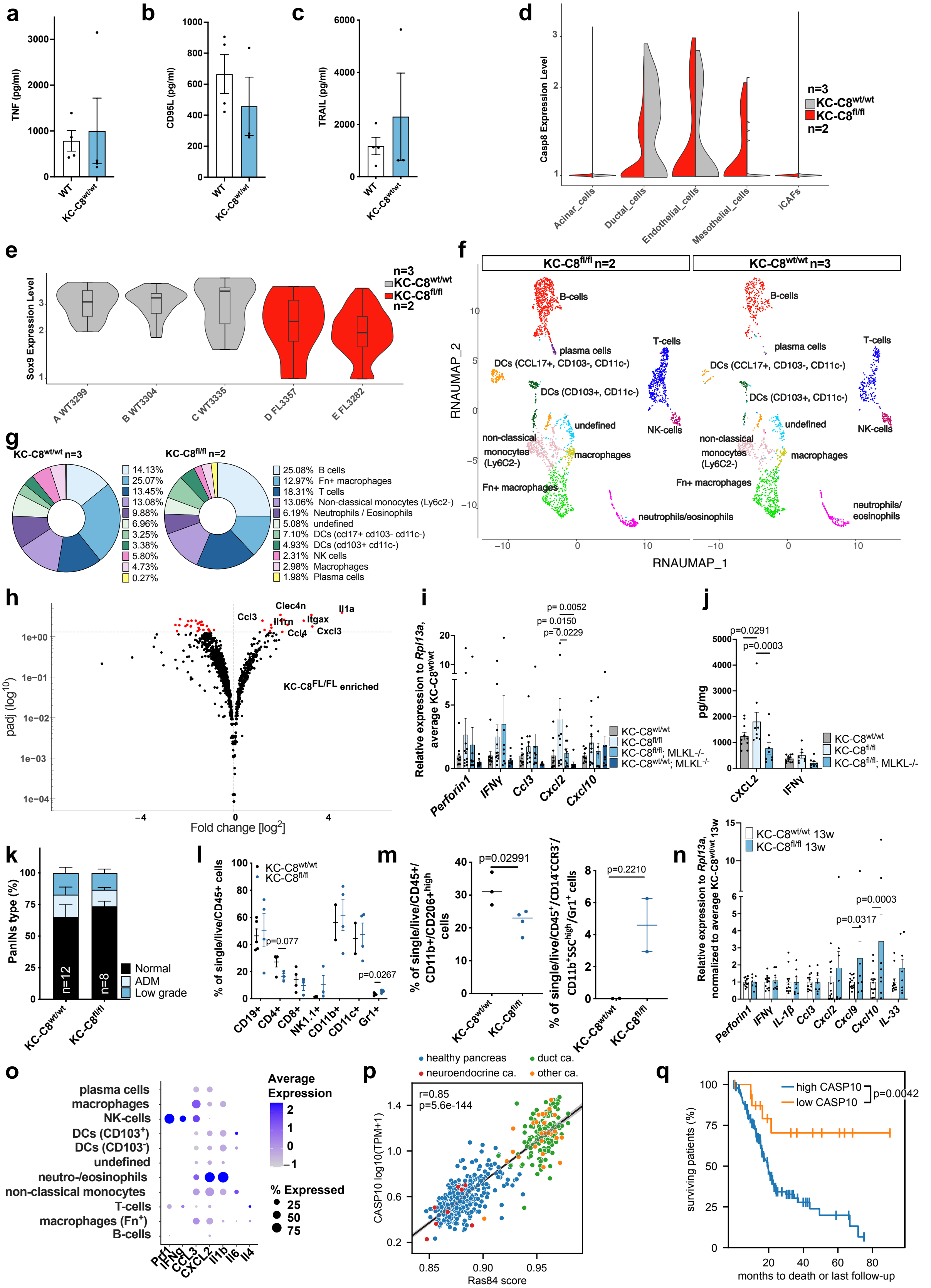

Supplementary Figure 2. Caspase 8 deletion promotes inflammatory infiltration.

a-c, Pancreata from 5-months-old C-C8$^{wt/wt}$(n=4), KC-C8$^{wt/wt}$ (n=4) were isolated and protein extracts were subjected to ELISA for the quantification of the indicated secreted proteins. **d,**

Log normalised caspase 8 expression density per cell type (all $CD45^-$ cell types) is shown split by grouping condition in scRNA-seq datasets from 5-months-old KC-C8$^{wt/wt}$ and KC-C8$^{fl/fl}$ mice. **e,** Log normalised *Sox9* expression per individual mouse within ductal cells of 5-months-old KC-C8$^{wt/wt}$ and KC-C8$^{fl/fl}$ mice. **f, g,** $CD45^+$ cells were reclustered within scRNA-seq data using RNA UMAP. (**g**) The average percentage of the indicated immune cell population split by genotype within $CD45^+$ cells is shown. Data are plotted as means in a pie-chart. **h,** The top 20 (by p adjusted) differentially expressed genes within the neutrophil/eosinophil cluster are shown. **i,** Pancreata from 5-months-old KC-C8$^{wt/wt}$ (n=8), KC-C8$^{fl/fl}$ (n=8), KC-C8$^{fl/fl}$;MLKL$^{-/-}$ (n=8) and KC-C8$^{wt/wt}$;MLKL$^{-/-}$ (n=8) were isolated and RNA extracted. The indicated cytokines were detected within bulk RNA using qPCR. Relative expression of genes normalised to ribosomal housekeeping gene *Rpl13a*. **j**, Protein extracts from rom 5-months-old KC-C8$^{wt/wt}$ (n=10-11), KC-C8$^{fl/fl}$ (n=8), KC-C8$^{fl/fl}$;MLKL$^{-/-}$ (n=8) were subjected to ELISA for the quantification of the indicated secreted proteins. **k,** 3-months-old KC-C8$^{wt/wt}$ (n=12) and KC-C8$^{fl/fl}$ (n=8) mice were sacrificed, pancreata were excised, fixed and stained by H&E. % of ducts visible per section were graded and quantified by blinded pathological inspection. **l, m** (**l**) Immune populations of 3-months-old KC-C8$^{wt/wt}$ (n=3-4) and KC-C8$^{fl/fl}$ (n=3-4) mice were analysed using FACs. The indicated immune populations are shown as percentage of viable immune cells. (**m**) Subpopulation of M2-macrophages (CD11b+CD206+high) and neutrophils (CD14-CCR3-/CD11b+SSChigh/Gr1+) within 3 months-old KC-C8$^{wt/wt}$ (n=3 and 2, respectively) and KC-C8$^{fl/fl}$ (n=4 and 2, respectively). **n,** Pancreata from 3-months-old KC-C8$^{wt/wt}$ (n=13), KC-C8$^{fl/fl}$ (n=8) were isolated and RNA extracted. The indicated cytokines were detected within bulk RNA using qPCR. **o,** Cell type-specific expression of the indicated genes is shown as log1p-transformed scaled average gene expression (scale factor 10000) within $CD45^+$ KC-C8$^{wt/wt}$ (n=3) single cells. **p,** Caspase 10 expression levels are plotted as a function of the Ras84 score [37] in the PAAD TCGA and normal human pancreas (GTEx) datasets. **q,** Kaplan Meier survival of the 10th percentile lowest caspase 10 expression vs. the rest within the PAAD dataset (TCGA) is plotted. Data are means +/- SEM. Unpaired t-test (a-c, l, m, n) and two-way ANOVA (i-k), logrank test for survival analysis (q). Source data are provided as a Source Data file.

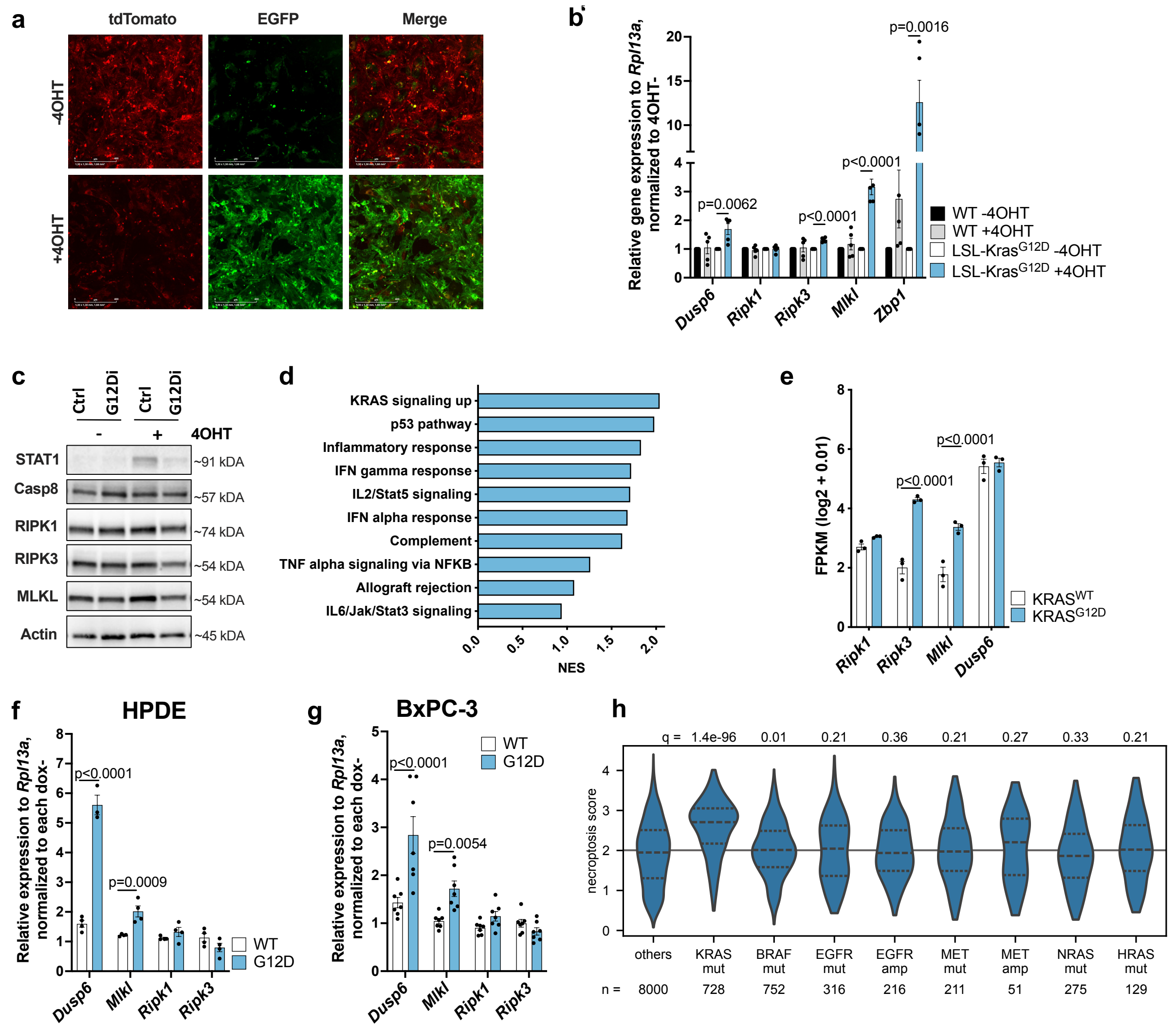

Supplementary Figure 3. Oncogenic KRAS induces necroptosis pathway component expression.

a, LSL-KRAS^{G12D};mTmG MEFs were treated with 4OHT [1µM] for 96 h. Fluorescent images were acquired using the IncuCyte SX5 live cell imaging system. **b**, LSL-KRAS^{G12D};mTmG MEFs were treated with control or 4OHT [1µM] for 96 h (n=5). Expression of the indicated genes was quantified by qPCR. Relative expression of genes normalised to ribosomal housekeeping gene *Rpl13a*. **c,** LSL-KRAS^{G12D};mTmG MEFs were treated with G12D inhibitor (MRTX1133) [10nM] and 4OHT [1µM] for 96h. Representative Western blots are shown. **d,** A ranked list from the RNA-seq data from KRAS^{G12D} versus KRAS^{WT} MEFs (Rasless MEFs constitutively reconstituted) was subjected to gene set enrichment analysis (GSEA). Significant normalised enrichment score (NES) is shown for all hallmark gene sets within the KRAS^{G12D}group. **e,** Log^2-transformed expression (fragments per kilobase of transcript per million fragments mapped, FPKM) of the indicated genes in cells as in d is shown (n=3). **f, g,** Doxycycline inducible KRAS^{WT} or KRAS^{G12D} HPDE (**f**, n=4) or Bxpc3 (**g**, n=7) cells were

treated with control or doxycycline [1mg/ml] for 48h, RNA was collected, and expression of the indicated genes was quantified using qPCR. **h,** A necroptosis score was computed based upon combined *Ripk3*, *Mlkl* and *Zbp1* expression and correlated to TCGA data of cancers with the indicated mutations or amplifications, related to the MAPK pathway. Relative expression of genes normalised to ribosomal housekeeping gene *Rpl13a*. Data are means +/- SEM. Unpaired t-test with Holm-Sidak (b) and with Benjamini Krieger and Yekultieli (e-g) correction were used. Source data are provided as a Source Data file.

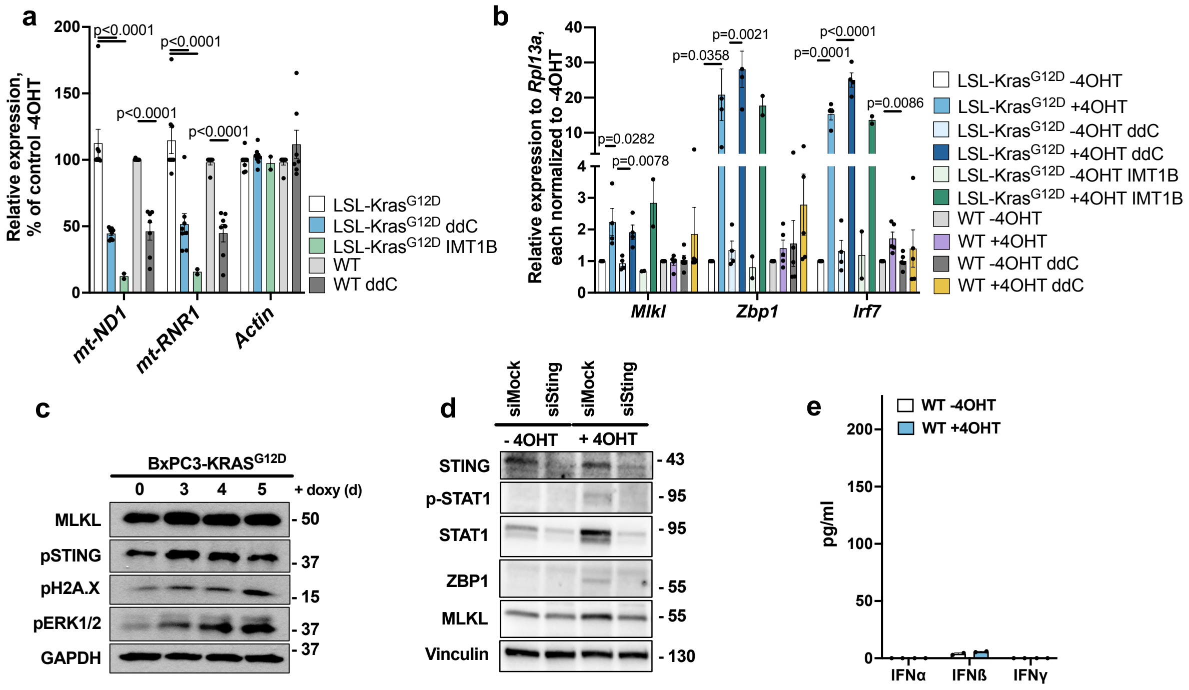

Supplementary Figure 4. Oncogenic KRAS induces necroptosis pathway component expression in a STING-dependent manner.

a, b, LSL-KRASG12D or MEFs were pre-treated with ddC [150 µM] or IMT1B [10 µM] for 7 days. Subsequently, half of the cells were further treated with 4OHT [1µM] for an additional 96 hours. (**a**) Genomic DNA was extracted after 7 days, and the expression of mitochondrial and control genes was analysed (n=8, 8, 2, 7, 7, top to bottom). (**b**) After the additional 96-hour treatment, RNA was extracted and qPCR was used to analyse the expression of the specified genes (n=4, 4, 4, 4, 2, 2, 5, 5, 5, 5, top to bottom). **c**, Doxycycline inducible $KRAS^{WT}$ or $KRAS^{G12D}$ BxPC-3 cells were treated with control or doxycycline [1mg/ml] for indicated number of days. Representative Western Blots for the indicated proteins are shown. **d,** LSL-$KRAS^{G12D}$ MEFs were pretreated together with siMock or siSting siRNA pools for 48h, followed by treatment with or without 4OHT [1µM]. Cells were collected after 96h incubation

with or without 4OHT. Representative Western Blots for the indicated proteins are shown. **e,** WT MEFs were treated with or without 4OHT [1µM] for 96 h, followed by 16-fold supernatant concentration using 3 kDa molecular weight cut-off spin columns and supernatants were subjected to ELISA quantification of the indicated proteins (n=2 for each). Data are means +/- SEM. Two-way ANOVA (a, b, e) was used. Source data are provided as a Source Data file.

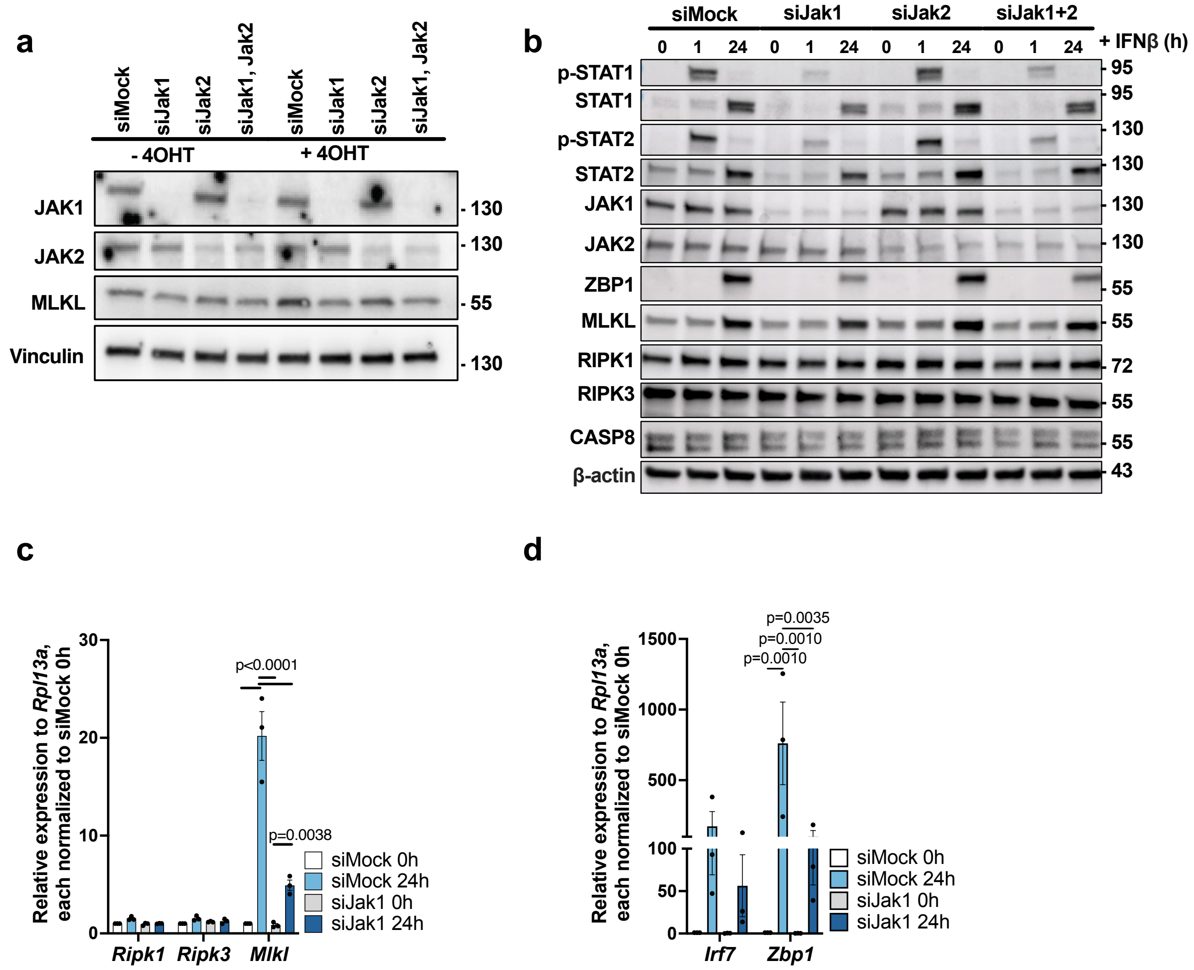

Supplementary Figure 5. Necroptosis-associated interferon stimulated genes (ISGs) are induced in a JAK1/STAT1/2/IRF9-dependent manner.

a, LSL-KRAS^{G12D} MEFs were treated with or without 4OHT [1µM] together with *Jak1* or *Jak2* or combined siRNA-mediated knockdown. Cells were collected after 96h incubation. The indicated proteins were detected by Western blotting. **b-d,** WT MEFs were pretreated together with Mock or *Jak1* or *Jak2* or combined siRNA-mediated knockdown for 48h, then treated with control or IFNß [100ng/ml] for indicated times (n=3). Expression of the indicated genes was quantified by Western blot and qPCR. Relative expression of genes normalised to ribosomal housekeeping gene *Rpl13a*. Data are means +/- SEM. Two-way ANOVA (c, d) was used. Source data are provided as a Source Data file.

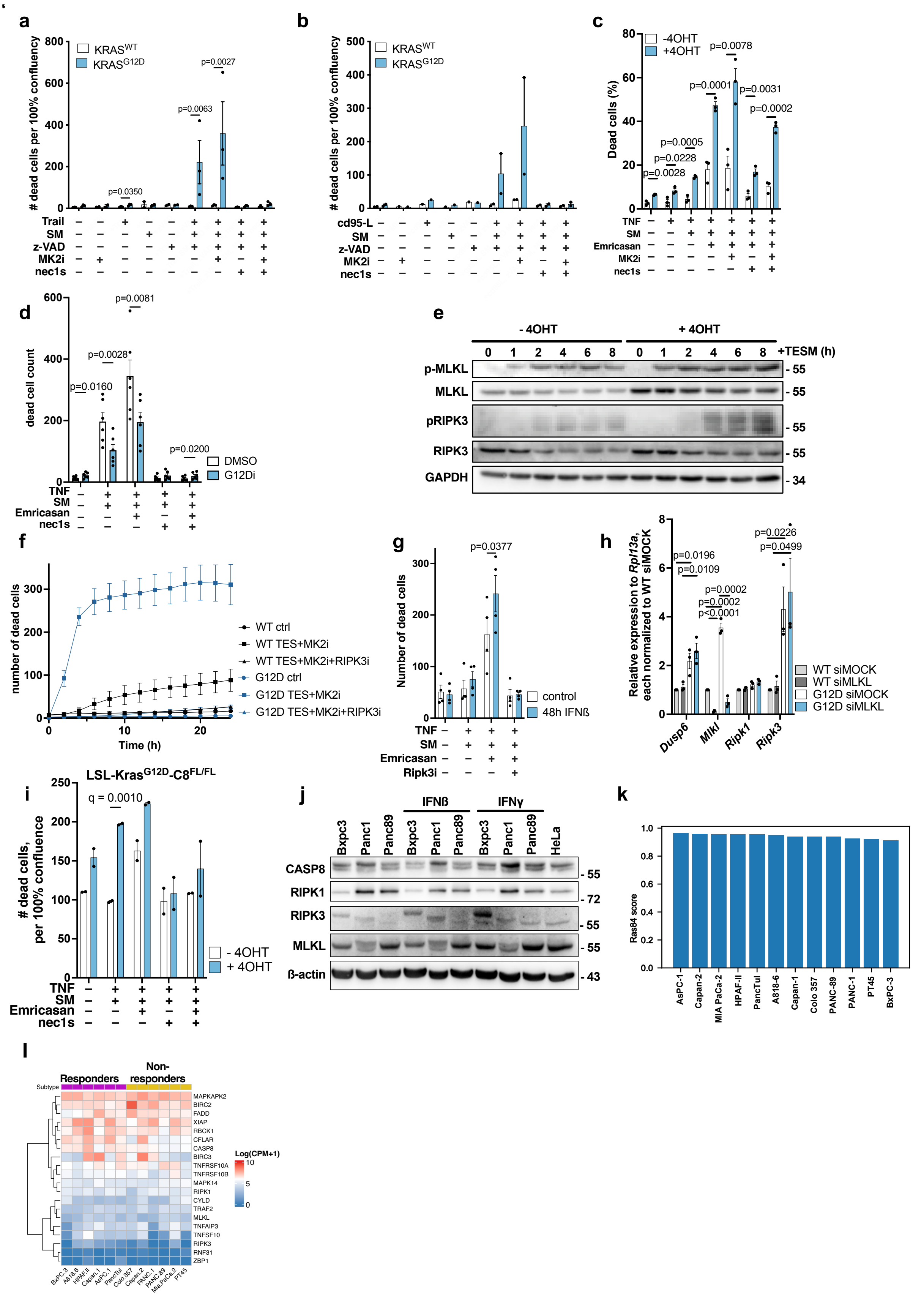

Supplementary Figure 6. Necroptotic priming sensitises KRAS-mutated cells to necroptotic cell death.

a, b, $KRAS^{G12D}$ and $KRAS^{WT}$ MEFs (Rasless MEFs constitutively reconstituted) were treated with (SM, birinapant [1 µM], z-VAD [20 µM], MK2i [20 µM], nec1s [10 µM], TRAIL [1 µg/ml] (n=3) or CD95-L [1 µg/ml] (n=2). Cell death was analysed by DRAQ7 fluorescence with normalization to cell confluence using the IncuCyte S3 bioimaging platform. **c,** LSL-$KRAS^{G12D}$ or WT (ERT2_Cre) MEFs were treated with control or 4OHT for 96h followed by the indicated treatment combination for 24h TNF [50ng/ml], SM [1µM], emricasan [2.5µM], MK2i [10µM] and RIPK1i [10µM]. Dead cells (eFluor660^{+} cells) were quantified by flow cytometry (n=3). **d,** Mouse PDAC cells treated with $KRAS^{G12D}$ inhibitor (G12Di) [MRTX1133, 10nM] for 2 days prior treatment (SM, birinapant [1 µM], emricasan [2.5 µM], nec1s [10 µM], TNF [50ng/ml]) for 24h (n=6). Cell death was analysed by DRAQ7 fluorescence with normalization to cell confluence using the IncuCyte S3 bioimaging platform. **e,** LSL-$KRAS^{G12D}$ MEFs were seeded with or without 4OHT [1µM] 4 days prior, then treated for the indicated times with combined TNF [50ng/ml], emricasan [2.5µM], SM [1µM] and MK2i [10µM] (TESM). Representative Western blots are shown. **f,** $KRAS^{G12D}$ and $KRAS^{WT}$ MEFs from a treated with SM, birinapant [1 µM], emricasan [2.5 µM], nec1s [10 µM], TNF [50ng/ml] (n=3). Cell death was analysed by DRAQ7 fluorescence with normalization to cell confluence using the IncuCyte S3 bioimaging platform. **g,** WT MEFs were treated with control or IFNß [100ng/ml] for 48h followed by the indicated treatment combination for 24h TNF [50ng/ml], SM [1µM], emricasan [2.5µM], RIPK3i [3.3µM] (n=4). Number of dead cells was analysed by DRAQ7 fluorescence using the IncuCyte S3 bioimaging platform. **h,** $KRAS^{WT}$ or $KRAS^{G12D}$ MEFs (Rasless MEFs constitutively reconstituted) were treated with siMock or siMlkl-target siRNA pools for 48h (n=3). RNA was extracted and qPCR was used to analyse the expression of the indicated genes. **i,** LSL-$KRAS^{G12D}$-$C8^{fl/fl}$ MEFs were seeded with or without 4OHT [1µM] 4 days prior, then treated as indicated (TNF [50ng/ml], emricasan [2.5µM], SM [1µM], nec1s [10 µM]) for 24h. Dead cells (PI^{+} cells) were quantified by flow cytometry (n=3). **j,** The human cancer cell lines Bxpc3, Panc1, Panc89 were treated with control, human Interferon ß [1000U/ml] or human Interferon γ [1000U/ml] for 24h. The indicated proteins were detected by Western blotting. **k,** Ras84 score which was calculated as the median of the ranks of the 84 genes associated with RAS oncogenic activity for the indicated human PDAC cell lines. **l,** Heatmap representation of the expression of necroptosis relate genes in human PDAC cell lines, where responders respond to necroptosis inducing combination with cell death more than 30% and non-responders have less than 20% of cell death. Data are means +/- SEM. Unpaired t-test on lognorm-transformed data (a), multiple

unpaired t-test (c, h, i), multiple unpaired t-test (d) and two-way ANOVA (g) were used. Source data are provided as a Source Data file.

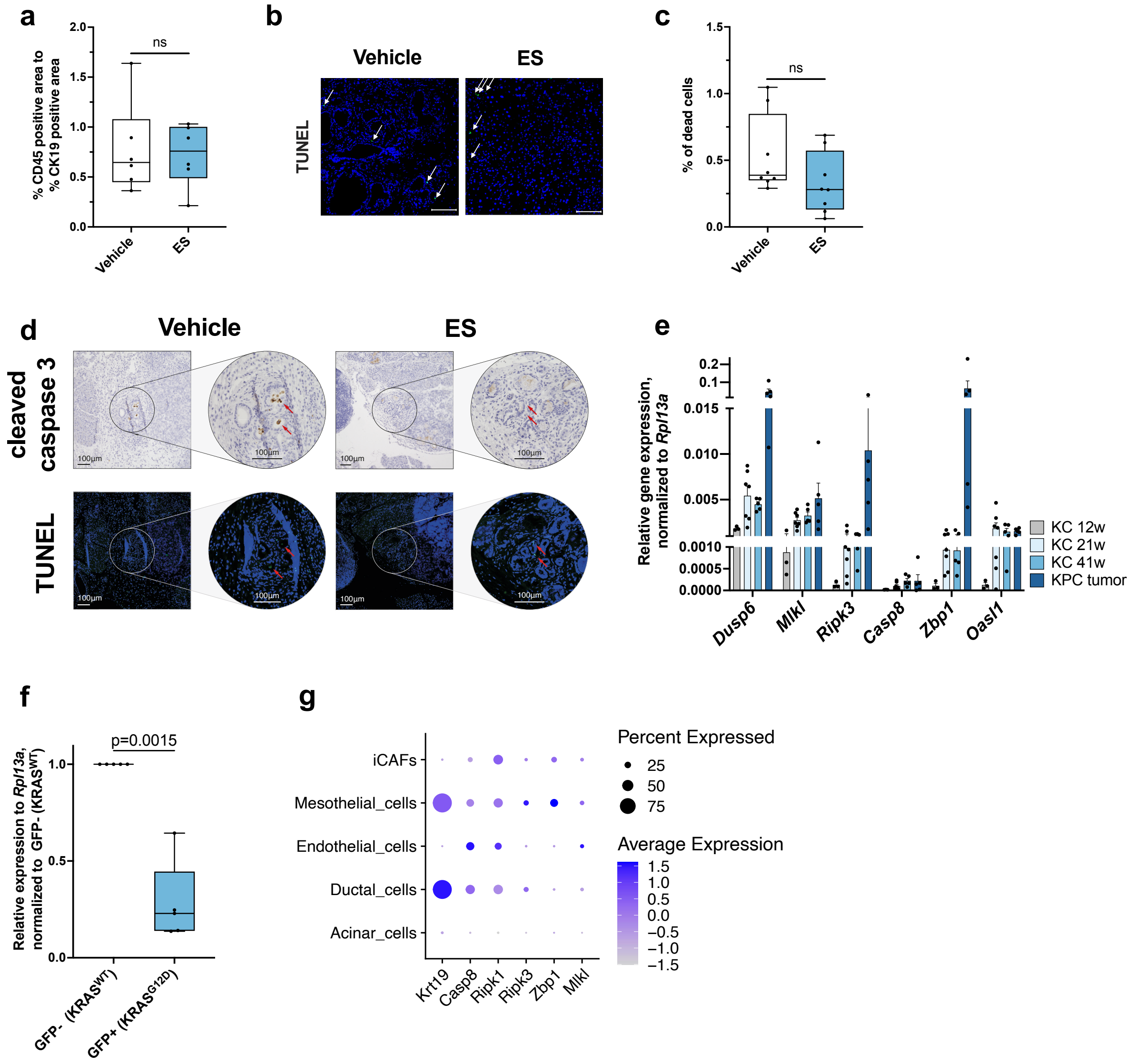

Supplementary Figure 7. Detection of cleaved caspase 3-negative cell death upon treatment.

a-d, 5-months-old KC-mice were treated i. p. with vehicle (PBS with 40% PEG-4000, 0.4% DMSO) or Emricasan (E) [2.5 mg/kg] / Birinapant (smac mimetic; S) [5 mg/kg], in (a-c) twice a week for 4 consecutive weeks, in (d) for 1.5 week. (**a-c**) Four weeks after the last treatment, mice were sacrificed, pancreata excised and tissues analysed with the indicated stainings. (**a**) KC- C8$^{wt/wt}$ treated with vehicle (n=6) or emricasan and smac mimetic (n=6) sections were stained for CD45 and CK19 using immunohistochemistry and quantified using QuPath. Boxplot centre line, mean; box limits, upper and lower quartile; whiskers min. to max. (**b, c**) Mice from a were stained by TUNEL assay (n=8 for each). (**b**) Representative images are shown. Size bars represent 100 µm. (**c**) TUNEL positive cells were quantified and normalised

to overall number of cells (DAPI^{+} cells) with the BZ-X800 microscope analysis software (Keyence). (**d**) Adjacent sections were stained for cleaved caspase 3 and TUNEL assay, representative images from the same area are shown. **e,** Pancreata from 12 (n=3), 21 (n=7), 41 (n=5) weeks-old KC-C8$^{wt/wt}$ and end point of KPC (n=5) mice were isolated and RNA extracted. The indicated genes were detected within bulk RNA using qPCR. **f**, Cells the analysis were sorted like in Figure 1c. Relative expression of *Zbp1* normalised to ribosomal housekeeping gene *Rpl13a* from adjacent WT pancreas (tdTomato^{+}, n=5) and KRAS^{G12D}-expressing PanINs (GFP^{+}, n=5) from 5-months-old KC mice. **g**, Cell type-specific expression of the indicated necroptosis genes is shown as log1p-transformed scaled average gene expression (scale factor 10000) within KC-C8$^{wt/wt}$ (WT) single cells. Boxplot centre line, mean; box limits, upper and lower quartile; whiskers min. to max. Data are means +/- SEM. Unpaired *t* test (a, c, f) and two-way ANOVA (e). Source data are provided as a Source Data file.

Supplementary Table 1. Clinical characteristics of patient-derived organoids (PDOs) used in this study.

PDO#	Organoid	KRAS status (Sanger-Seq)	Source	Gender	Age at Diagnosis	Staging	Grading
1	B265	WT		female			
2	B339	G12D	Surgery	male	60-70	pT3, pN2 (24/33, ECE+), Pn1, V1, CRM	G2
3	B379	G12D	FNB	male	70-80	pT2, pN1(1/40), Pn1, L1, V1, G2, R1, CRM+	G2

Supplementary Figure 3c uncropped blots. Lanes 5-8 correspond to those shown in Supplementary Figure 3c. Lanes 1-4 are not shown in the figure.

STAT1

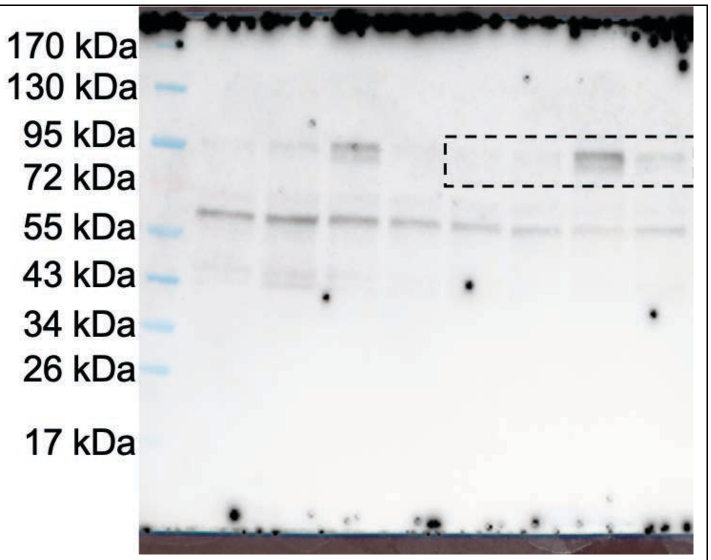

CASP8	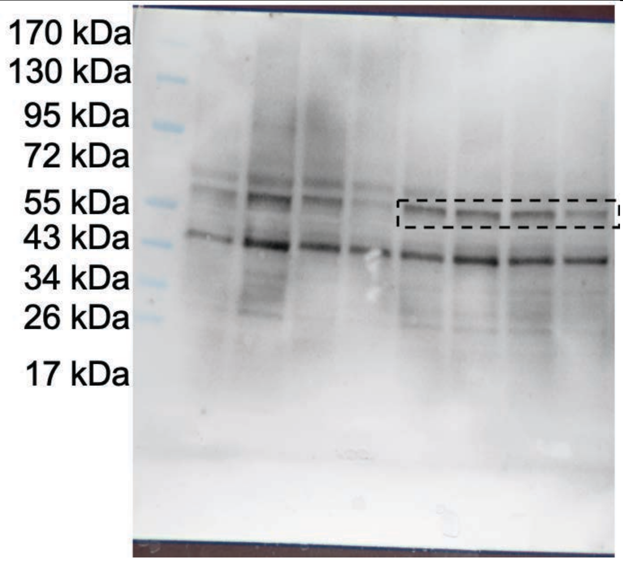 170 kDa 130 kDa 95 kDa 72 kDa 55 kDa 43 kDa 34 kDa 26 kDa 17 kDa
RIPK1	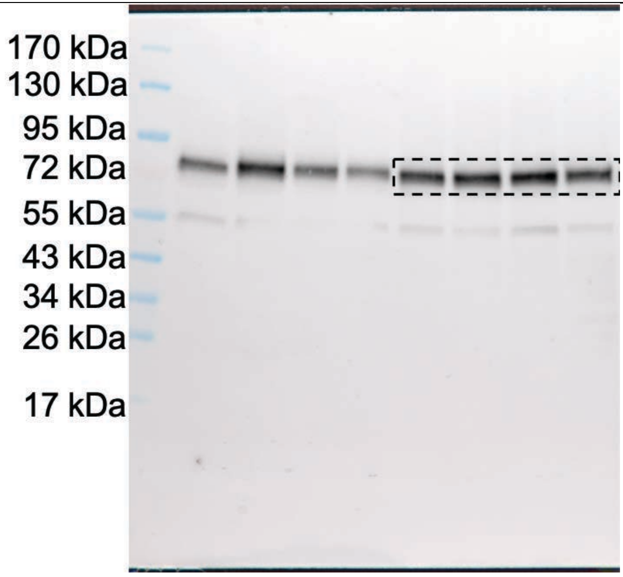 170 kDa 130 kDa 95 kDa 72 kDa 55 kDa 43 kDa 34 kDa 26 kDa 17 kDa

<table>
<tr><td>RIPK3</td><td>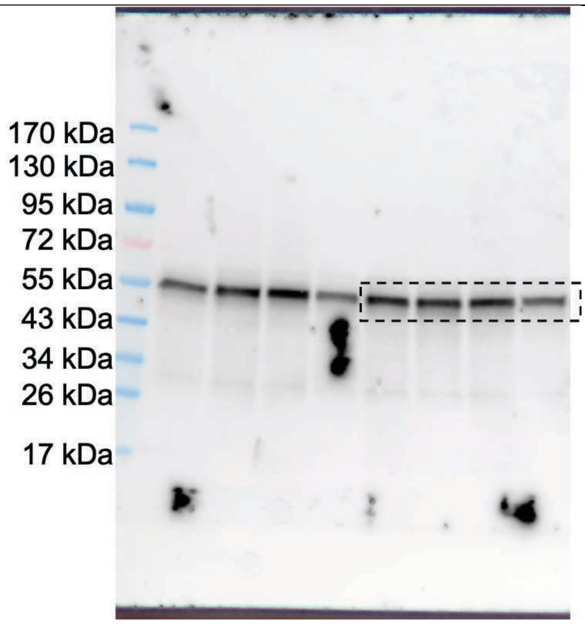
</td></tr>
<tr><td>MLKL</td><td>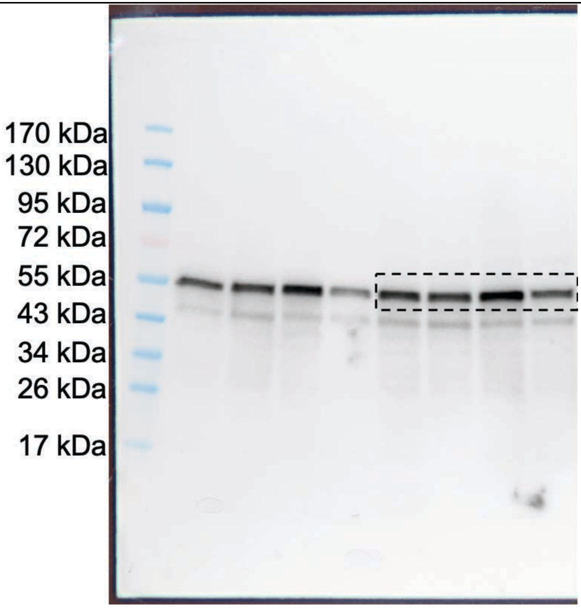
</td></tr>
<tr><td>β-Actin (membrane 1)</td><td>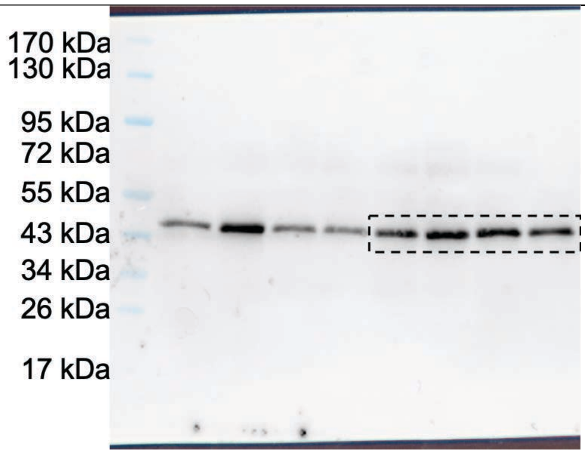
</td></tr>
</table>

β-Actin (membrane 2)	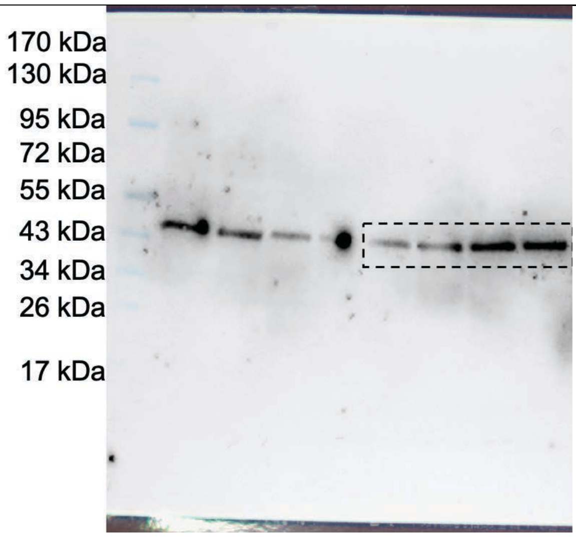

Supplementary Figure 4c uncropped blots. Lanes correspond to those shown in Supplementary Figure 4c. Western blots detected by X-ray film; molecular weight markers visible on membrane but not on film. Membrane was cut prior to primary antibody incubation.

MLKL	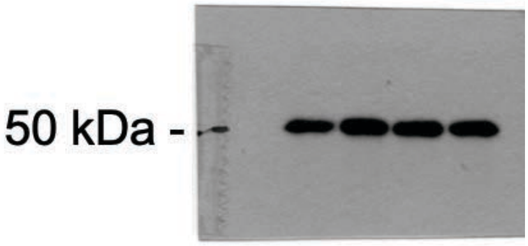
p-STING	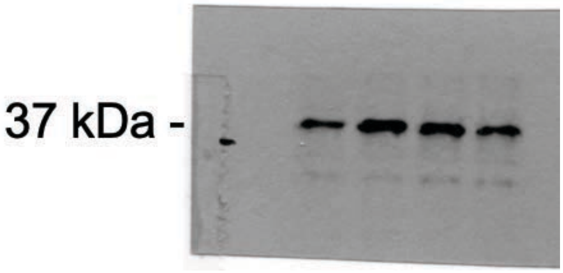
p-H2A.X	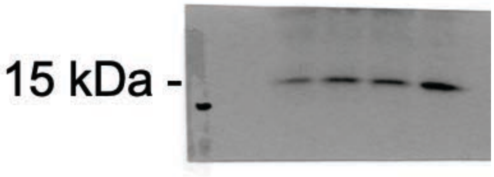

p-ERK1/2	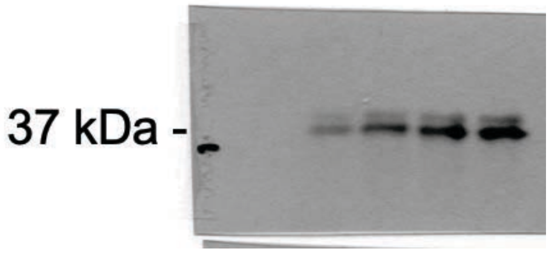
GAPDH	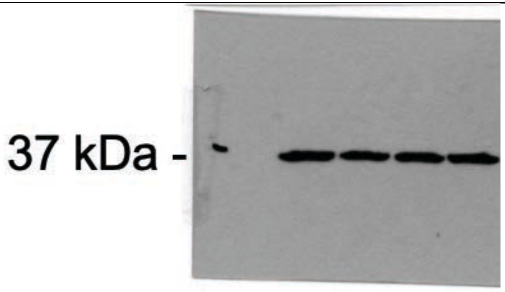

Supplementary Figure 4d uncropped blots. Lanes correspond to those shown in Supplementary Figure 4d.

STING	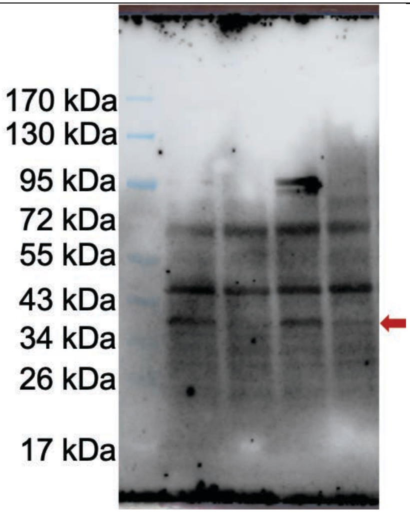

p-STAT1	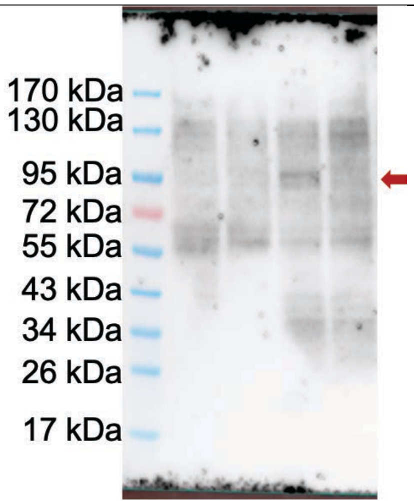
STAT1	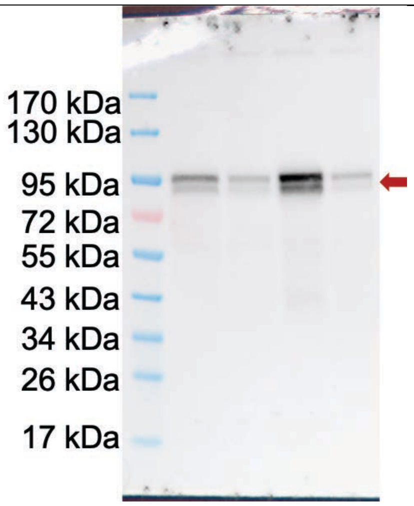
ZBP1	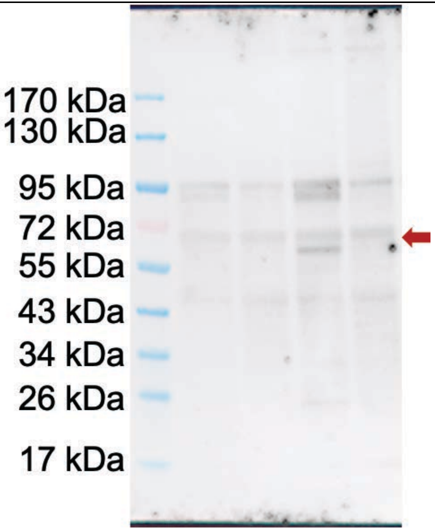

MLKL	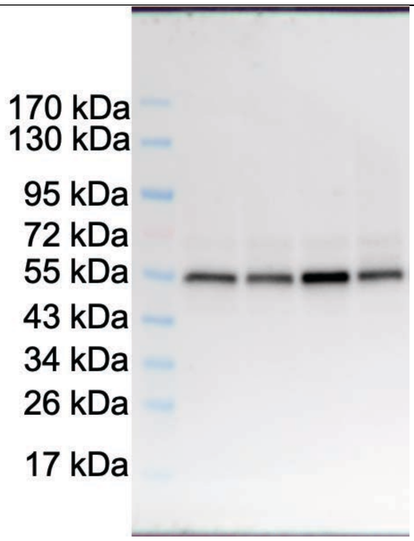
Vinculin	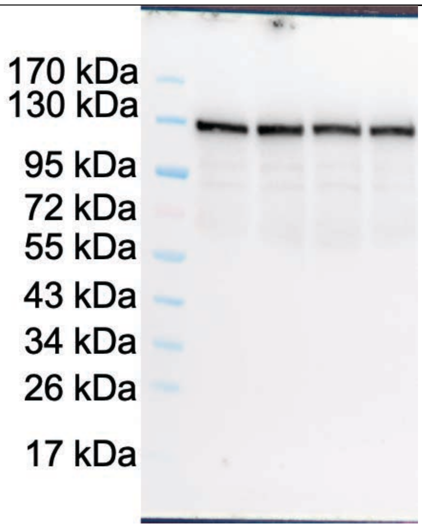

Supplementary Figure 5a uncropped blots. Lanes correspond to those shown in Supplementary Figure 5a. Membrane was cut before incubation with primary antibodies.

Jak1	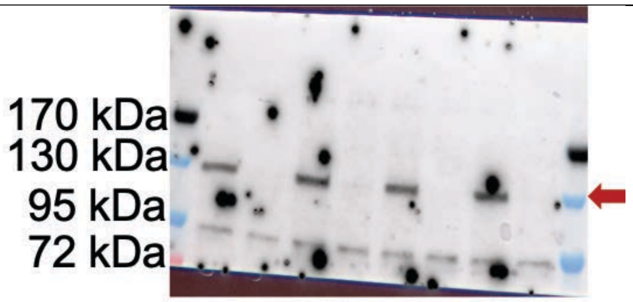

Jak2	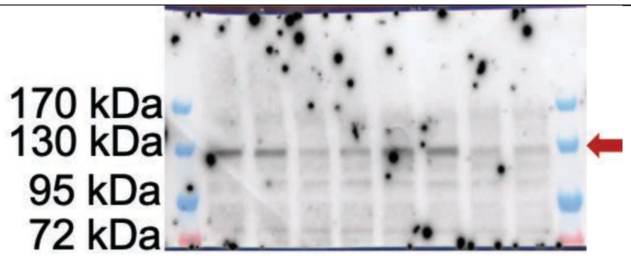
MLKL	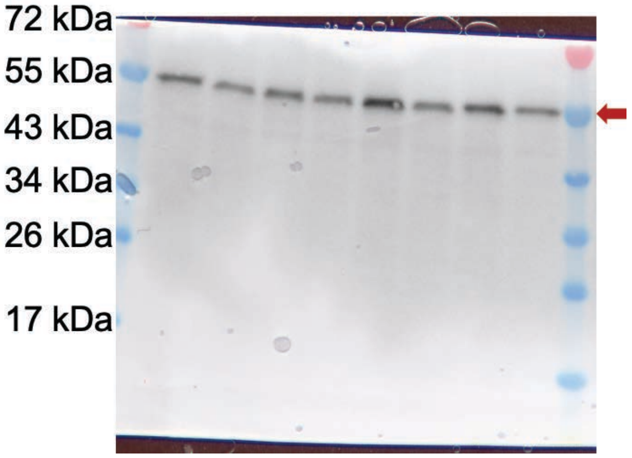
Vinculin (membrane 1)	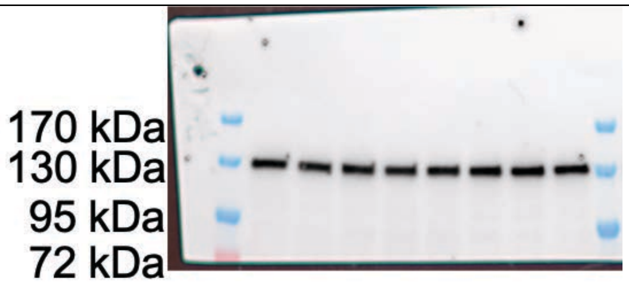
Vinculin (membrane 2)	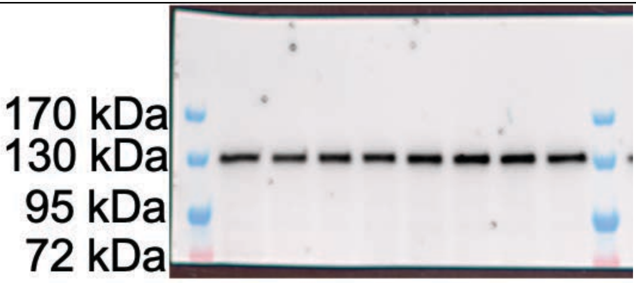

Supplementary Figure 5b uncropped blots. Lanes correspond to those shown in Supplementary Figure 5b.

p-STAT1	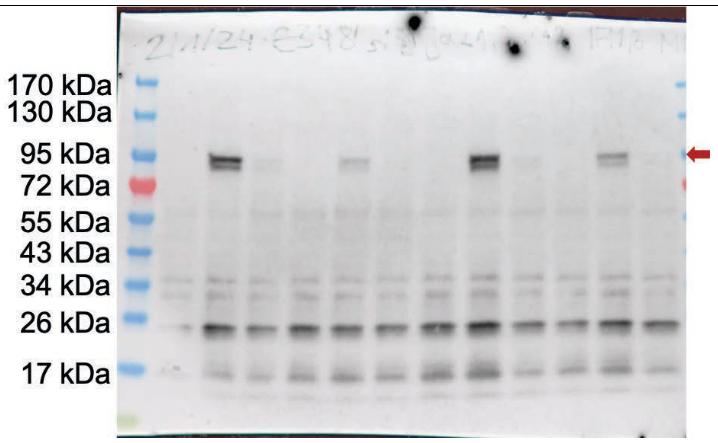
STAT1	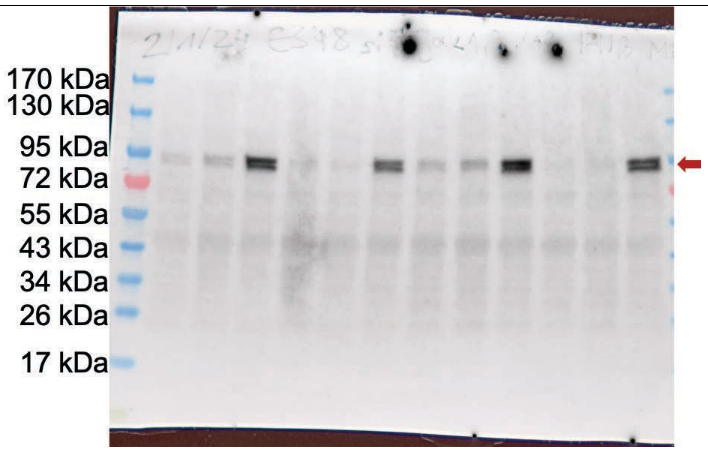
p-STAT2	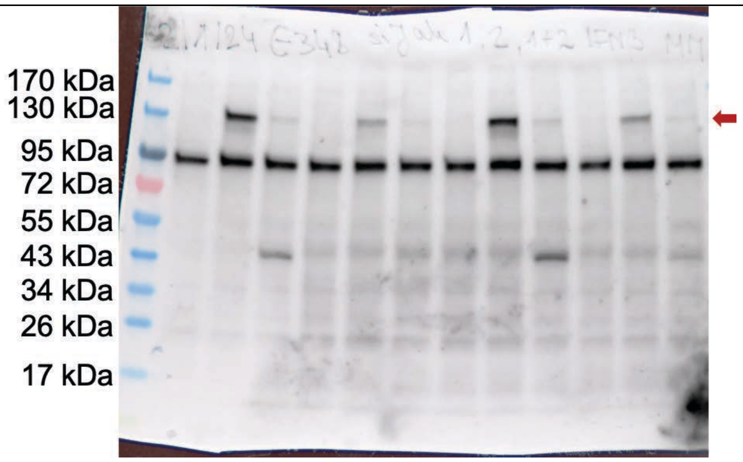

STAT2	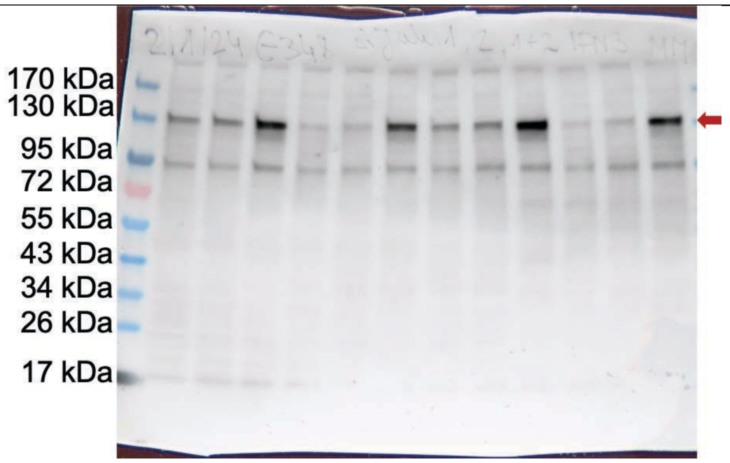
JAK1	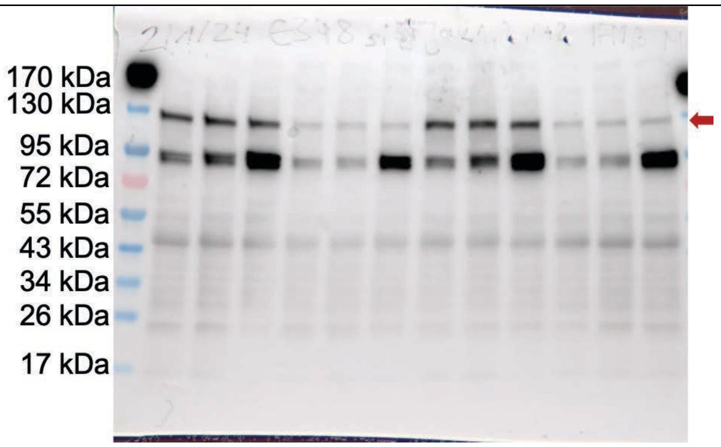
JAK2	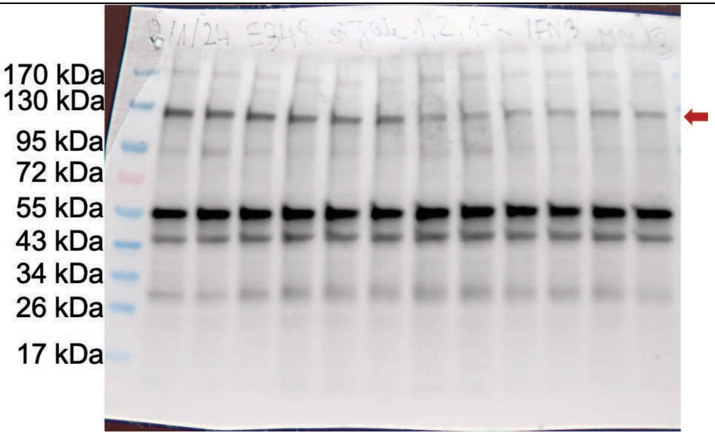

ZBP1	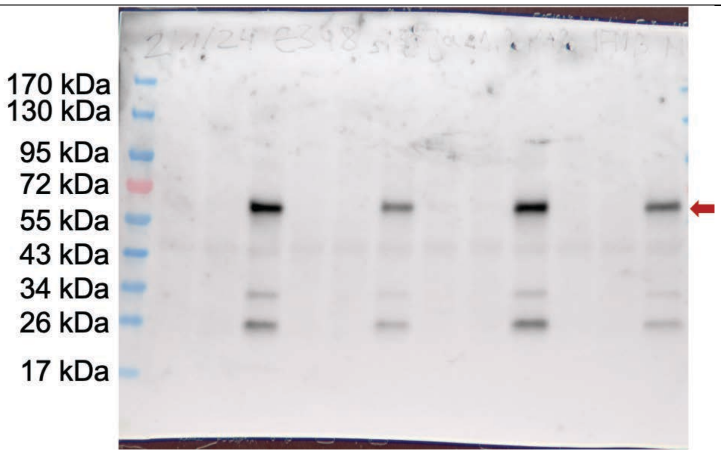
MLKL	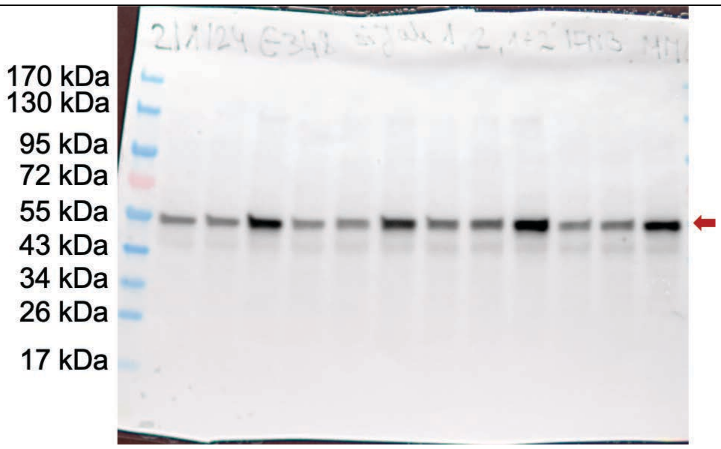
RIPK1	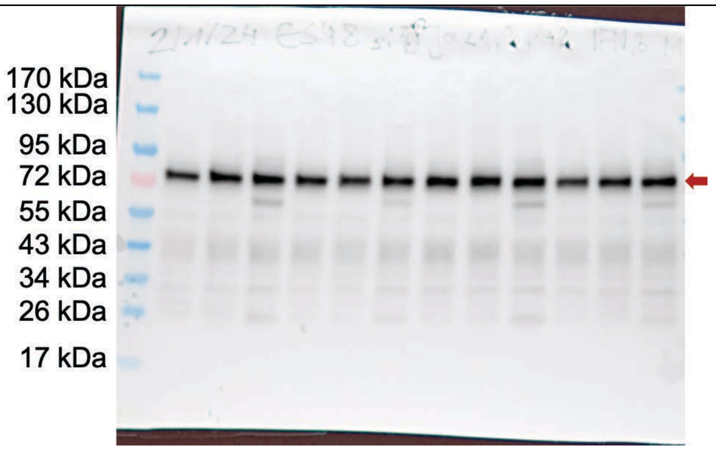

RIPK3	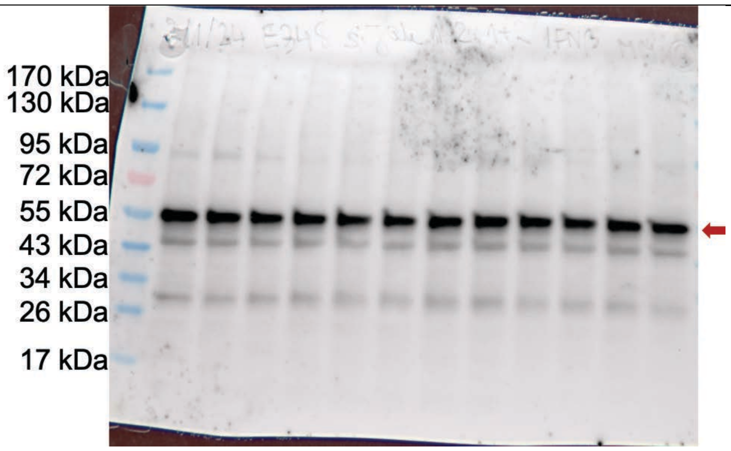
CASP8	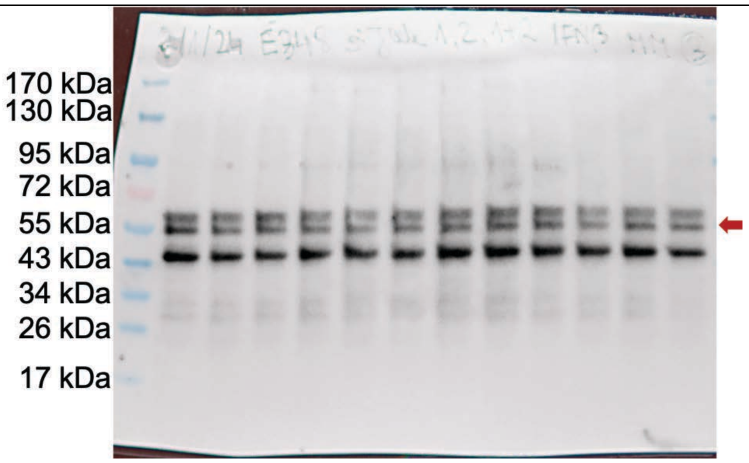
β-Actin (membrane 1)	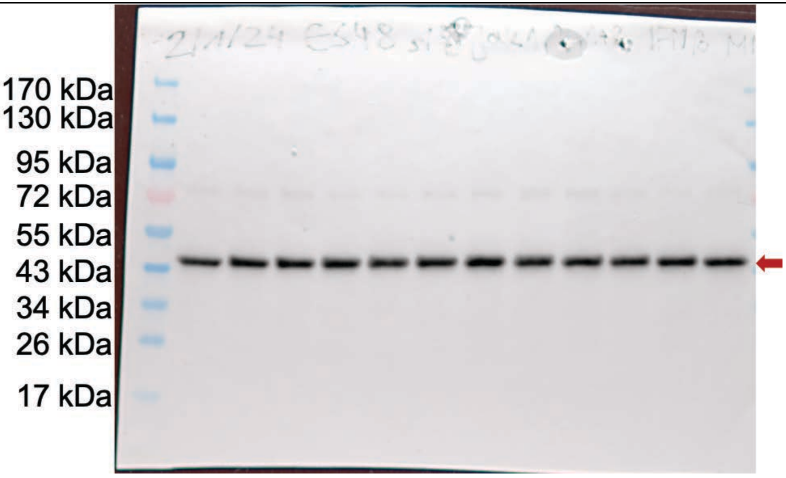

β-Actin (membrane 2)	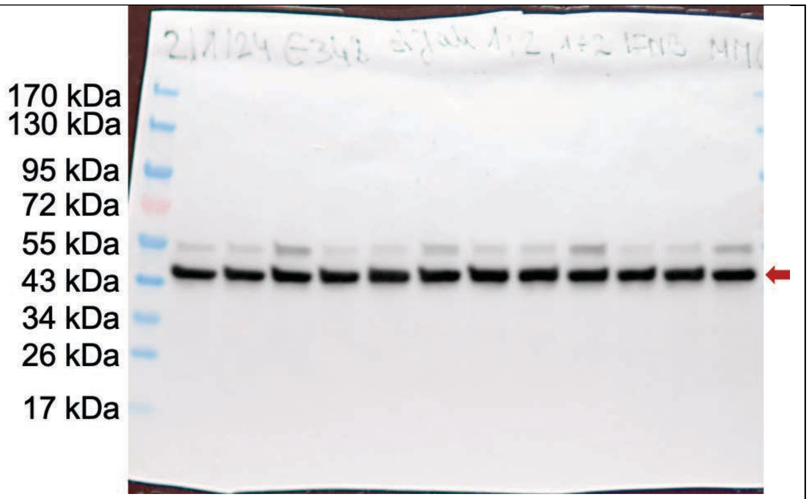
β-Actin (membrane 3)	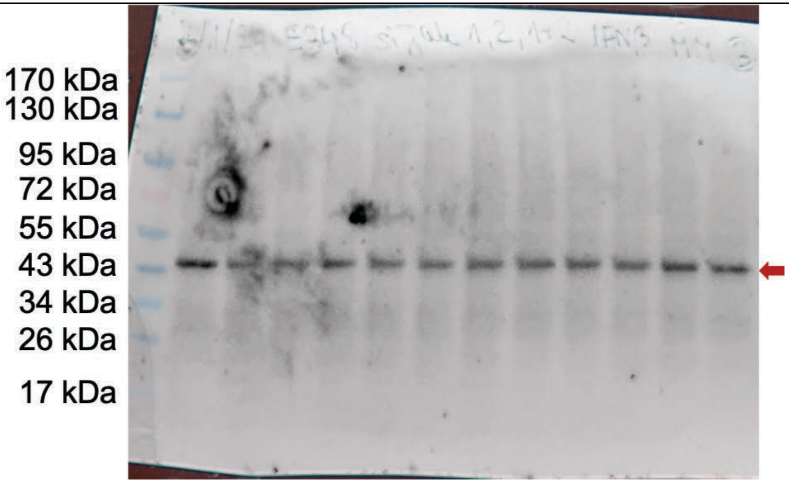

Supplementary Figure 6e uncropped blots. Lanes correspond to those shown in Supplementary Figure 6e. Membrane was cut before incubation with primary antibodies. Only the signal image is available for this blot.

p-MLKL	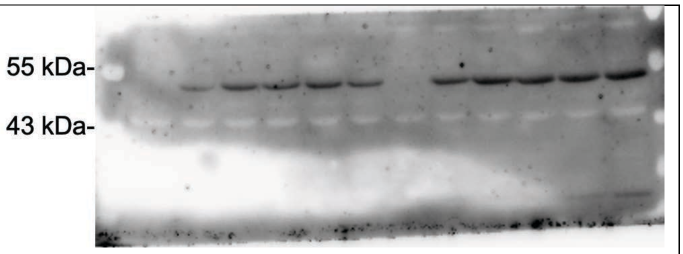

MLKL	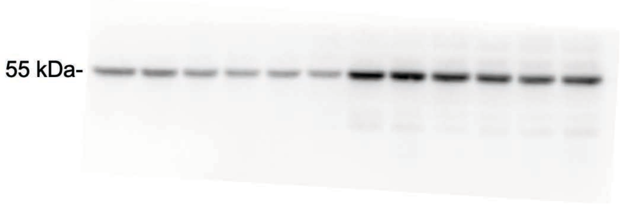
p-RIPK3	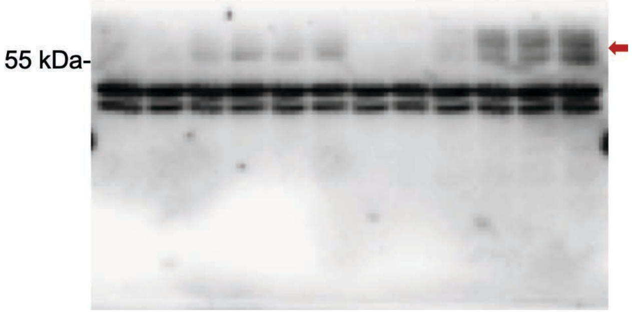
RIPK3	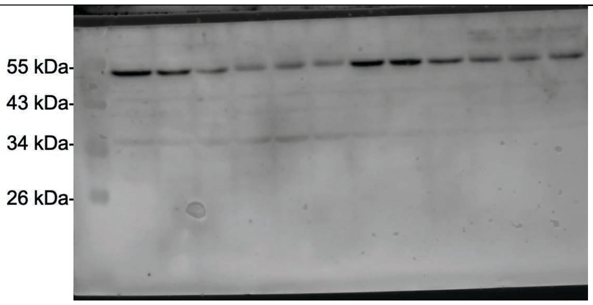
GAPDH	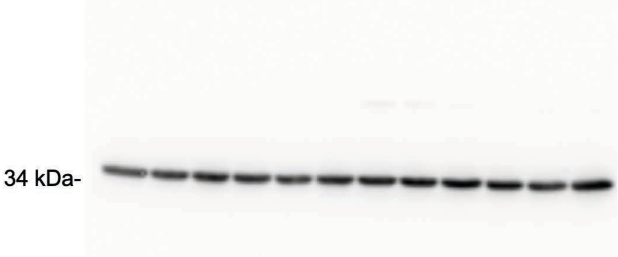

Supplementary Figure 6j uncropped blots. Lanes correspond to those shown in Supplementary Figure 6j.

CASP8	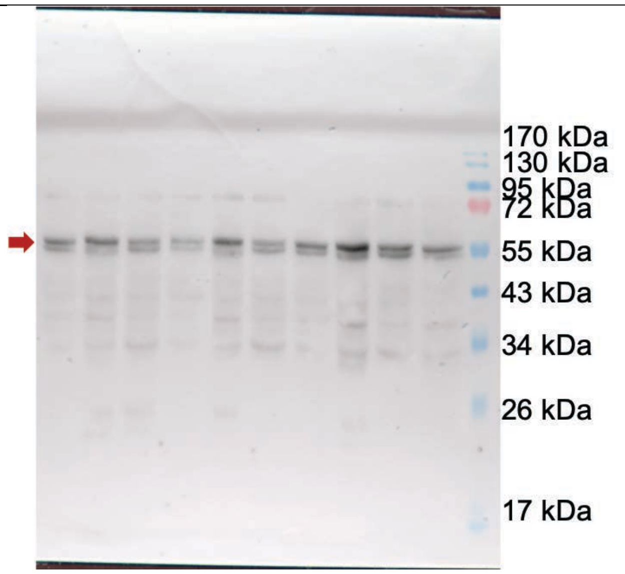170 kDa<br>130 kDa<br>95 kDa<br>72 kDa<br>55 kDa<br>43 kDa<br>34 kDa<br>26 kDa<br>17 kDa
RIPK1	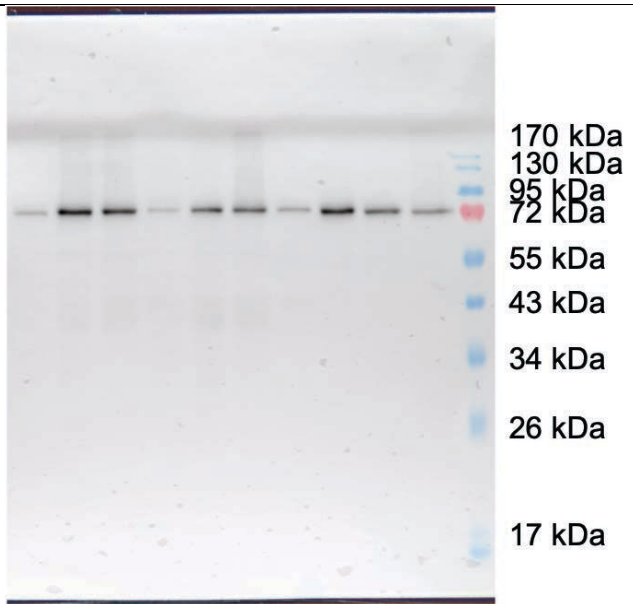170 kDa<br>130 kDa<br>95 kDa<br>72 kDa<br>55 kDa<br>43 kDa<br>34 kDa<br>26 kDa<br>17 kDa

RIPK3	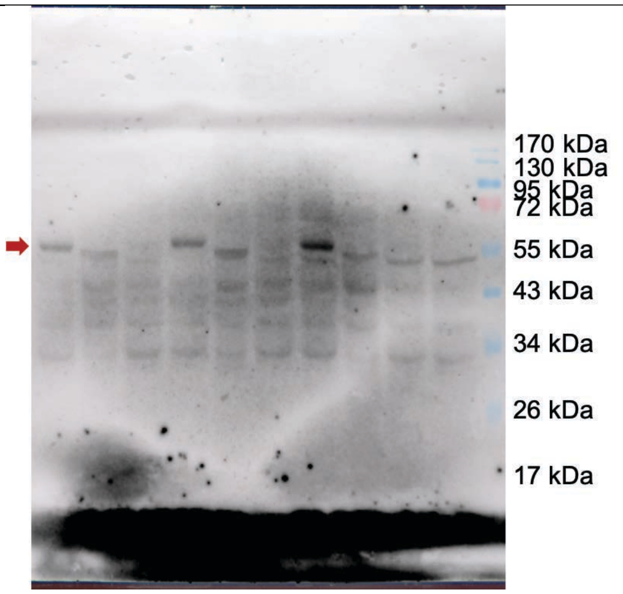
MLKL	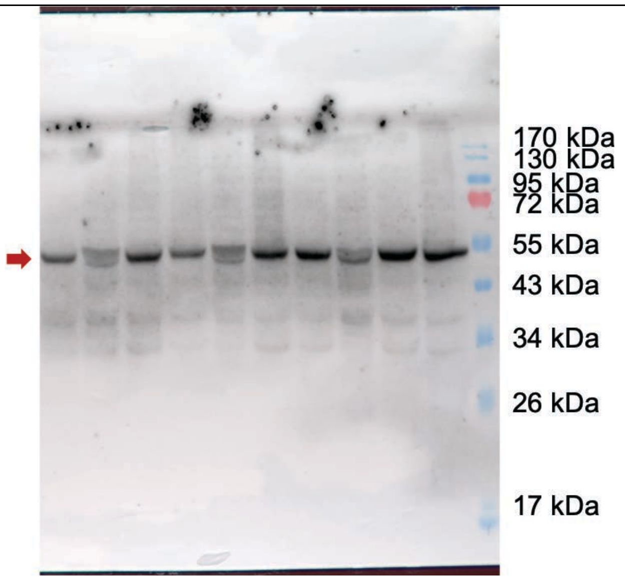
β-Actin	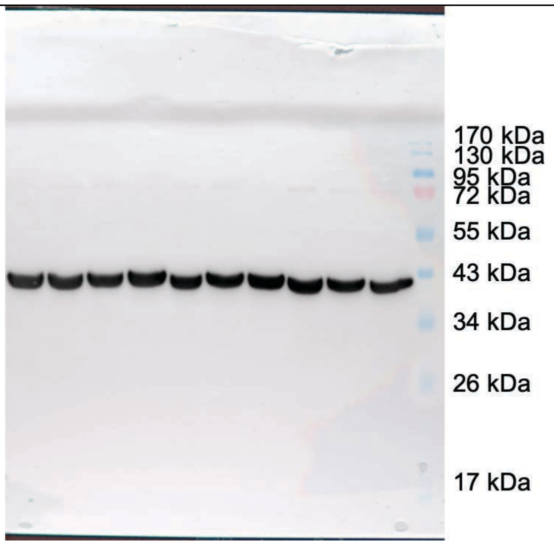
