# Supplementary material for: Oncogenic KRAS-driven type I interferon signalling primes pancreatic cancer for necroptosis
Source: Nat Commun. 2026 Jun 15;17:5288. doi: 10.1038/s41467-026-73189-8 (PMC13269921; doi:10.1038/s41467-026-73189-8)
Supplement: Supplementary file 2 — Description of Additional Supplementary Files [file 41467_2026_73189_MOESM2_ESM.pdf]

Title: Supplementary Data 1

Description: Marker genes for all cell types identified by scRNA-seq analysis of KC-C8wt/wt and KC-C8fl/fl pancreata (5-month-old mice). Cell types were assigned based on established pancreatic lineage markers. Differentially expressed genes were identified using the FindAllMarkers function (Seurat) with default parameters, returning only upregulated genes. Clustering resolution: 0.1. Columns: gene name (gene), unadjusted p-value (p_val), average log2 fold change (avg_log2FC), fraction of cells in the cluster expressing the gene (pct.1), fraction of all other cells expressing the gene (pct.2), Bonferroni-adjusted p-value (p_val_adj), cell type cluster.

Title: Supplementary Data 2

Description: Marker genes for immune cell subtypes identified by re-clustering of CD45+ cells from KC-C8wt/wt and KC-C8fl/fl pancreata. Clustering resolution: 0.2. Columns as in Sheet 1.

Title: Supplementary Data 3

Description: Oligonucleotide primer sequences used for quantitative real-time PCR (qPCR) in this study. Columns: primer name, sequence.
