# Supplementary material for: Oncogenic KRAS-driven type I interferon signalling primes pancreatic cancer for necroptosis
Source: Nat Commun. 2026 Jun 15;17:5288. doi: 10.1038/s41467-026-73189-8 (PMC13269921; doi:10.1038/s41467-026-73189-8)
Supplement: Supplementary file 4 — Reporting Summary [file 41467_2026_73189_MOESM4_ESM.pdf]

nature portfolio

Corresponding author(s): Silvia von Karstedt
Last updated by author(s): 17.04.2026

Reporting Summary

Nature Portfolio wishes to improve the reproducibility of the work that we publish. This form provides structure for consistency and transparency in reporting. For further information on Nature Portfolio policies, see our Editorial Policies and the Editorial Policy Checklist.

Statistics

For all statistical analyses, confirm that the following items are present in the figure legend, table legend, main text, or Methods section.

n/a	Confirmed	
☐	☒	The exact sample size (*n*) for each experimental group/condition, given as a discrete number and unit of measurement
☐	☒	A statement on whether measurements were taken from distinct samples or whether the same sample was measured repeatedly
☐	☒	The statistical test(s) used AND whether they are one- or two-sided<br>*Only common tests should be described solely by name; describe more complex techniques in the Methods section.*
☒	☐	A description of all covariates tested
☐	☒	A description of any assumptions or corrections, such as tests of normality and adjustment for multiple comparisons
☐	☒	A full description of the statistical parameters including central tendency (e.g. means) or other basic estimates (e.g. regression coefficient) AND variation (e.g. standard deviation) or associated estimates of uncertainty (e.g. confidence intervals)
☐	☒	For null hypothesis testing, the test statistic (e.g. *F*, *t*, *r*) with confidence intervals, effect sizes, degrees of freedom and *P* value noted<br>*Give P values as exact values whenever suitable.*
☒	☐	For Bayesian analysis, information on the choice of priors and Markov chain Monte Carlo settings
☒	☐	For hierarchical and complex designs, identification of the appropriate level for tests and full reporting of outcomes
☐	☒	Estimates of effect sizes (e.g. Cohen's *d*, Pearson's *r*), indicating how they were calculated

Our web collection on statistics for biologists contains articles on many of the points above.

Software and code

Policy information about availability of computer code

Data collection	FACS data were acquired using BD FACS Diva version 8.0, Live- cell images were acquired with IncuCyte 2022B (Sartorius)
Data analysis	No custom code was used for the study. All published code used for data analysis is described in detail and referenced in the methods section.<br><br>-RNA-sequencing data of patient cohorts were processed with STAR aligner and analyzed with RSEM<br>-Bioinformatics analysis of single-cell RNA sequencing data was done using the PARSE scRNAseq pipeline v.1.1.1. Data analysis was performed using Seurat (v4.4.0)<br>- RNA -seq analysis was conducted using rnaseq pipeline from the nf-core suite (v3.7). STAR aligner (v2.7.10a), DESeq2 (v1.36.0 and fdrtool (v1.2.17) were used for futher analysis as described in the methods part. Heta maps were generated using R.<br>-Tissue stainings and tumor spheres were analysed using Omero.web 5.25.0 and BZ-X800 Analyzer software.<br>-qPCR data and ELISA data were analysed using Excel. Graphs and statistical analysis of the data were performed in GraphPad prism v9.5.0<br>-Incucyte data were analyzed using the Software IncuCyte 2022B (Sartorius).<br>-FACS data were analyzed using FlowJo version 10.8.2

For manuscripts utilizing custom algorithms or software that are central to the research but not yet described in published literature, software must be made available to editors and reviewers. We strongly encourage code deposition in a community repository (e.g. GitHub). See the Nature Portfolio guidelines for submitting code & software for further information.

Data

Policy information about availability of data

All manuscripts must include a data availability statement. This statement should provide the following information, where applicable:

- Accession codes, unique identifiers, or web links for publicly available datasets
- A description of any restrictions on data availability
- For clinical datasets or third party data, please ensure that the statement adheres to our policy

All RNA-seq and scRNA-seq data generated in this study have been deposited in the NCBI Sequence Read Archive (SRA) under accession codes PRJNA975357 and PRJNA975358. Source data for all graphs are provided in the Source Data file. All bioinformatic analyses were performed using publicly available software and packages as described in the Methods. The necroptosis score was calculated as described in the Methods and the underlying data are provided in the Source Data file. The remaining data are available within the Article, Supplementary Information or Source Data file. No data are available under restricted access.

Research involving human participants, their data, or biological material

Policy information about studies with human participants or human data. See also policy information about sex, gender (identity/presentation), and sexual orientation and race, ethnicity and racism.

Reporting on sex and gender	Human patient–derived organoids (PDO) were obtained from different patient groups to assess the generalisability of treatment efficacy. Organoids were derived from three patients, including two males and one female. All available clinical data on PDOs are in Supplementary Table 4.
Reporting on race, ethnicity, or other socially relevant groupings	This information is not available for the PDOs .
Population characteristics	n/a
Recruitment	n/a
Ethics oversight	The primary human PDAC cellular models were established and analyzed in accordance with the Declaration of Helsinki; were approved by the local ethical committee of TUM, Klinikum rechts der Isar and LMU, Klinikum der Universität München (projects 207/15, 1946/07, 330/19S, 80/17S, 5542/12, and 17-648); and written informed consent from the patients for research was obtained prior to the investigation.

Note that full information on the approval of the study protocol must also be provided in the manuscript.

Field-specific reporting

Please select the one below that is the best fit for your research. If you are not sure, read the appropriate sections before making your selection.

☒ Life sciences ☐ Behavioural & social sciences ☐ Ecological, evolutionary & environmental sciences

For a reference copy of the document with all sections, see nature.com/documents/nr-reporting-summary-flat.pdf

Life sciences study design

All studies must disclose on these points even when the disclosure is negative.

Sample size	Sample sizes and numbers of repeats are indicated in the figure legends, Methods, and the text. Sample sizes were not determined a priori but were chosen based on experience, reproducibility, and animal licence allowances and limitations.
Data exclusions	no data were excluded
Replication	All cell-based experiments were performed with triplicate or quadruplicate measurements within each individual experiment, and means were calculated accordingly. These experiments were repeated independently at least three times to obtain independent means. Figures show means ± SEM calculated from these independent experiments.<br>All in vivo mouse experiments were performed in two or three independent cohorts, each including at least three independent biological samples. Data from all cohorts were pooled for presentation in the figures, and independent cohorts reproducibly yielded the same results.
Randomization	Mice were allocated to experimental groups to obtain an approximately equal number of males and females. Where applicable, littermates were allocated to different treatment groups.
Blinding	Investigators were only blinded while performing histological scoring. For the rest, the blinding was not possible as mouse genotypes were screened to have necessary selection of mice.

Reporting for specific materials, systems and methods

We require information from authors about some types of materials, experimental systems and methods used in many studies. Here, indicate whether each material, system or method listed is relevant to your study. If you are not sure if a list item applies to your research, read the appropriate section before selecting a response.

Materials & experimental systems

n/a	Involved in the study
☐	☒ Antibodies
☐	☒ Eukaryotic cell lines
☒	☐ Palaeontology and archaeology
☐	☒ Animals and other organisms
☒	☐ Clinical data
☒	☐ Dual use research of concern
☒	☐ Plants

Methods

n/a	Involved in the study
☒	☐ ChIP-seq
☐	☒ Flow cytometry
☒	☐ MRI-based neuroimaging

Antibodies

Antibodies used	The following antibodies for Western Blots were used: AKT (Cell Signaling, 4691 1:1000), Caspase 8 (AdipoGen, AG-20B-0057-C050 1:1000), Caspase 8 (Enzo Life Sciences, ALX-804-447-C100 1:1000), ERK (Cell Signaling, 9102 1:1000), GAPDH (Cell Signaling, 97166 1:2000), IKBα (Santa Cruz, sc-1643 1:1000), IRF3 (Abcam, ab68481 1:1000), IRF7 (Cell Signaling, 72073 1:1000), IRF9 (Cell Signaling, 28845 1:1000), JAK1 (Cell Signaling, 3344 1:1000), JAK2 (Cell Signaling, 3230 1:1000), MK2 (Cell Signaling, 3042 1:1000), MLKL (Cell Signaling, 14993 1:1000), MLKL (Millipore, MABC604 1:1000), p-AKT (Ser473) (Cell Signaling, 4060 1:1000), p-ERK (Thr202/Tyr204) (Cell Signaling, 4307 1:1000), p-IKBα (Cell Signaling, 9246 1:1000), p-MK2 (Thr334) (Cell Signaling, 3007 1:1000), p-MLKL (Ser345) (Cell Signaling, 37333 1:1000), p-MLKL (Ser358) (Cell Signaling, 91689 1:1000), p-p38 (Cell Signaling, 4511 1:1000), p-RIPK1 (Ser166) (Cell Signaling, 65746 1:1000), p-RIPK3 (Thr231/Ser232) (Cell Signaling, 91702 1:1000), p-STAT1 (Tyr701) (Cell Signaling, 9167 1:500), p-STAT2 (Tyr689) (Sigma-Aldrich, 07-224 1:500), p-STAT3 (Tyr705) (Cell Signaling, 9145 1:500), p38 (Cell Signaling, 9212 1:1000), RIPK1 (Cell Signaling, 3493 1:1000), RIPK3 (Cell Signaling, 15828 1:1000), RIPK3 (Enzo Life Sciences, ADI-905-242 1:1000), ß-Actin (Sigma, A1978, 1:10,000), STAT1 (Cell Signaling, 14995 1:1000), STAT2 (Sigma-Aldrich, 07-140 1:1000), STAT3 (Cell Signaling, 9139 1:1000), STING (Cell Signaling, 13647 1:1000), pSTING (Cell Signaling, 19781 1:1000), Vinculin (Cell Signaling, 13901 1:2000), ZBP1 (Adipogen, A42342106 1:1000)); HRP-conjugated secondary antibodies: goat-anti-mouse-HRP (Linaris GmBH, 20400, 1:10,000), goat-anti-rabbit-HRP (Linaris GmBH, 20402, 1:10,000), goat-anti-rat-HRP (Sigma, A9037, 1:10,000). The following antibodies were used for tissue stainings: Cytokeratin 19 (1:100, AHP1846, Bio rad antibodies (discontinued) and TROMA-III from Developmental Studies Hybridoma Bank Iowa University), CD45 (1:100, 14-0451-82, Invitrogen), Caspase 8 (1:200, ALX-804-447-C100, Enzo), cleaved caspase 3 (1:500, 9661, Cell Signaling), p-STAT1 (1:50, 9167, Cell Signaling). The following antibodies were used for micronuclei microscopy: DAPI (D1306, InvitrogenTM), Anti-DNA Antibody (CBL186, Sigma), ProLong Gold antifade (P36934, InvitrogenTM). Fluorescence-activated cell sorting (FACS) antibodies: Fc block (CD16/32, clone 93, 101301, biolegend, 1:50), CD45-BV421 (30.F11, 1:1000, 103133, biolegend), CD45-FITC (30.F11, 1:1000, 103107, biolegend), NK1.1- BV421 (PK136, 1:1000, 156537, biolegend), CD4-V450 (RM4-5, 1:1000, 560470, BD Horizon), CD11b-PE (M1/70, 1:1000, 12-0112-81, ebioscience), CD8a-PE (53-6.7, 1:1000, 100707, biolegend), CD19- PE (1D3, 1:1000, 12-0193-82, ebioscience), CD11c-BV421 (N418, 1:1000, 117329, biolegend), Ly-6G/Ly-6C-FITC (RB6-BC5, 1:1000, 108435, ebioscience), Gr1-BV-711 (RB6-8C5, 1:1000, 108443, biolegend), CD14-FITC (Sa14-2, 1:1000, 123307, biolegend), CCR3-FITC (J073E5, 1:1000, 144509, biolegend), CD206-BV421 (MMR, C068C2, 1:50, 141717, biolegend), Rat IgG2a, κ-isotype Ctrl -BV-421 (RTK-2758, 1:50, 400501, biolegend).
Validation	Antibodies were validated by the supplier or siRNA-mediated knockdown.

Eukaryotic cell lines

Policy information about cell lines and Sex and Gender in Research

Cell line source(s)	LSL-KRASG12D inducible MEFs were generated as described previously46. “Rasless” MEFs reconstituted with either WT KRAS4B or KRASG12D (RPZ25854, RPZ26198) were generated and kindly provided by the RAS Initiative at the Frederick National Laboratory for Cancer Research (FNLCR), US. Rasless MEFs were grown in Dulbecco’s modified Eagle’s (DMEM) + GlutaMAX™ medium (Gibco) with 4 μg/ml of blasticidin. Freshly isolated LSL-KRASG12D inducible MEFs and IFNAR1-/- MEFs were cultured in DMEM (Gibco) supplied with 1% L-Glutamine (Sigma) and 1% Sodium Pyruvate (Sigma). Control, STAT1, STAT2 and IRF9-deficient MEFs were previously published (Platanitis, 2019) and kindly provided by Thomas Decker. Mouse PDAC cell lines (dox-KRASG12D mPDAC) derived from KC mice and engineered to express a doxycycline-inducible additional KRASG12D allele were kindly provided by Roland Rad (Mueller, 2018). Inducible human pancreatic duct epithelial cells (HPDE) pCW-KRASG12D were described previously in Müller, 2022 and cultured in 75% RPMI 1640/ medium in presence of 25% keratinocyte growth medium 2 (PromoCell) + 0,5μg Puromycin, BxPC-3 pCW-KRASG12D46 in RPMI 1640 GlutaMAX™ + 1% Sodium Pyruvate (Sigma) + 2,5 μg Puromycin. Human PDAC cell lines BxPC-3, A818-6, HPAF-II, Capan-1, AsPC1, PancTul, Colo 357, Capan-2, PANC-1, PANC-89, MIA PaCa-2, PT45 were cultured in RPMI 1640 GlutaMAX™ + 1% Sodium Pyruvate (Sigma). KC- organoids were isolated from KC-mice and cultured as described previously in Müller, 2022. All media were supplemented with 10% fetal calf serum (FCS) (Sigma Aldrich) and 1000 U/mL of both penicillin and streptomycin (Pen/Strep) (Sigma Aldrich). All cells were kept at 37°C with 5% CO2 and tested for mycoplasma at regular intervals (mycoplasma barcodes, Eurofins Genomics).
Authentication	All human PDAC cell lines were authenticated by STR profiling.

Mycoplasma contamination	All cells were kept at 37°C with 5% CO2 and tested for mycoplasma at regular intervals (mycoplasma barcodes, Eurofins Genomics). All cells were confirmed to be mycoplasma negative.
Commonly misidentified lines (See ICLAC register)	none of the cell lines used are commonly misidentified cell lines.

Animals and other research organisms

Policy information about studies involving animals; ARRIVE guidelines recommended for reporting animal research, and Sex and Gender in Research

Laboratory animals	Male and female mice were used in all experiments with balanced sex ratios maintained across experimental groups to avoid sex-related confounding effects. For scRNA-seq analysis, sex ratios were unintentionally unbalanced between compared groups due to sample availability at the time of collection; sex-specific genes (Xist, Ddx3y) were therefore excluded from the analysis to account for this. Sex-disaggregated numbers (male/female breakdown per group) are provided in the Source Data file for all in vivo experiments. LSL-KRASG12D-23 and LSL-Trp53R172H are on mixed 129/SvJae/C57Bl/6J background PDX-Cre mice on C57Bl/6J were purchased from the Jackson Laboratory. Casp8fl/fl mice on a C57BL/6N background were obtained under a material transfer agreement (MTA) from Stephen Hedrick. MLKL-/- mice on C57BL/6N were newly generated in the Pasparakis lab and described in Körner et al. , RIPK3-/- mice on C57BL/6N background were obtained from Genentech under an existing MTA as part of the Pasparakis lab 48. All mice were maintained on a 12-hour light/dark cycle at 20–22°C ambient temperature and 50–60% humidity, with water and food ad libitum. For four consecutive weeks, 5-months-old mice were injected i.p. 2 x per week either with vehicle (PBS with 40% PEG-4000, 0.4% DMSO) or emricasan [2.5 mg/kg] with birinapant [5 mg/kg]. KC mice were sacrificed four weeks after the last treatment and the pathologist was blinded to the group allocation while performing the progression analysis. 8-week-old KPC mice were treated as described above and kept until humane experimental endpoint. For all Kaplan-Meier experiments experimental endpoints were chosen based upon a scoring system quantifying animal well-being based upon weight loss, general condition, abnormal behaviour as common practice in German animal experimentation. Animals were euthanised once the cumulative score reached 20. Scoring was performed by a person blinded to the study.
Wild animals	n/a
Reporting on sex	male and female mice equally included in all studies and showed comparable results. For single cell analysis, animals of the same age were originally included yet due to poor quality three mice dropped out leaving 3 males in the KC-WT group and 2 females in the KC-C8 floxed group. Therefore, sex-determining transcripts were removed from the DEA analysis.
Field-collected samples	n/a
Ethics oversight	This study was conducted in compliance with all relevant ethical regulations. All animal experiments were approved by local government authorities (Landesamt für Verbraucherschutz und Ernährung, Nordrhein-Westfalen, LAVE, Germany, license numbers: 2017.A433; 2017.A477; 2022.A364) and were conducted in compliance with European, national and institutional guidelines on animal welfare at the University of Cologne, Germany. All people involved in animal experiments received prior training and have passed the additionally required personal licensing course (FELASA B). Mice were were closely monitored and sacrificed at the indicated experimental endpoint (time) or later at humane endpoint when reaching a score 10 (moderate burden) within a scale of 20 (severe burden) to minimize animal suffering as approved by local authorities. The maximum burden was not exceeded in any experiment. The primary human PDO models were established and analysed in accordance with the Declaration of Helsinki; were approved by the local ethical committee of Technical University Munich (TUM), Klinikum rechts der Isar and LMU, Klinikum der Universität München (projects 207/15, 1946/07, 330/19S, 80/17S, 5542/12, and 17-648); and written informed consent from the patients for research was obtained prior to the investigation. Publicly available TCGA and other cited RNA-sequencing datasets were used; prior ethics approval has been obtained for these studies, and no additional approval is required.

Note that full information on the approval of the study protocol must also be provided in the manuscript.

Plants

Seed stocks	n/a
Novel plant genotypes	n/a
Authentication	n/a

Flow Cytometry

Plots

Confirm that:

- [x] The axis labels state the marker and fluorochrome used (e.g. CD4-FITC).
- [x] The axis scales are clearly visible. Include numbers along axes only for bottom left plot of group (a 'group' is an analysis of identical markers).
- [x] All plots are contour plots with outliers or pseudocolor plots.
- [x] A numerical value for number of cells or percentage (with statistics) is provided.

Methodology

Sample preparation	Single cell suspensions of immune cells obtained from tumors were stained for live-dead cells, washed, incubated with Fc block and stained for surface markers. For intracellular staining, the eBioscience™ Foxp3/transcription factor staining buffer kit was used. In this case, after surface marker staining, cells were fixed and permeabilized, blocked with goat serum and stained intracellularly.
Instrument	FACS data were acquired on an LSR Fortessa (cat no. 647788, BS Bioscience), November 2013 model no. 647788E3
Software	Data were acquired using Diva software (BD Bioscience), analysis was performed using FlowJo version 10.8.2 or Diva (8.0)
Cell population abundance	10000-20000 cells were acquired to measure abundance of target cell population.
Gating strategy	Gating was based on non-stained/single-stained samples in order to distinguish between positive and negative populations

- [ ] Tick this box to confirm that a figure exemplifying the gating strategy is provided in the Supplementary Information.
