# Supplementary material for: Oncogenic KRAS-driven type I interferon signalling primes pancreatic cancer for necroptosis
Source: Nat Commun. 2026 Jun 15;17:5288. doi: 10.1038/s41467-026-73189-8 (PMC13269921; doi:10.1038/s41467-026-73189-8)
Supplement: Supplementary file 5 — Transparent Peer Review file [file 41467_2026_73189_MOESM5_ESM.pdf]

nature portfolio

Peer Review File

Oncogenic KRAS-Driven type I Interferon Signalling Primes Pancreatic Cancer for Necroptosis

Corresponding Author: Professor Silvia Von Karstedt

This file contains all reviewer reports in order by version, followed by all author rebuttals in order by version.

Version 1:

Reviewer comments:

Reviewer #1

(Remarks to the Author)
In this manuscript, Tishina and colleagues focused on the higher expression of Caspase-8, which initiates the apoptosis cascade, in KRAS-dependent PDAC and is associated with a rather poorer prognosis in patients. Utilizing a sophisticated murine PDAC model combined with deletion of Caspase-8 and/or MLKL, they demonstrated that Caspase-8 functions as a tumor suppressor by inhibiting MLKL-induced necroptosis. This provides compelling evidence that the suppression of necroptosis by Caspase-8 contributes to pancreatic cancer progression. In addition, they showed that the STING/TBK1 pathway and subsequent autocrine type I IFN signaling activated by oncogenic KRAS is crucial for inducing the molecules driving necroptosis, such as MLKL and ZBP1. While it is well-known that Caspase-8 inhibits necroptosis, this study shed light on the novel aspects of the paradoxical high expression of Caspase-8 in various cancer types. In my opinion, the most significant points in this paper are as follows: 1) Oncogenic KRAS-driven cancer has the vulnerability to the treatment to induce necroptosis induction. 2) Activation of STING/TBK1 signaling following oncogenic KRAS is key to triggering necroptosis. To strengthen these findings and before considering publication in a journal such as Nature Communications, several points should be addressed. I have listed my comments below.

Major points
１、Among various gene mutations related to the MAPK pathway, it is unclear how specifically oncogenic KRAS induces necroptotic priming. Oncogenic KRAS has been reported to stimulate innate immune signaling, in addition to the MAPK and AKT pathways, via binding to various effectors. However, as the authors argue in this paper, if addiction to the MAPK pathway including BRAF mutations is crucial, it is possible that not only KRAS but also other RAS mutations, EGFR, and MET mutations could induce necroptotic priming. From this perspective, expanding the analyses of TCGA in Figures 5I and 5J or using another human cancer database, it is necessary to examine which gene mutations frequently occur in tumors with high necroptosis scores, and discuss the specificity of oncogenic KRAS. This is important for determining which patients with specific gene mutations should be targeted by inducing necroptosis, as the authors propose.

２、The mechanism by which oncogenic KRAS activates STING remains unclear. This is particularly important for the novelty of this paper, since aside from the KRAS-STING axis, many of the signaling cascades shown in Figure 4J are combinations of well-known pathways. Although the authors conducted several experiments related to the release of mtDNA and immunogenic micronuclei, the investigation is not thorough. Since it has been reported that treatment with CDK4/6 inhibitors also triggers the IFN pathway, this approach does not effectively verify the existence of immunogenic micronuclei. For example, they could potentially try detecting cGAS foci or leaked mtDNA in the cytoplasm, cGAS depletion, detecting the markers of various DNA damage following oncogenic KRAS activation in thier system. While it is not necessary to perform all of these experiments, given that this is novel aspect of this study, a more detailed analysis should be performed to characterize this mechanism.

３、As the authors focus on CD45+ cells infiltration and the activation of the IFN pathway in bystander TME components, recent studies have reported that immunogenic cell death of cancer cells associated with necroptosis contributes to the activation of dendritic cells, which are essential for the initiation of anti-tumor immunity. It is clinically important to investigate whether MLKL-dependent necroptosis not only causes cell-autonomous cell death but also contributes to tumor regression through anti-tumor immunity in PDAC, in which resistance to ICIs remains a significant clinical challenge. For example, it would be valuable to examine the effect of administering CD8-neutralizing antibodies in the KC-C8FL/FL model.

４、In Figure 6, does the induction of necroptosis in the organoids of KRAS-mutant PDAC get suppressed by STING deletion or treatment with STING inhibitor? Since most experiments demonstrating STING dependency in inducing necroptosis pathway were performed in MEFs, it would be valuable to validate the role of STING in the context of KRAS-driven pancreatic cancer.

５、Recently, many studies have reported that the administration of KRAS inhibitors including KRASG12C and KRASG12D inhibitors promotes the remodeling of the tumor immune microenvironment to 'hot' state through the secretion of various cytokines/chemokines, including type I IFN. It would be valuable to include a discussion comparing these contrasting results with the findings of this study, which show that the IFN pathway is activated by oncogenic KRAS via STING signaling. This could provide significant insights in this field.

Reviewer #2

(Remarks to the Author)
In this study elevated caspase-8 is associated with PDAC, with the authors positing that this acts to restrain necroptotic killing. The authors show that oncogenic KRAS activates STING/TBK1 to induce type I IFNs that prime cells for necroptosis via increasing ZBP1 and MKLK expression. The study is well conducted and is, to my knowledge, novel, and the authors conclusions supported by the presented data. I only have a few requests requiring clarification:

1. Figure legends should clearly define how many independent experiments were performed in order to validate each data set.
3. The authors correlate oncogenic RAS induced type I IFN and subsequent MLKL and ZBP1 expression as sensitising to necroptosis. In my opinion it is important to substantiate these in vitro and/or correlative data by examining if blocking type I IFN signalling in vivo recapitulates the effects of MLKL deletion in KC-C8 fl/fl mice, and test if necroptosis engaged upon caspase-8 deletion (KC-C8 fl/fl) is driven by ZBP1?
4. Line 215/216: Please define the experimental endpoint.
5. Fig. S3B is important and should be included in the main figures with mouse numbers and parameters defining the ethical endpoint clearly noted.
6. Fig. S3K. Does G12D expression increase caspase-8 levels to explain the sensitization to caspase-8 activation i.e. did this come up in the bulk RNA seq (Fig. 3)?
7. Caspase-10 can act redundantly with caspase-8 in blocking necroptotic signalling in human cells. As such, are elevated caspase-10 levels also observed in the PAAD TCGA, and is there any correlation with survival (e.g. Fig. 1A,B)?

Reviewer #3

(Remarks to the Author)
In this study Tishina et al. report that high level of caspase 8 expression in PDAC tumors is protective against necroptosis. Furthermore, they suggest that KRAS-driven STING-dependent type I interferon response leads to necroptotic priming and sensitization to cell death. The role of Caspase 8/MLKL/ ZBP1 axis in controlling the balance between necroptosis and apoptosis is well-established in PDAC as well as other malignancies. The potentially novel aspect of the work is related to the KRAS-dependent priming mechanism that relies on the engagement of type I interferon response. However, this aspect of the study has several weaknesses that collectively detract from the strength of the conclusion drawn. Therefore, the work in its present form is not suitable for publication in Nature Communications.

Major comments:
1. Overall the review process has been hindered by the fact that the manuscript reads like an advanced draft (acronyms are not defined in order of appearance or at all, misplaced cut and paste content, etc.) Thorough editing would improve the reading experience.

2. The central mechanistic tenant of the study hinges on the claim that KRAS drives STING-dependent type I interferon response. This postulate is not aligned with earlier studies documenting that Type 1 interferon pathway is actively repressed in PDAC cells as a consequence of KRAS signaling (Mutahalagu et al. Cancer Discovery 2020; Vonderhaar et al. Cell Mol Gastro hepatology, 2021)

3. Line 207: The authors claim that specific staining reagents for phosphorylated MLKL are not available. This claim is not supported by published data. For example, Samson et al, Cell Death Differ 2021 describe IF and IHC staining protocols for RIPK1, RIPK3 and MLKL and their phosphorylated forms. Furthermore, Kelepouras et al., Nature 2024 recently published and demonstrated an optimal protocol for staining p-MLKL specifically in mice. This staining data is critical for the substantiation of the author's conclusion regarding the specific cell death mechanism driving the observed phenotype.

4. Line 193: The rationale for implicating necroptosis based on the observed neutrophil accumulation in the C8 K/O mice is not supported by the data. The marker used to define neutrophils CD11b+Gr1+ (Line 186: Figure S2) is inclusive of other cell populations making it difficult to attribute the increase in immune infiltrates selectively to neutrophils.

5. There is unexplained inconsistency in treatment conditions applied to different cell lines (Figure S3I/J). Furthermore, the staining approach used to ascertain co-localization is inadequate as it relies on two different staining techniques IHC and IF and therefore spatial overlap cannot be discerned (Figure S8D).

6. A large portion of the in-vitro studies (documenting cell death, necroptosis related protein/RNA expression etc.) were carried out using MEFs. Given the centrality of these data to the overall conclusions of the manuscript, and the well-recognized importance of cellular context in determining the nature of biological responses, the in vitro analyses should be carried out in pancreatic cancer cells.

7. The limited scope of the in vivo experiments weakens the significance of the results and their interpretation. For example, comparison of tumor burden between treatment and control cohorts is not included. Characterization of changes in cell death after treatment with emricasan/birinapant (ES) combination as well as exploration of the tumor immune environment would further strengthen associations between Cas8 inhibition and necroptosis.

Minor comments:
-Line 97-98: Misplaced sentence? Accidentally dragged over from introduction?
-Line 133: figure S1F lacks representative IHC plot (why needed)
-Line 204: the authors mention the “very thick CK19+ ductal linings with very large ductal lesions”. A representative image would strengthen the statement
-Line 218: Figure 2F and 2G: Statistical analysis is missing
-Figure 3B: line 1298: Negative enrichment score should be replaced by normalized enrichment score
-Figure S7I Statistical analysis is missing
-Line 421: Spelling mistake “bot” but
-Figure S8: Says related to figure 5 but its related to figure 6

Version 2:

Reviewer comments:

Reviewer #1

(Remarks to the Author)
The authors have presented a sufficient amount of convincing data addressing my comments. I have no additional comments.

Reviewer #2

(Remarks to the Author)
The reviewers have done a superb job in answering my queries. I'd like to congratulate them on a very nice story that I believe will garner significant attention in the necroptosis/cancer fields.

Reviewer #3

(Remarks to the Author)
Following a careful review of the authors' response to the points we have raised we have, we reached to conclusion that all comments and concerns have been adequately addressed. Therefore, publication of the manuscript in its present form is recommended.

Open Access This Peer Review File is licensed under a Creative Commons Attribution 4.0 International License, which permits use, sharing, adaptation, distribution and reproduction in any medium or format, as long as you give appropriate credit to the original author(s) and the source, provide a link to the Creative Commons license, and indicate if changes were made.
In cases where reviewers are anonymous, credit should be given to 'Anonymous Referee' and the source.
The images or other third party material in this Peer Review File are included in the article's Creative Commons license, unless indicated otherwise in a credit line to the material. If material is not included in the article's Creative Commons license and your intended use is not permitted by statutory regulation or exceeds the permitted use, you will need to obtain permission directly from the copyright holder.
To view a copy of this license, visit https://creativecommons.org/licenses/by/4.0/

Tishina et al. point_by_point_reply:

Reviewer #1 (Remarks to the Author)

In this manuscript, Tishina and colleagues focused on the higher expression of Caspase-8, which initiates the apoptosis cascade, in KRAS-dependent PDAC and is associated with a rather poorer prognosis in patients. Utilizing a sophisticated murine PDAC model combined with deletion of Caspase-8 and/or MLKL, they demonstrated that Caspase-8 functions as a tumor suppressor by inhibiting MLKL-induced necroptosis. This provides compelling evidence that the suppression of necroptosis by Caspase-8 contributes to pancreatic cancer progression. In addition, they showed that the STING/TBK1 pathway and subsequent autocrine type I IFN signaling activated by oncogenic KRAS is crucial for inducing the molecules driving necroptosis, such as MLKL and ZBP1. While it is well-known that Caspase-8 inhibits necroptosis, this study shed light on the novel aspects of the paradoxical high expression of Caspase-8 in various cancer types. In my opinion, the most significant points in this paper are as follows: 1) Oncogenic KRAS-driven cancer has the vulnerability to the treatment to induce necroptosis induction. 2) Activation of STING/TBK1 signaling following oncogenic KRAS is key to triggering necroptosis. To strengthen these findings and before considering publication in a journal such as Nature Communications, several points should be addressed. I have listed my comments below.

Major points

１、Among various gene mutations related to the MAPK pathway, it is unclear how specifically oncogenic KRAS induces necroptotic priming. Oncogenic KRAS has been reported to stimulate innate immune signaling, in addition to the MAPK and AKT pathways, via binding to various effectors. However, as the authors argue in this paper, if addiction to the MAPK pathway including BRAF mutations is crucial, it is possible that not only KRAS but also other RAS mutations, EGFR, and MET mutations could induce necroptotic priming. From this perspective, expanding the analyses of TCGA in Figures 5I and 5J or using another human cancer database, it is necessary to examine which gene mutations frequently occur in tumors with high necroptosis scores, and discuss the specificity of oncogenic KRAS. This is important for determining which patients with specific gene mutations should be targeted by inducing necroptosis, as the authors propose.

We appreciate this constructive suggestion and have performed the additional dataset analysis requested. Strikingly, oncogenic KRAS mutations showed by far the strongest association with necroptosis score in a pan-cancer analysis, displaying both the highest necroptosis score and an extraordinarily significant correlation (q = 1.4e10-96). In contrast, although several other mutations or amplifications exhibited mildly elevated necroptosis scores on average, these associations were not statistically significant. The only exception was BRAF-mutated cancers, which showed a significant but much weaker correlation (q = 0.01) and a far less uniform distribution compared to the KRAS-mutant group (new Supplementary Figure 3h). Based on this analysis but also our prior identification of ISG signatures and transcriptional Ras pathway activation to best correlate with necroptotic priming, we propose to use transcriptional signatures of ISG score or Ras pathway activation for classifying potential necroptosis responders rather than mutation status.

２、The mechanism by which oncogenic KRAS activates STING remains unclear. This is particularly important for the novelty of this paper, since aside from the KRAS-STING axis, many of the signalling cascades shown in Figure 4J are combinations of well-known pathways. Although the authors conducted several experiments related to the release of mtDNA and immunogenic micronuclei, the investigation is not thorough. Since it has been reported that treatment with CDK4/6 inhibitors also triggers the IFN pathway, this approach does not effectively verify the existence of immunogenic micronuclei. For example, they could potentially try detecting cGAS foci or leaked mtDNA in the cytoplasm, cGAS depletion, detecting the markers of various DNA damage following oncogenic KRAS activation in their system. While it is not necessary to perform all of these experiments, given that this is novel aspect of this study, a more detailed analysis should be performed to characterize this mechanism.

We very much appreciate the reviewer's suggestion to further explore how oncogenic KRAS activates STING. To address this, we performed additional experiments directly assessing the involvement of the cGAS–STING pathway in our system.

We first observed that expression of oncogenic KRAS was associated with increased accumulation of cytosolic micronuclei, a hallmark of cytosolic DNA stress, which was ablated upon $KRAS^{G12D}$ inhibition (new Fig. 3a, b). Importantly, this response was independent of mitochondrial DNA, as experimental depletion of mitochondrial DNA did not alter KRAS-induced ISG expression, excluding mtDNA as a major source of immunostimulatory DNA in this context (new Supplementary Fig. 4a, b). In addition, consistent with a role for cytosolic DNA sensing, we detected robust STING phosphorylation upon KRAS expression (new Fig. 3c, Supplementary Fig. 4c).

To directly test the requirement for cGAS, we silenced cGAS and found that oncogenic KRAS-induced ISG expression was entirely ablated, similarly as with Sting silencing (new Fig. 3d-g, Supplementary Fig. 4d), demonstrating that ISG upregulation is dependent on cGAS-mediated DNA sensing.

Notably, although γH2AX was detectable upon oncogenic KRAS expression, its signal increased subsequent to STING phosphorylation, suggesting that DNA damage follows STING activation rather than preceding it in this context (new Fig. 3c, Supplementary Fig. 4c).

Taken together, these data indicate that $KRAS^{G12D}$ expression and activity promote cytosolic DNA accumulation, leading to activation of a cGAS–STING–TBK1–dependent type I interferon response. We appreciate the reviewer's constructive feedback, which enabled us to further define the mechanism underlying KRAS-mediated STING activation.

３、As the authors focus on CD45+ cells infiltration and the activation of the IFN pathway in bystander TME components, recent studies have reported that immunogenic cell death of cancer cells associated with necroptosis contributes to the activation of dendritic cells, which are essential for the initiation of anti-tumor immunity. It is clinically important to investigate whether MLKL-dependent necroptosis not only causes cell-autonomous cell death but also contributes to tumor regression through anti-tumor immunity in PDAC, in which resistance to

ICIs remains a significant clinical challenge. For example, it would be valuable to examine the effect of administering CD8-neutralizing antibodies in the KC-C8FL/FL model.

We appreciate this important point regarding the potential contribution of MLKL-dependent necroptosis to anti-tumour immunity in PDAC. To address this, we performed additional experiments assessing whether the therapeutic efficacy of necroptosis induction is promoted by T cells. To specifically test a clinically relevant scenario, we evaluated the combination of the necroptosis-inducing regimen (caspase inhibitor Emricasan together with Smac mimetics; ES) and anti-PD-1 treatment in KPC mice in vivo. Interestingly, the addition of anti-PD-1 did not further enhance therapeutic benefit and in fact caused some toxicity, which may account for the slightly inferior outcome compared to ES monotherapy (panel A). These observations suggest that in this cancer model the anti-tumour effects of ES do not rely on CD8⁺ T-cell–mediated immunity. For reasons of keeping a concise message within the manuscript, we have opted to not include the data into the manuscript but are showing them to the reviewer here.

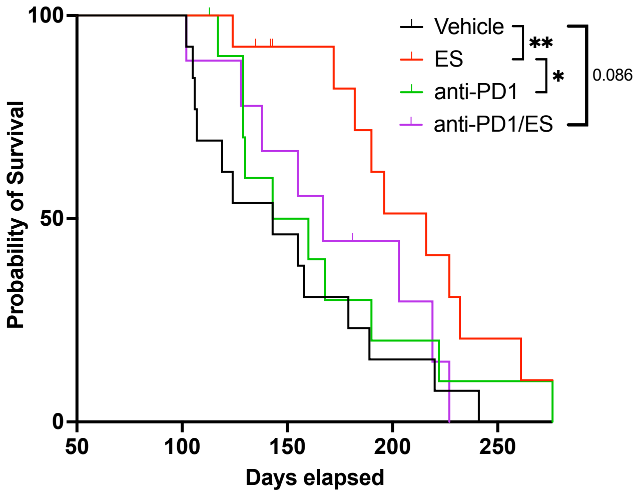

Panel A. 8-week-old KPC mice were treated with vehicle (PBS containing 40% PEG-4000 and 0.4% DMSO) or a combination of emricasan [2.5 mg/kg] and birinapant [5 mg/kg] administered twice weekly for four consecutive weeks, with or without anti-PD-1 antibody in PBS [5 mg/kg], which was administered for three consecutive weeks. Group sizes were as follows: vehicle (n = 13), emricasan/birinapant (n = 13), anti-PD-1 (n = 11), and anti-PD-1 with emricasan/birinapant (n = 9). Mice were monitored for Kaplan–Meier survival analysis until the experimental endpoint was reached.

４、In Figure 6, does the induction of necroptosis in the organoids of KRAS-mutant PDAC get suppressed by STING deletion or treatment with STING inhibitor? Since most experiments demonstrating STING dependency in inducing necroptosis pathway were performed in MEFs, it would be valuable to validate the role of STING in the context of KRAS-driven pancreatic cancer.

We fully agree with this point. To address it we made use of mouse KC-organoids which have been shown to closely mimic PDAC in vivo[1]. Indeed, in new data added, STING inhibition fully reverted the necroptosis sensitivity in KC-organoids ex vivo (new Fig. 5h, i).

５、Recently, many studies have reported that the administration of KRAS inhibitors including KRASG12C and KRASG12D inhibitors promotes the remodelling of the tumour immune microenvironment to 'hot' state through the secretion of various cytokines/chemokines, including type I IFN. It would be valuable to include a discussion comparing these contrasting results with the findings of this study, which show that the IFN pathway is activated by oncogenic KRAS via STING signalling. This could provide significant insights in this field.

Following the reviewer's suggestion, we have now expanded the Discussion regarding this point. While KRAS inhibition has been shown to enhance IFN-related programs in vivo, these effects frequently occur in the context of oncogene addiction, where acute KRAS blockade induces tumour cell stress and immunogenic cell death, thereby secondarily activating immune cells that serve as the dominant source of cytokine and chemokine production[2,3]. In addition, KRAS-G12C inhibition causes remodelling of the tumour immune microenvironment and synergizes with anti–PD-1 therapy predominantly in tumours with pre-existing T cell infiltration, underscoring that the response is largely shaped by immune cell dynamics rather than tumour-intrinsic signalling alone[3]. Moreover, off-target effects of KRAS inhibitors on normal cells may also contribute to these observations, as exemplified by sotorasib binding to KEAP1 and inhibiting its enzymatic activity[4].
In contrast, our study addresses the tumour-intrinsic role of oncogenic KRAS, demonstrating that active KRAS directly induces type I IFN signalling through STING activation. Importantly, analysis of our KC-derived single-cell RNA-seq data revealed that bona fide interferon-stimulated genes (ISGs) were strongly enriched in ductal PanIN cells but not in normal acinar cells (Fig. 2c). Furthermore, consistent with these findings, independent patient single-cell datasets show higher ISG expression in human PDAC compared with normal pancreas (Fig. 2f-k). Further analysis of TCGA revealed that ISG levels correlate with the Ras84 score and that KRAS-mutant cancers (including PDAC, Lung adenocarcinoma (LUAD) and Colon adenocarcinoma (COAD)) rank among those with the highest ISG expression (Fig. 2l). Together, these observations suggest that, although KRAS inhibition and oncogenic KRAS activation can both be associated with IFN signalling, they likely do so through distinct systemic mechanisms. We propose that based on our data in line with data from pan cancer TCGA and PDAC patient single cell data analysis, KRAS inhibition may primarily promote IFN responses indirectly via immune cell activation and tumour cell death in vivo, while our data reveal a previously underappreciated role for oncogenic KRAS itself in directly engaging the STING–type I IFN axis in tumour cells. Thus, rather than being contradictory, these findings highlight the context-dependent and multilayered regulation of IFN signalling in KRAS-driven cancers.

Reviewer #2 (Remarks to the Author)

In this study elevated caspase-8 is associated with PDAC, with the authors positing that this acts to restrain necroptotic killing. The authors show that oncogenic KRAS activates

STING/TBK1 to induce type I IFNs that prime cells for necroptosis via increasing ZBP1 and MKLK expression. The study is well conducted and is, to my knowledge, novel, and the authors conclusions supported by the presented data. I only have a few requests requiring clarification:

1. Figure legends should clearly define how many independent experiments were performed in order to validate each data set.

We fully agree with data transparency. Each figure legend indicates the number of mice used in the experiment. In all cases means calculated from at least three biological replicates are shown. Biological replicates on any given day were performed with at least two technical replicates. Dots within bar diagrams represent biological replicates or mice if not stated otherwise. Additionally, to accommodate all the different types of repeats (number of independent cohorts, number of independent biological repeats) without stretching figure legends too much we have dedicated a section in the methods "Statistical Analysis" to this clarifying this throughout and reported on this in detail in the reporting summary.

3. The authors correlate oncogenic RAS induced type I IFN and subsequent MLKL and ZBP1 expression as sensitising to necroptosis. In my opinion it is important to substantiate these in vitro and/or correlative data by examining if blocking type I IFN signalling in vivo recapitulates the effects of MLKL deletion in KC-C8 fl/fl mice, and test if necroptosis engaged upon caspase-8 deletion (KC-C8 fl/fl) is driven by ZBP1?

Although desirable, unfortunately, to genetically show the full pathway in vivo in this model would require generating a quadruple transgenic hemizygous mouse line (e.g. LsLKRASG12D$^{+/wt}$;PDX1-Cre$^{+/wt}$;Casp8 $^{FL/FL}$; ifnar1$^{FL/FL}$- 6% expected Mendelian ratio to be born in the best case scenario) followed by prolonged aging (5 month), which is not feasible within the scope of this study. Therefore, in order to address the reviewer's point and given the close recapitulation of KC organoids of PanINs [1] we performed additional experiments on KC-organoids as was also suggested by reviewer 1. Indeed, in newly added data, inhibition of STING, which is upstream of type I interferon signalling, completely reversed the necroptosis sensitivity in KC-organoids ex vivo (new Fig. 5h, i).

To first assess a potential role of ZBP1 in necroptosis execution in mouse PDAC, we first tested ZBP1 mRNA expression in PanIN cells sorted ex vivo from KRAS-expressing PanIN cells, using the same approach as for caspase-8 in Figure 1c. Surprisingly, despite being a type I IFN target gene, Zbp1 expression in GFP^{+} PanIN cells harbouring KRAS^{G12D} was markedly lower than in the adjacent GFP^{-} normal tissue for 5-months-old KC mice (new Supplementary Figure 7f). Additionally, we also performed bulk qPCR analysis on whole pancreatic tissue from KC mice at various timepoints, as well as from KPC tumours. In this analysis, Zbp1 expression was detectable only at advanced stages, specifically in KPC tumours, but not during earlier stages in KC mice (new Supplementary Figure 7e). These data suggest that in early stages ZBP1 expression might be counter-selected through low level constitutive necroptosis and its expression is only tolerated at later stage. While we appreciate that understanding the role of ZBP1 in PDAC will be important, its described cell death-independent function in driving a type

I IFN response itself[5] will require a separate in depth investigation to disentangle this effect from the KRAS-driven effect which is outside the scope of this study.

4. Line 215/216: Please define the experimental endpoint.

An explanation of experimental endpoint has been added the methods section "mice". In brief experimental endpoints were determined based upon a scoring system quantifying animal well-being based upon weight loss, general condition, abnormal behaviour as common practice in German animal experimentation. Animals were euthanized once the cumulative score reached 20. Scoring was done by a person blinded to the study.

5. Fig. S3B is important and should be included in the main figures with mouse numbers and parameters defining the ethical endpoint clearly noted.

Fig. S3B has been moved to new Figure 1j. An explanation of experimental endpoint has been added the methods section "mice" and as described above.

6. Fig. S3K. Does G12D expression increase caspase-8 levels to explain the sensitization to caspase-8 activation i.e. did this come up in the bulk RNA seq (Fig. 3)?

We did not observed upregulation of caspase 8 upon KRAS induction in any inducible KRAS expression model (MEFs or HPDE or BxPC-3 cells or mPDAC). We think that upregulation of caspase 8 is secondary or a result of selection, which serves as protection for cancer cells with upregulated necroptosis pathway components such as pancreatic cancer cell lines (Supplementary Fig. 6l), pancreatic cancer mouse models (Fig. 1c-d, 1g, 5g, Supplementary Fig. 1h, 2d, 7e) and PDAC patients (Fig. 1a-b, 2l, m, 5e, Supplementary Fig. 1a-c).

7. Caspase-10 can act redundantly with caspase-8 in blocking necroptotic signalling in human cells. As such, are elevated caspase-10 levels also observed in the PAAD TCGA, and is there any correlation with survival Caspase-10 can act redundantly with caspase-8 in blocking necroptotic signalling in human cells. As such, are elevated caspase-10 levels also observed in the PAAD TCGA, and is there any correlation with survival (e.g. Fig. 1A,B)?

This is a very interesting suggestion which we followed up on. Indeed, CASP10 expression is significantly elevated in the TCGA PDAC patient cohort and shows a strong positive correlation with the Ras84 score (r=0.85), whereas normal pancreatic tissue exhibits substantially lower levels (new Supplementary Figure 2p). Moreover, patients in the 10th percentile of the lowest CASP10 expression formed a distinct subgroup of long-term PDAC survivors, supporting the notion that reduced CASP10 expression may be associated with improved outcome (new Supplementary Figure 2q). Given that CASP10, similar to CASP8, can inhibit necroptosis in human cells, these findings further independently support the hypothesis that elevated levels of necroptosis protectors such as CASP8 and CASP10 may be advantageous for sustaining pancreatic cancer by limiting necroptotic cell death. We want to thank the reviewer for raising this point as it has significantly contributed to the scope of our findings.

Reviewer #3 (Remarks to the Author):

In this study Tishina et al. report that high level of caspase 8 expression in PDAC tumors is protective against necroptosis. Furthermore, they suggest that KRAS-driven STING-dependent type I interferon response leads to necroptotic priming and sensitization to cell death. The role of Caspase 8/MLKL/ ZBP1 axis in controlling the balance between necroptosis and apoptosis is well-established in PDAC as well as other malignancies. The potentially novel aspect of the work is related to the KRAS-dependent priming mechanism that relies on the engagement of type I interferon response. However, this aspect of the study has several weaknesses that collectively detract from the strength of the conclusion drawn. Therefore, the work in its present form is not suitable for publication in Nature Communications.

Major comments:
1. Overall the review process has been hindered by the fact that the manuscript reads like an advanced draft (acronyms are not defined in order of appearance or at all, misplaced cut and paste content, etc.) Thorough editing would improve the reading experience.

We apologise for this and have made every effort to improve readability and flow of the manuscript in the revised version.

2. The central mechanistic tenant of the study hinges on the claim that KRAS drives STING-dependent type I interferon response. This postulate is not aligned with earlier studies documenting that Type 1 interferon pathway is actively repressed in PDAC cells as a consequence of KRAS signalling (Mutahalagu et al. Cancer Discovery 2020; Vonderhaar et al. Cell Mol Gastro hepatology, 2021)

This is indeed an important point. Previous studies have suggested that oncogenic KRAS can dampen type I interferon (IFN-I) signalling in pancreatic cancer models, and we have now addressed this more explicitly in the Discussion. To this end, we carefully re-examined the experimental setups of the cited studies in relation to our own.
In Vonderhaar et al.[6], the authors demonstrate that pharmacological activation of STING can promote tumour cell demise in a manner dependent upon tumour cell expressed ifnar1. This aligns very well with our finding that STING drives up ifnar1-dependent expression of MLKL and ZBP1. While in the endogenous KRAS-driven scenario necroptosis execution is kept in check by caspase 8, this may be overridden when pushing type I IFN expression to the limits using STING agonists. It is therefore tempting to speculate whether in this paper STING agonists induce necroptosis in PDAC, and therefore oblation of the tumour is observed.

Muthalagu et al.[7] reported KRAS-dependent suppression of interferon signalling in their experimental system including MYC oncogene expression. To address this apparent discrepancy, we repeated the key experiment using wild-type and LSL-KRAS^{G12D} MEFs infected with adeno-Cre under conditions analogous to those described in the original study. In our hands, KRAS activation did not diminish the interferon response: both wild-type and KRAS^{G12D} MEFs exhibited comparable ISG induction 24 hours after viral exposure. One notable difference between the two systems is that KRAS-mutant cells in Muthalagu et al. expressed high levels of MYC, whereas MYC expression is relatively low in our MEFs, as determined by

RNA-seq. As shown in that study and others, MYC can mediate repression of interferon responses, which may explain why KRAS signalling appeared inhibitory in their system but not in ours.

An additional consideration is that adenoviral exposure itself induces a strong innate immune response, including robust type I IFN signalling that can persist for at least 24 hours post-infection, which corresponds to the experimental harvesting time point[8]. Under these conditions, virus-driven IFN programs may dominate the transcriptional landscape and obscure more subtle KRAS-dependent effects, potentially making KRAS-mutant cells appear comparatively less responsive to virus-induced interferon signalling.

To further assess the relevance of our findings in physiologically relevant settings, we analysed KC-derived single-cell RNA-seq data and observed that bona fide ISGs were markedly enriched in ductal (KRAS-driven PanIN) cells but not in normal acinar cells (new Fig. 2c). Consistent with this, independent patient single-cell datasets show higher ISG expression in human PDAC compared with normal pancreas (new Fig. 2f-k). Moreover, analysis of TCGA datasets revealed that ISG levels strongly and positively correlate with the Ras84 score, and that KRAS-mutant cancers, including PDAC, lung adenocarcinoma (LUAD), and colon adenocarcinoma (COAD), rank among those with the highest ISG expression (Fig. 2l). Collectively, these observations indicate that while the influence of oncogenic KRAS on IFN-I signalling may vary across in vitro experimental systems and may depend on co-mutations, our findings align well with patterns observed in patient-derived data.

3. Line 207: The authors claim that specific staining reagents for phosphorylated MLKL are not available. This claim is not supported by published data. For example, Samson et al, Cell Death Differ 2021 describe IF and IHC staining protocols for RIPK1, RIPK3 and MLKL and their phosphorylated forms. Furthermore, Kelepouras et al., Nature 2024 recently published and demonstrated an optimal protocol for staining p-MLKL specifically in mice. This staining data is critical for the substantiation of the author's conclusion regarding the specific cell death mechanism driving the observed phenotype.

As requested, we have included TUNEL stains yet, very few dead cells are detected overall at time of analysis as pancreatic tissue is known to be rapidly remodeled and PDX1-Cre is expressed already pre-birth. Thereby, we assume to have missed the time at which cell death takes place (possible pre-birth) with these tissue stains in the genetic model. In KC mice treated with Emricasan/birinapant we have included TUNEL stains and cl. Casp3 staining and indeed detected cells that are TUNEL positive but cl. C3 negative (Supplementary Figure 7d). Given that an MLKL staining protocol has recently become available for other tissues[9], we also tried optimising pMLKL staining for the mouse pancreas in collaboration with the authors of the cited study (Kelepouras et al.) yet could not obtain specific conclusive results for the pancreas. For this reason, we chose to isolate murine KC-derived pancreatic organoids from +/- caspase 8 and treated them with TNF/smac mimetic ex vivo. Indeed, KC-casp8 floxed pancreatic organoids undergo TNF/smac mimetic induced necroptosis while KC-casp8 WT organoids do not (new Figure 1h, i). Western blot analysis confirmed the presence of pMLKL in this system, indicating activation of the necroptotic pathway in these organoid models which closely mimic the biology of PanIN lesions of KC-C8 FL mice (new Figure 1g).

4. Line 193: The rationale for implicating necroptosis based on the observed neutrophil accumulation in the C8 K/O mice is not supported by the data. The marker used to define neutrophils CD11b+Gr1+ (Line 186: Figure S2) is inclusive of other cell populations making it difficult to attribute the increase in immune infiltrates selectively to neutrophils.

The reviewer raises an important point as the markers CD11b and Gr1 are not only specific to neutrophils. To be more precise, we repeated the staining using additional markers and gating strategies to exclude other populations that share expression of CD11b and/or Gr1 like eosinophils (CCR3+), monocytes (CD14+) and CD11b+SSClow ones (potential NK, B or other myeloid cells). As such, neutrophils are identified now as CD45+/CD14-CCR3-/CD11b+ SSChigh/Gr1+ cells (gating strategy in Panel B). The new results are shown in Supplemental Figure 2m.

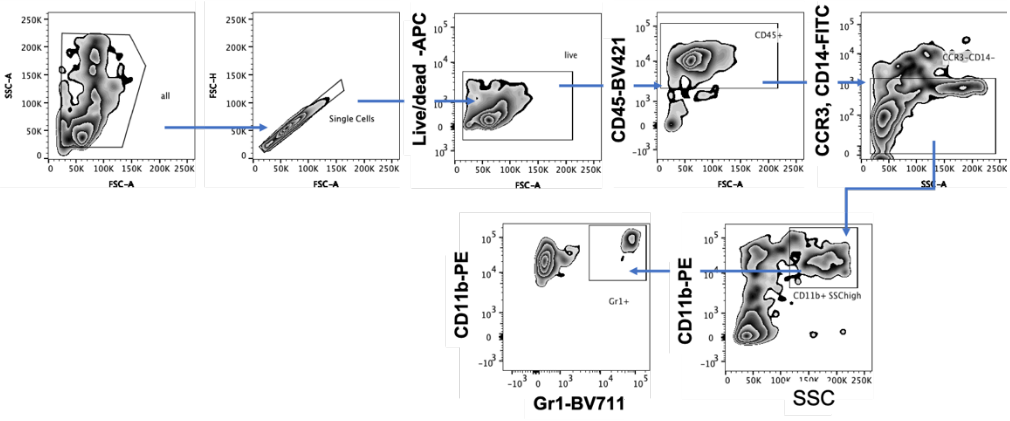

Panel B. New gating strategy identifying neutrophils as $CD45^{+}/CD14^{-}CCR3^{-}/CD11b^{+}SSC^{high}/Gr1^{+}$ *cells.*

5. There is unexplained inconsistency in treatment conditions applied to different cell lines (Figure S3I/J). Furthermore, the staining approach used to ascertain co-localization is inadequate as it relies on two different staining techniques IHC and IF and therefore spatial overlap cannot be discerned (Figure S8D).

In Figure S3I/J we are showing that human and mouse PDAC cells show cleaved caspase 3 activation upon stimulation with TNF/Smac mimetic and RIPK3 inhibition. We are not sure as to the mentioned inconsistency as we have merely added one more condition without RIPK3 inhibition to S3J. Given that these data are not central to the scope of the manuscript, we are also happy to remove them. Regarding IHC and IF in Figure S8D, we corrected the text claim so that it is clear that we do not claim overlap as it is indeed not the same slide but rather consecutive slides analysed.

6. A large portion of the in-vitro studies (documenting cell death, necroptosis related protein/RNA expression etc.) were carried out using MEFs. Given the centrality of these data to the overall conclusions of the manuscript, and the well-recognized importance of cellular context in determining the nature of biological responses, the in vitro analyses should be carried out in pancreatic cancer cells.

We fully agree with this statement. To this end, we now have included necroptosis analysis into figure 1g-i using KC pancreatic organoids closely mimicking the biology of KRAS-driven pancreatic neoplasia, inducible mPDAC cells (new Figure 2e), human scRNA-seq data from patient samples (new Figure 2f-n), human pancreatic ductal epithelial cells (HPDE) and BxPC-3 with inducible KRASG12D (Supplementary Figure 3f and g), as well as RNA-seq data and killing of 12 human established pancreatic cell lines (Fig. 5d, Supplementary Figures 6k, l). For an even more clinically relevant model, we have now also tested the efficacy of necroptotic killing in human PDAC patient-derived organoids (PDO) (new Figure 6i-m). Together, we believe that we now provide comprehensive evidence that necroptotic priming also extends to pancreatic cancer cells.

7. The limited scope of the in vivo experiments weakens the significance of the results and their interpretation. For example, comparison of tumor burden between treatment and control cohorts is not included. Characterization of changes in cell death after treatment with emricasan/birinapant (ES) combination as well as exploration of the tumor immune environment would further strengthen associations between Cas8 inhibition and necroptosis.

We appreciate the reviewer's careful evaluation. We note that we have presented tumour burden of control and treated KC mice by pathological analysis and ck19 staining quantification (Figure 6a-f) yet, the request for direct comparison of tumour burden in the KPC mouse model does not fully take into account the highly variable timing of tumour initiation and progression between individual animals. This intrinsic heterogeneity precludes meaningful tumour-burden comparisons at fixed time points in most laboratory settings. Accordingly, survival analyses and mechanistic endpoints are considered robust and informative measures of therapeutic efficacy in the KPC model, and we have therefore focused on these readouts and observe a -for this model- drastic improvement of median survival.

To nevertheless further address this point of the reviewer, we now provide an even more clinically relevant model, using human PDAC patient-derived organoids (PDO) (new Figure 6i-m) and equally observe PDO killing that is dependent upon RIPK1 and caspase inhibition, and hence, necroptosis. Together, our findings provide preclinical evidence that therapeutic induction of necroptosis may represent a promising strategy for targeting pancreatic cancer.

Minor comments:

-Line 97-98: Misplaced sentence? Accidentally dragged over from introduction?
Thank you for pointing this out, we removed this misplaced sentence from the revised manuscript.

-Line 133: figure S1F lacks representative IHC plot (why needed)
We included a representative IHC plot to new Figure 1e and quantifications of the sections can be found in new Supplementary Figure 1f.

-Line 204: the authors mention the "very thick CK19+ ductal linings with very large ductal lesions". A representative image would strengthen the statement

A representative IHC section has been added to the CK19 panel of the new Figure 1e, and the text has been updated to reference this panel accordingly.

-Line 218: Figure 2F and 2G: Statistical analysis is missing
Now we included all the used statistical analysis in the end of each figure legend.

-Figure 3B: line 1298: Negative enrichment score should be replaced by normalized enrichment score
We thank the reviewer and corrected this typo.

-Figure S7I ◊ Statistical analysis is missing
Thank you for pointing this out, now we included all the used statistical analysis in the end of each figure legend.

-Line 421: Spelling mistake "bot" ◊ but
We corrected this typo.

-Figure S8: Says related to figure 5 but its related to figure 6
We corrected this wrong reference.

References

1. Boj, S. F. *et al.* Organoid Models of Human and Mouse Ductal Pancreatic Cancer. *Cell* **160**, 324–338 (2015).
2. Canon, J. *et al.* The clinical KRAS(G12C) inhibitor AMG 510 drives anti-tumour immunity. *Nature* **56**, 779–21 (2019).
3. Cole, M. *et al.* Spatial multiplex analysis of lung cancer reveals that regulatory T cells attenuate KRAS-G12C inhibitor-induced immune responses. *Sci Adv* **10**, eadl6464 (2024).
4. Wang, Y. *et al.* Global profiling of AMG510 modified proteins identified tumor suppressor KEAP1 as an off-target. *iScience* **26**, 106080 (2023).
5. Jiao, H. *et al.* ADAR1 averts fatal type I interferon induction by ZBP1. *Nature* **607**, 776–783 (2021).
6. Vonderhaar, E. P. *et al.* STING Activated Tumor-Intrinsic Type I Interferon Signaling Promotes CXCR3 Dependent Antitumor Immunity in Pancreatic Cancer. *Cellular and Molecular Gastroenterology and Hepatology* **12**, 41–58 (2021).
7. Muthalagu, N. *et al.* Repression of the Type I Interferon Pathway Underlies MYC- and KRAS-Dependent Evasion of NK and B Cells in Pancreatic Ductal Adenocarcinoma. *Cancer Discovery* **10**, 872–887 (2020).
8. Minamitani, T., Iwakiri, D. & Takada, K. Adenovirus virus-associated RNAs induce type I interferon expression through a RIG-I-mediated pathway. *J Virol* **85**, 4035–4040 (2011).
9. Kelepouras, K. *et al.* The importance of murine phospho-MLKL-S345 in situ detection for necroptosis assessment in vivo. *Cell Death Differ* **31**, 897–909 (2024).

<u>Tishina et al. point_by_point_reply:</u>

Reviewer #1 (Remarks to the Author):

The authors have presented a sufficient amount of convincing data addressing my comments. I have no additional comments.

Reviewer #2 (Remarks to the Author):

The reviewers have done a superb job in answering my queries. I'd like to congratulate them on a very nice story that I believe will garner significant attention in the necroptosis/cancer fields.

Reviewer #3 (Remarks to the Author):

Following a careful review of the authors' response to the points we have raised we have, we reached to conclusion that all comments and concerns have been adequately addressed. Therefore, publication of the manuscript in its present form is recommended.

We are delighted to have addressed all comments made by the reviewers previously and have now provided the remaining editorial requests in the revised files.
